# Deep learning predicts potential reassortments of avian H5N1 with human influenza viruses

**DOI:** 10.1093/nsr/nwaf396

**Published:** 2025-09-17

**Authors:** Jun-Qing Wei, Sen Zhang, Ya-Dan Li, Shu-Yang Jiang, Si-Rui Yan, Ying Liu, Yue-Hong Chen, Ye Feng, Xiao Ding, Yu-Chang Li, Xiao-Ping Kang, Wei Liu, Aiping Wu, Tao Jiang, Yigang Tong, Jing Li

**Affiliations:** State Key Laboratory of Pathogen and Biosecurity, Academy of Military Medical Science, Beijing 100071, China; BAICSM, State Key Laboratory of Green Biomanufacturing, College of Life Science and Technology, Beijing University of Chemical Technology, Beijing 100029, China; State Key Laboratory of Pathogen and Biosecurity, Academy of Military Medical Science, Beijing 100071, China; State Key Laboratory of Pathogen and Biosecurity, Academy of Military Medical Science, Beijing 100071, China; State Key Laboratory of Pathogen and Biosecurity, Academy of Military Medical Science, Beijing 100071, China; College of Basic Medical Sciences, Inner Mongolia Medical University, Hohhot 010107, China; College of Basic Medical Sciences, Inner Mongolia Medical University, Hohhot 010107, China; State Key Laboratory of Pathogen and Biosecurity, Academy of Military Medical Science, Beijing 100071, China; State Key Laboratory of Pathogen and Biosecurity, Academy of Military Medical Science, Beijing 100071, China; State Key Laboratory of Common Mechanism Research for Major Diseases, Suzhou Institute of Systems Medicine, Chinese Academy of Medical Sciences & Peking Union Medical College, Suzhou 215123, China; State Key Laboratory of Pathogen and Biosecurity, Academy of Military Medical Science, Beijing 100071, China; State Key Laboratory of Pathogen and Biosecurity, Academy of Military Medical Science, Beijing 100071, China; State Key Laboratory of Pathogen and Biosecurity, Academy of Military Medical Science, Beijing 100071, China; State Key Laboratory of Common Mechanism Research for Major Diseases, Suzhou Institute of Systems Medicine, Chinese Academy of Medical Sciences & Peking Union Medical College, Suzhou 215123, China; State Key Laboratory of Pathogen and Biosecurity, Academy of Military Medical Science, Beijing 100071, China; BAICSM, State Key Laboratory of Green Biomanufacturing, College of Life Science and Technology, Beijing University of Chemical Technology, Beijing 100029, China; State Key Laboratory of Pathogen and Biosecurity, Academy of Military Medical Science, Beijing 100071, China; College of Basic Medical Sciences, Inner Mongolia Medical University, Hohhot 010107, China

**Keywords:** adaptive reassortment, H5N1, polymerase gene, deep learning, genome embedding

## Abstract

Frequent infection cases with avian H5N1 influenza A viruses (IAVs) are posing pandemic risks of human/avian-reassorted IAVs. We aimed to build an attentional deep learning model named HAIRANGE, for predicting potentially human-adapted reassortment of H5N1 and human IAVs. A biologically relevant and non-pretrained embedder named Codon2Vec in HAIRANGE performed competitively in benchmarking against other embedders, such as ESM2, DNABERT2 and others, indicating a high association of genomic context with viral hosts or serotypes, for IAV RNA polymerase-related genes. HAIRANGE accurately predicted the adaptation of each polymerase-related gene and the adaptive polymerase-related gene reassortment with polymerase activity validated by *in vitro* reporting assay. Worryingly, an adaptive reassortment between avian H5N1 and human H3N2 IAVs was predicted by HAIRANGE and validated by polymerase activity assay. Summarily, HAIRANGE can predict adaptive IAV reassortment based on embedded genomic context. Current avian H5N1 IAV is posing pandemic potential via possible reassortment with human IAVs.

## INTRODUCTION

Avian influenza A virus (IAV) naturally inhabits wild aquatic waterfowl and shorebirds [1], occasionally causing spillover infection to mammals [2], and rarely but periodically launching human influenza pandemics [3]. IAV is highly diversified in its genome of eight-segmented RNA, due to gene reassortment and a high mutation rate [4]. The global distribution and density of domestic and wild birds expand the ecological interface at which avian influenza viruses can spill over into mammals [5,6]. The extensive genetic diversity that accumulates in these avian reservoirs increases the likelihood that some strains possess molecular traits compatible with mammalian entry or replication of IAVs [7]. After a spillover event, additional host-specific mutations or reassortments may adapt to the mammalian host, further enhancing virus infection and transmissibility [8]. Adaptation of viruses manifests as an evolutionary advantage on infection and transmissibility, either at monogenic or polygenic levels, via mechanisms such as receptor binding, growth efficiency and antagonizing host immune response [9,10]. It was concluded that the last five influenza pandemics in 1918, 1957, 1968, 1977 and 2009 were caused by reassorted IAVs [11,12]. Consequently, it is of utmost importance to identify the adaptation and the adaptive reassortment of avian IAVs.

Host-adaptation transitions of IAV are governed mainly by hemagglutinin (HA)-mediated receptor binding to human or avian receptors (α2,6- or α2,3-linked sialic acids), neuraminidase (NA)-mediated viral release, and viral RNA polymerase complex-mediated viral replication efficiency [8,13]. The receptor binding specificity of an IAV strain is definite and easily distinguishable. H5 and other serotypes primarily bind to the avian receptor, with the exception of H3, H2 and H1 [14]. However, the multiple polymerase-related gene (*PB2, PB1, PA* and *NP*)-mediated adaptation was much more complicated and difficult to identify. Up to now, it has been well established that the receptor binding of H5N1 is avian type [15]; however, the adaptation of each of the polymerase-related genes and the potentially human-adapted reassortment mediated by these genes with other IAVs remain unknown. IAV undergoes frequent genetic reassortment by exchanging its segment(s) during co-infection. Current evidence indicates that IAV segment reassortment is governed by intrinsic genomic packaging constraints, rather than occurring at random. Each RNA segment contains cis-acting signals that direct a highly ordered ‘7 + 1’ assembly, preserving genomic integrity and particle infectivity [16,17]. These signals impose compatibility requirements to the backbone genome on incoming segments [17,18]. However, the interaction network is not stringent, allowing limited ‘fault-tolerance’ segment combinations, providing the substrate for occasional reassortment events and subsequent adaptive exploration [18]. Moreover, the host adaptation is more important to evaluate the pandemic potential of a possible IAV reassortant. Only the higher human-adapted reassortant is able to dominate the virus population and develop a novel strain, posing pandemic risk. Additionally, different segments possess different reassortment rates. The NS segment displays elevated reassortment rates across both human and avian viruses [19], while the other five internal gene segments are similarly lower, but the reassortment rates differ significantly among different serotypes [19,20]. Besides, the newly formed reasserted progeny particles are also constrained by host factors, such as ANP32A [21], MxA [22] and BTN3A3 [23] via various molecular mechanisms. These findings underscore the interplay of segment compatibility and host biology in driving IAV reassortment.

H5N1 IAVs have been recorded to cause human infections since the late 1990s [24], and later constantly from 2003 to 2014 [25], mainly in East Asia. More pathogenic H5N1 IAVs with clade 2.3.4.4b H5 [26] emerged in wild birds worldwide [27] and adapted to mammals quickly [28]. More worryingly, mammal H5N1 infection has been widely reported in Europe [29], North America [30] and Central and South America countries [31] since 2022, implying a high risk of mammalian adaptation and mammal-to-mammal transmissibility [32], like in bovine herds in the USA [33]. Thus, it is reasonable to worry about a potential pandemic caused by H5N1 IAVs, most likely via reassorting with human IAVs.

Artificial intelligence (AI) approaches have brought significant achievements in virus genotype–phenotype learning, such as the prediction of IAV’s host adaptation [34], the prediction of viral reservoir hosts and arthropod vectors [35], and the prediction of the adaptation shift of SARS-CoV-2 [36,37] or other coronaviruses [38,39], particularly the Natural Language Processing (NLP) embedding of protein sequences leads to deeper learning about the association of protein genotypes and phenotypes, such as protein structure [40] and virus evolution [41]. Considering the biosafety and the ethical issue of the experimental reassortment test between H5N1 and human IAVs, it is urgent to build an AI predictor to assess the human adaptation potential of reassortments between H5N1 and human IAVs. Such AI embedding or prediction should not pose secondary biosafety and ethical issues, such as pretraining an embedding tool based on a large gene sequence dataset, or training a generator of high-risk virus genomes. Several bioinformatical methods have emerged to analyze IAV reassortment by exploiting either evolutionary history and sequence-derived compatibility signals, such as phylogeny-based pipelines of GiRaF and FluReF [42,43] or alignment-free techniques [44]. Moving further toward phenotype-oriented prediction, the random forest classifier of HopPER allowed probabilistic inference of host tropism and, by extension, host-specific segment compatibility [45], though without experimental validation of its predicted reassortment. Thus, more efficient but safe NLP embedders of IAV genes and more intelligent networks seem expected to assess the reassortants with high adaptation potential of current H5N1 IAVs, particularly combining the validation by biological approaches.

The present study provided an intelligent framework of Human Adaptive Influenza virus Reassortment using Attentional Networks based on Genome Embedding (HAIRANGE), to predict the high human adaptation potential of reassortants involving the four RNA polymerase-related genes of *PB2, PB1, PA* and *NP* for the prevalent H5N1 IAVs with human H3N2 IAVs. HAIRANGE integrated a novel genomic context embedder Codon2Vec and a deep learning predictor to assess the high human adaptation potential of IAV reassortants. The non-pretrained Codon2Vec was to embed the viral gene sequence as a contextual matrix, which represents each codon and its coded residue, dependent on its upstream and downstream codons and residues, thus at a fine-grained level to code both RNA and protein information. The ResNet classifier in HAIRANGE was to predict the adaptation and adaptive reassortment of polymerase genes, with both an interpretable ablation and a biological validation of polymerase activity. The framework of intelligent prediction and biological validation provides a timely and interpretable assessment of the high pandemic risk posed by the prevalent H5N1 IAVs.

## RESULTS

### Pandemic risk posed by the steep rise of H5N1 prevalence since 2020

**Figure 1.**
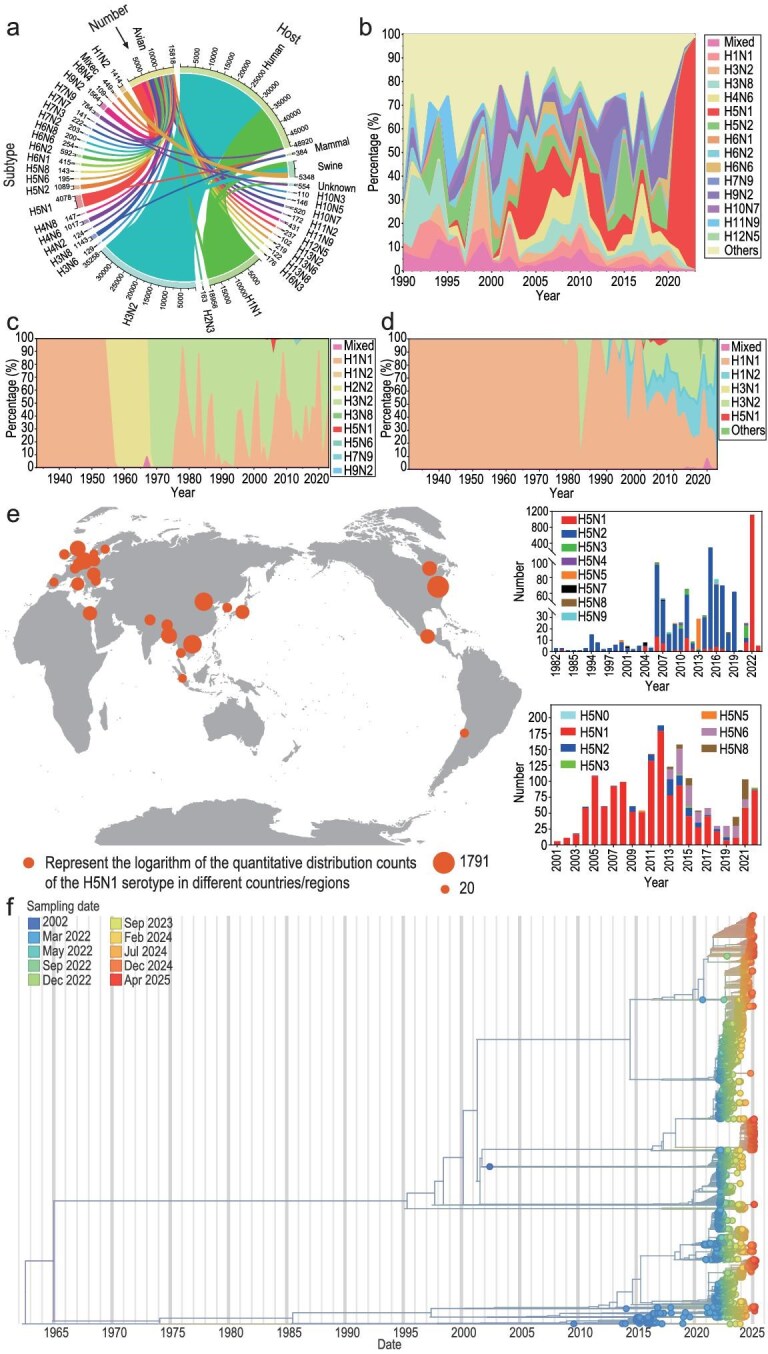
Spatial and temporal distribution of IAVs. A spatial and temporal statistical analysis of all IAVs up to 2023. (a) The serotype inclusion relationships among various hosts; the hosts are primarily classified as humans, birds, pigs, other mammals and unknown. The proportions of different serotypes in avians (b), humans (c) and swine (d) across different years are examined. (e) The quantitative distribution of the H5N1 serotype across different countries, focusing on the top 25 countries, and cumulative numbers of H5N1 cases in North America and Asia over various years. (f) The time-scaled tree created using Nextstrain with the NP segment of H5N1. Review drawing number: GS 京 (2025) 2291号.

To investigate the current prevalence and epidemiological trends of IAVs, a spatial and temporal distribution of collected IAVs was conducted. Initially, the containment relationships and quantitative distributions revealed that the primary IAV serotypes collected from humans and swine were H3N2 and H1N1, while those from avians were primarily H3N8, H4N6, H5N1 and H9N2 (Fig. 1a). Additionally, the proportions of various serotypes collected from avians before 2020 and those from humans and swine exhibited a fluctuating trend (Fig. 1b–d). However, the H5N1 serotype IAVs collected from avians after 2020 increased significantly. While our dataset contains the largest number of sequences from North America, likely reflecting more intensive surveillance there, recent detections now span every populated continent (Fig. 1e), underscoring the worldwide circulation and continued expansion of H5 series serotypes. Based on a time-scaled tree created using Nextstrain with the NP segment of H5N1 in our dataset, H5N1 IAV has undergone several significant genetic differentiations during its spread in recent decades (Fig. 1f). Around 2000, there was a key early differentiation node for H5N1. Subsequently, the period between 2015 and 2020 saw a large number of branches in the H5N1 phylogenetic tree, also representing the main period of clade 2.3.4.4 differentiation. After 2022, H5N1 experienced a peak of ‘explosive’ genetic differentiation, indicating its entry into a rapid evolution phase, and suggesting the intensification of global prevalence and adaptive evolution.

### Framework of HAIRANGE to predict IAV adaptation

**Figure 2.**
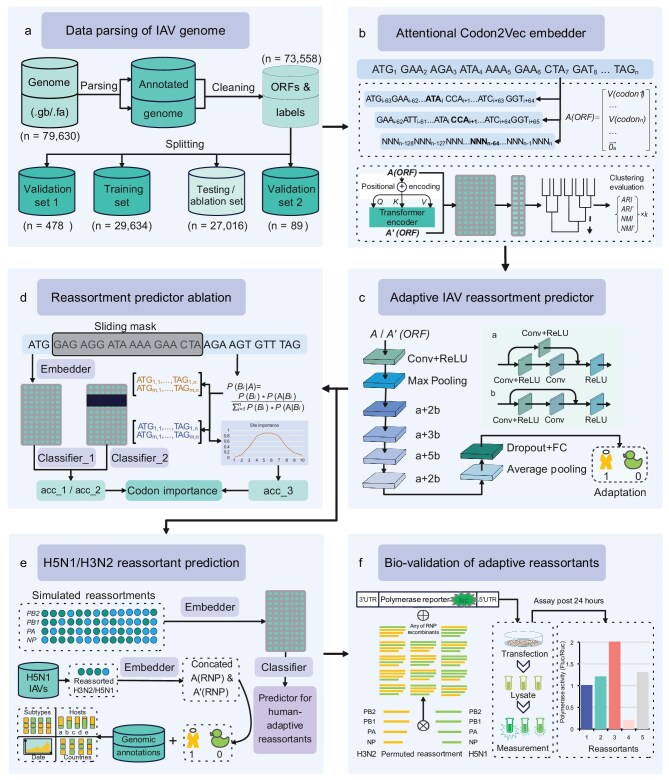
HAIRANGE workflow for genome embedding, adaptive IAV reassortment prediction and polymerase activity validation. The pipeline of HAIRANGE consists of six modules. (a) The data parser of the IAV genome. (b) The attentional codon embedder Codon2Vec. (c) ResNet adaptive reassortment predictor to predict the human adaptation of each of the four IAV genes and reassortment IAVs, based on human H1N1 and avian IAVs. (d) Reassortment prediction ablation network to examine the important codon in the genome context of the four genes, using the embedded data and classification tools and the Bayes model. (e) The adaptive reassortment prediction of H5N1 IAVs with a human H3N2 backbone virus based on the simulated RdRp genomes. Human-adapted H5N1 IAVs were further analyzed by their temporal and spatial distribution. (f) Biological validation of the human-adapted reassortment of H5N1/H3N2 RNA polymerase segments with a luciferase reporter of IAV RNA polymerase.

A framework was designed to intelligently predict and biologically validate the human adaptation potential of each of the individual polymerase-related genes and the reassortants of these genes from avian and human viruses. The HAIRANGE pipeline consisted of six successive modules. Firstly, the four open reading frame (ORF) sequences of the RNA polymerase-related genes of *PB2, PB1, PA* and *NP* were cleaned from the public database and split into training, test and valid datasets (Fig. 2a, Figs S1–S2). Secondly, each ORF sequence was embedded with a genomic context embedder named Codon2Vec, which was decorated with an attention mechanism when utilized to adaptation prediction (Fig. 2b). Codon2Vec was designed to represent an ORF sequence into sequential codons, and then to embed

each codon in the sequence into a codon frequency vector with a dimension of 64, within a window of contextual codons. Thirdly, the ResNet predictor for the adaptation and adaptive IAV reassortment of the four genes was built based on convolutional neural networks (CNNs) and four residual blocks (Fig. 2c). The fourth ablation network module was to verify the reliability and the interpretability of the model (Fig. 2d). The fifth part was to simulate a reassortment between avian and human IAVs, then to predict the human adaptation potential of the simulated reassortants with the trained predictor (Fig. 2e). Finally, the predicted adaptive reassortment of avian H7N9, H9N2 and H5N1 with human H3N2 polymerase-related genes was bio-validated via *in vitro* polymerase assay (Fig. 2f).

### Competitive performance of Codon2Vec in embedding benchmarking

**Figure 3.**
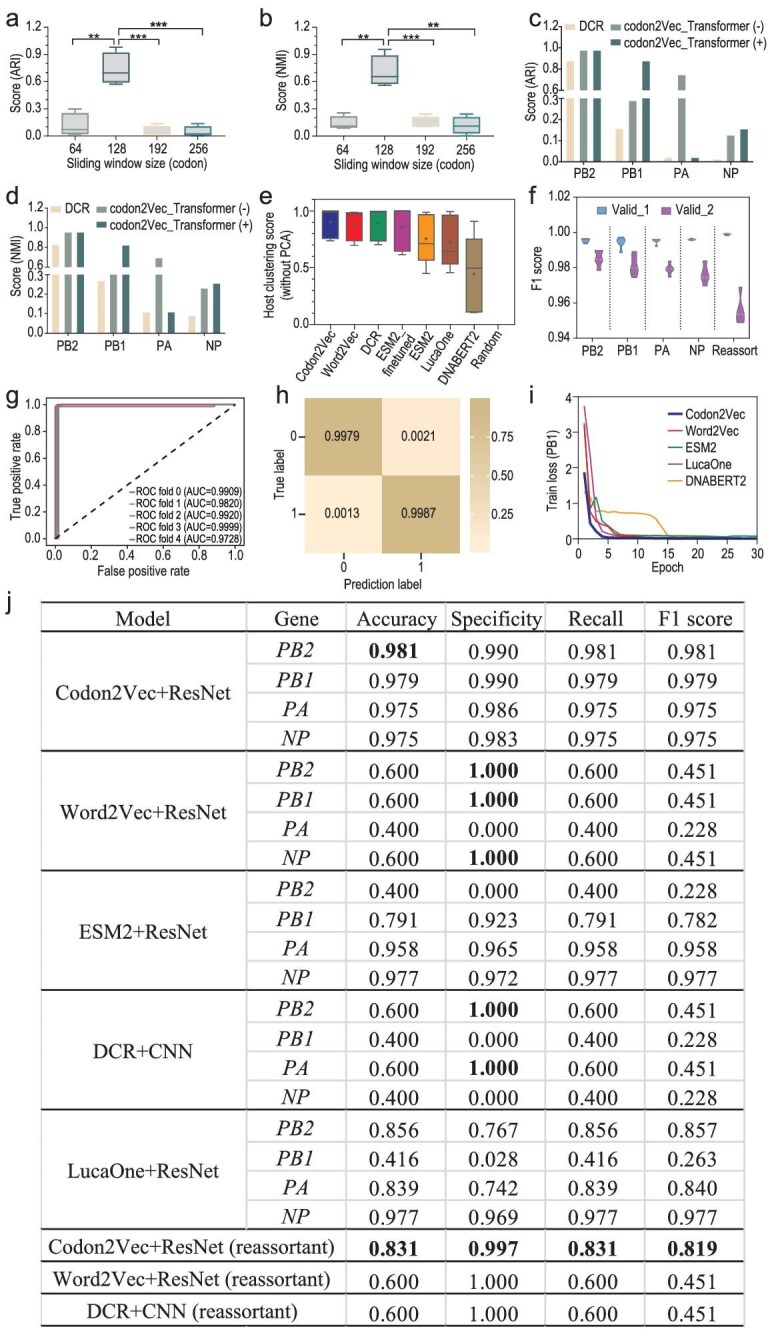
Embedding performance on IAV RdRp genes and prediction performance of HAIRANGE on the human adaptive reassortment of IAVs. The embedding tool of Codon2Vec for contextual codons in the IAV genome was optimized with a sliding window size of 64, 12 192 or 256 codons, respectively, with the adjusted Rand index (ARI) (a) and normalized mutual information (NMI) (b) as scoring indexes. The embedding performance of Codon2Vec with or without attention pretraining was compared to dinucleotide composition representation (DCR) with ARI (c) and NMI (d). The evaluation index results of host label clustering for embedding results of different embedding methods on sampled datasets (e). The prediction performance of ResNet was evaluated with an F1 score (f) on the training set and two validation sets (valid_1 and valid_2) for PB2, PB1, PA, NP and simulated reassortant polymerase-related genes. ROC (g) and confusion matrix (h) of the reassortant adaptation predictor on Validation Set 2 for adaptive reassortment of the four polymerase-related genes. Training loss of the single-segment model using different embedding matrices and ResNet models (i) and predictive metric results of different models (j).

Considering the importance of the codon context in both viral RNA (vRNA) and messenger RNA (mRNA) and its coded protein levels for viral adaptation to host [46,47] and the unavailability of a tool for codon context embedding, the Codon2Vec tool was built to embed both codon and amino acid contexts of viral genes. Firstly, the parameter of a sliding window size of 128 was optimized based on the embedding performance according to two clustering indexes, compared with the sliding window size of 64, 192 or 256, for each (*P* < 0.05 or *P* < 0.01, Fig. 3a and b). The high embedding performance of Codon2Vec was indicated by its overperforming against the previously reported non-pretrained method of dinucleotide composition representation (DCR) [37] for the four genes. What’s more, the embedding performance was improved by the attention mechanism for *PB1* and *NP* [48], according to clustering (Fig. 3c and d). Such embedding improvement by the attention mechanism was also observed for *PB2, PB1* or *NP*, from the UMAP-reduced embedding data (Fig. S3). In particular, there was a marked separation between avian and human genes, on the UMAP-reduced (UMAP1 or UMAP2) embedding data, either without or with attention, compared to the UMAP-reduced DCR embedding (Fig. S3J–L). Full benchmarking was performed to evaluate the embedding performance of the two non-pretrained tools of Codon2Vec and DCR [37], and of the other pretrained language models of Word2Vec [49], ESM2 [50], DNABERT2 [51], LucaOne [52] and random embedding matrices. ESM2 was also fine-tuned with unique protein sequences coded by the polymerase genes for HAIRANGE training. Comprehensive clustering results, which were indicated by five types of clustering index scores, demonstrated competitive performance of Codon2Vec, against other non-pretrained or pretrained embedding tools (Tables S1–S4). It was worth noting that with the IAV gene data, the fine-tuned ESM2 model (ESM2_fine-tuned) outperformed the original ESM2, and was similar to or better than Codon2Vec on certain clustering indexes for some genes. Taken together, the non-pretrained Codon2Vec performed competitively against ESM2_fine-tuned, much better than the others (Fig. 3e, Figs S4–S6).

### HAIRANGE predicts the adaptation and adaptive reassortant of IAV

To evaluate the human adaptation potential for reassortment of the prevalent H5N1 IAVs with human-adaptive IAVs, four single-gene binary predictors for human-adapted *PB2, PB1, PA* and *NP*, whose predicted adaptive host is human, were independently trained based on the codon context embedding with the available human/avian IAVs. A learning rate of 0.03 and a batch size of 1150 were set uniformly for the four predictors after judging the model training performance with different parameters, and the convergent validity of the predictors was manifested as a rapid and stable decline of training loss and high values of accuracy (Fig. S7A–H). The F1 score for each of the four classifiers was >0.97 on the two validation sets (Fig. 3e), and the receiver operating characteristic (ROC) (Fig. 3f, Fig. S7I–L) and confusion matrix (Fig. 3g, Fig. S7M–P) also performed well. The single-gene prediction model could accurately predict the host labels, but it had poor recognition ability for site mutations in the sequence. For example, at position 627 on the PB2 protein, it could accurately predict the K position but could not accurately predict the 627E position (Fig. S8, Table S5).

For the adaptive potential of IAV reassortant prediction, a classifier was trained based on the embedding of four polymerase-related genes of *PB2, PB1, PA* and *NP* in the training set, with the simulated reassortant of human H3N2/H1N1 IAVs as positive samples, and with the simulated reassortant of avian IAVs as negative samples. The reassortant adaptation model was optimized with similar parameters and also performed well (Fig. 3f–h). The reliability of the reassortant adaptation predictor was evaluated on the two validation sets, indicating that the F1 score was higher than 0.95 (Fig. 3e); the prediction of natural reassortment IAVs by the reassortant adaptation classifier also demonstrated high accuracy (Table S6). Single-gene prediction models for each of the four genes based on embeddings of Codon2Vec, Word2Vec, ESM2, DNABERT2 and LucaOne were trained independently by adjusting the parameters of ResNet. These results revealed that the model trained using Codon2Vec performed well in terms of both computational cost and model performance, under the same condition of training epochs (Fig. 3i–j, Figs S9–S12, Table S7). Specifically, the Codon2Vec-based model demonstrated advantages in efficiently processing the data while maintaining high predictive accuracy and so on. Additionally, the training and prediction performance of the reassortant adaptation models trained with simulated reassortants using the Word2Vec and DCR embeddings were both inferior to those of Codon2Vec (Fig. 3j, Fig. S13). Both the Word2Vec and DCR models converged rapidly while training, which may indicate a risk of overfitting. Consequently, their predictive performance on the validation set was poor. Overall, HAIRANGE holds great potential in developing efficient and accurate predictive models for biological sequences.

To evaluate the reliability of these predictors for IAV adaptation, an ablation experiment was performed. The codon importance (CI) was evaluated as the average vector importance (VI) for codons masked in a sliding window fashion (Fig. S14A). A Naïve Bayes model based on avian and human polymerase-related genes was also utilized as a control for codon importance. The codon importance inferred by these two methods was highly consistent (Fig. S14B). UMAP-reduced dimensionality features of the full-length simulated avian and human polymerase-related genes indicated significant segregation for the IAV from the two types of host (Fig. S14C). Additionally, given the intra-gene and inter-gene compatibility and constraints for reassortment [53], the intra- and inter-gene codon pair for these polymerase-related genes were evaluated, respectively, with inter-codon Spearman correlation and co-occurrence or direct coupling analysis (DCA) (Fig. S14D and E). The majority of critical codons identified through ablation experiments conducted via two distinct methodologies exhibited strong concordance with previously documented findings in the literature. Notably, codons of high importance have not been reported in existing studies, underscoring their potential significance for guiding future research endeavors in this field (Fig. S14F–I, Tables S8–S9).

### Adaptive reassortment of avian IAVs with human H3N2 IAV

**Figure 4.**
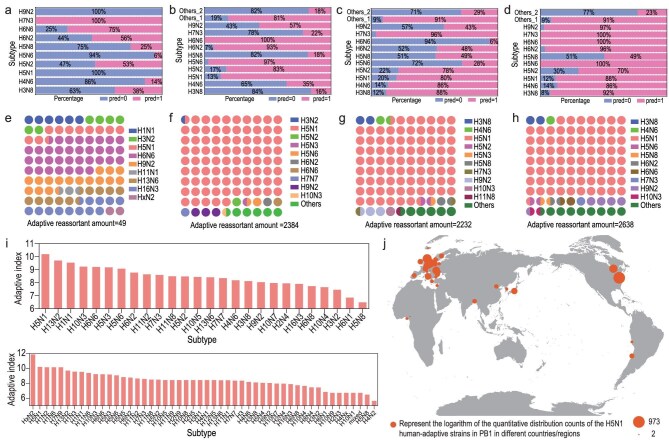
Prediction results and spatiotemporal distribution of avian IAVs after 2020. The adaptation proportions of predictive simulations of reassortment adaptation for all avian IAVs post-2020 in *PB2* (a), *PB1* (b), *PA* (c) and *NP* (d). Here, ‘others_1’ refers to the average value of other serotypes with an adaptation ratio greater than 50%, and ‘others_2’ refers to the average value of other serotypes with an adaptation ratio less than 50%. Serotype analysis on the human-adaptative results from the reassortment adaptation prediction of *PB2* (e), *PB1* (f), *PA* (g) and *NP* (h). (i) The adaptation index calculated based on the prediction results of these four individual segments for the 25 serotypes with the highest quantity and all serotypes. (j) Country distribution of H5N1 human-adaptive strains in *PB1*. Review drawing number: GS 京 (2025) 2291号.

Simulation reassortant adaptation predictions for avian IAVs after 2020 with H3N2 reference IAV were conducted utilizing the trained reassortant adaptation model, and the adaptive results with single-gene simulated reassortment were analyzed. Firstly, the adaptation proportions of various serotypes in the prediction results revealed that the H5N1 and H5N2 serotypes exhibited high human adaptation after *PB1, PA* and *NP* single-gene reassortments, while H5N6 showed high human adaptation after *PB1* and *NP* single-gene reassortment (Fig. 4a–d). Secondly, the proportion of human-adapted serotypes indicated that H5N1 has the largest number of human-adapted viruses after *PB1, PA* and *NP* single-gene reassortments (Fig. 4e–h). The adaptation indexes were calculated based on the quantity distribution and prediction results, and viruses with H5N1 reassorted exhibited the highest risk of adaptation in humans (Fig. 4i). Finally, human-adapted *PB1* single-gene reassortments of H5N1 viruses are revealed most frequently in North American sequence in our dataset (Fig. 4j). This result might be related to the higher sampling density in North America, but it also reflected the increased threat of H5N1 transmission in the region to some extent and indicated the need for continued vigilance and globally coordinated monitoring.

### High polymerase activity of the reassortants of avian IAVs with human H3N2 IAV

**Figure 5.**
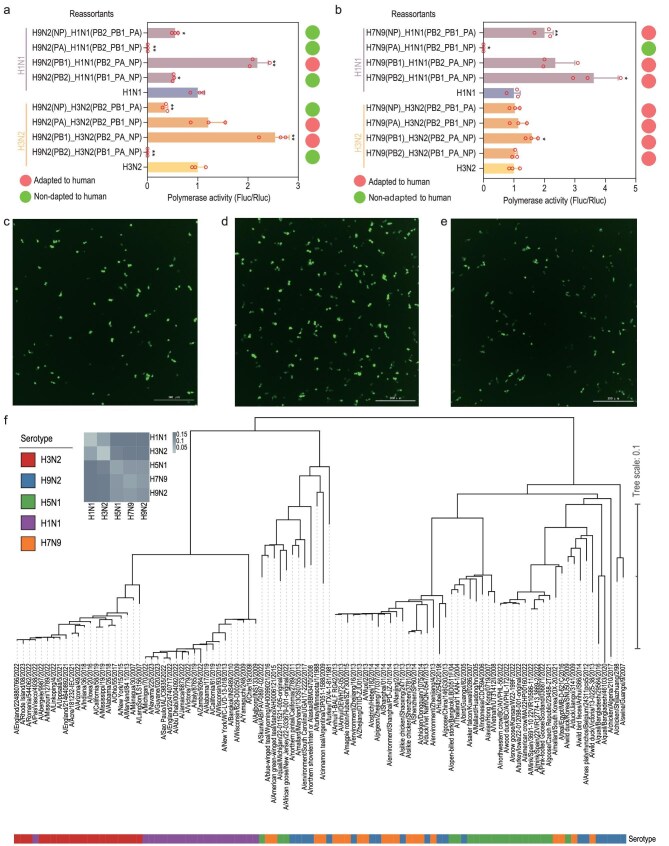
*In vitro* polymerase activity of reassortant RdRps from human and avian IAVs. Single H9N2 (a) or H7N9 (b) polymerase segment was reassorted with H1N1 and H3N2 backbones, respectively, and the activity of reassorted polymerase was assayed by a dual luciferase reporter system with the pHH21 vector connecting with firefly luciferase. The polymerase activity of the H3N2 backbone (c), replaced with PB1 of H9N2 (d) or H7N9 (e) serotype were visualized with the pHH21 vector connecting with green fluorescent protein. Fluorescence intensity indicates the strength of polymerase activity. Phylogenetic analysis was conducted on the PB1 genes, which were randomly selected from H3N2, H1N1, H9N2, H5N1 and H7N9 serotype IAV, 20strains each (f). Subsequently, the average genetic distance between pairs was calculated using Mega software. Data of polymerase activity are represented as the mean ± SD of three independent experiments. Statistical significance was calculated using Student’s *t*-test (*P < 0.05; **P < 0.01; ***P < 0.001).

To biologically validate the reassortant adaptation prediction made by HAIRANGE, polymerase activity determinations were performed for each reassortment of H7N9 and H9N2 with H3N2, respectively. If the polymerase activity after reassortment was significantly higher than or similar to that of the control group, the risk of adaptive reassortment was high; otherwise, the risk was considered low. A dual-luciferase assay was performed using transfection with six plasmids (four polymerase gene expression plasmids, pHH21-Fluc, and pRL-TK) in 293T cells, and both Firefly and *Renilla* luciferase activity were measured. Meanwhile, four polymerase plasmids with pHH21-GFP were also co-transfected to observe the fluorescence intensity. The results showed that the *PB1* and *PA* segments of H9N2, when reassorted with H3N2, yield high polymerase activity, which indicates a risk of human adaptation, especially in *PB1*. All four individual segments of H7N9, when reassorted with H3N2, exhibited a certain degree of adaptation to humans, especially in *PB1* (Fig. 5a–e, Figs S15–S16). These findings are generally consistent with our model’s predictions (Table S10). However, there were discrepancies between the polymerase activity results for the *NP* segment predictions. Additionally, based on homology analysis results from evolutionary trees, H3N2 and H1N1 exhibited high homology, while their homology with H5N1, H7N9 and H9N2 was relatively low (Fig. 5f, Fig. S17). Taking NP as an example, seasonal H1N1 and H3N2 both retain the NP segment that descended from the 1918 virus; they unsurprisingly exhibit higher NP homology to each other than to avian H5N1, H7N9 or H9N2 strains. This observation highlights the limitation of using single-segment similarity alone to infer human adaptation; in contrast, HAIRANGE evaluates nucleotide-context features across all four polymerase segments and can therefore detect reassortment risks that do not involve a pre-existing ‘human-like’ NP.

### Adaptive reassortment of the prevalent H5N1 IAVs with human H3N2 IAV

**Figure 6.**
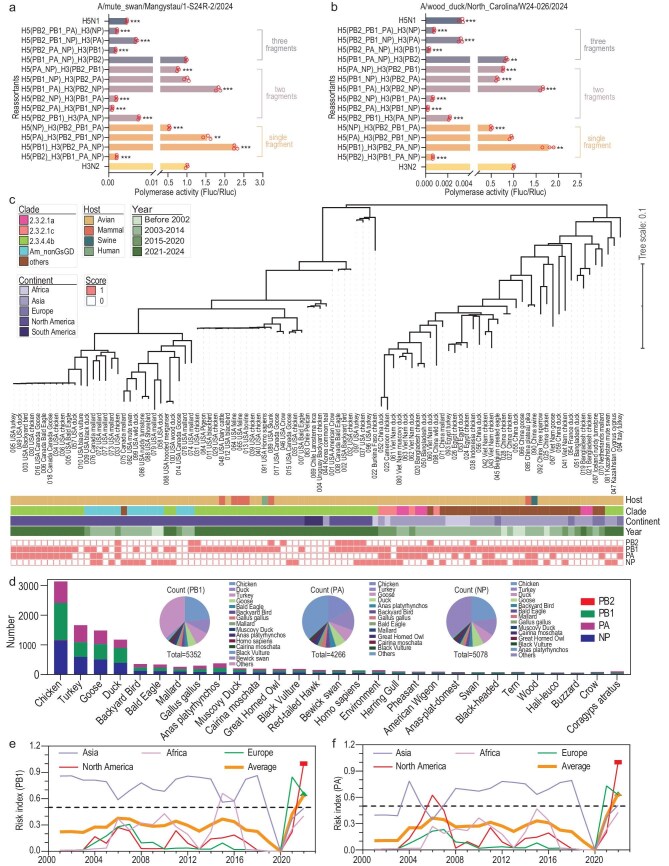
Polymerase activity for H5N1/H3N2 reassortment and epidemiological risk analysis of the prediction results. The polymerase activity assayed by a dual luciferase reporter system with human IAV UTR for the reassortment of a H3N2 backbone with A/mute swan/Mangystau/1-S24R-2/2024 (a) and A/wood duck/North Carolina/W24-026/2024 (b). (c) Phylogenic analysis of concatenated polymerase-related genes from randomly sampled H5N1 IAVs with various adaptations. Data of polymerase activity are represented as the mean ± SD of three independent experiments. Statistical significance was calculated using Student’s *t*-test (*P < 0.05; **P < 0.01; ***P < 0.001). (d) Counting of the polymerase-related genes of H5N1 strains, with adaptive reassortment with a backbone of H3N2 reference IAV, virus host-dependently. Risk indices of PB1 (e) and PA (f) were calculated based on the virus number and adaptive reassortant number for the H5N1 IAVs from different continents in different years.

To validate the prediction reliability of the high human-adapted reassortment between the prevalent H5N1 IAVs and human H3N2 IAV, the *in vitro* polymerase activity of reassortant H5N1/H3N2 polymerase-related genes was examined with an IAV polymerase reporter with human IAV untranslated region (UTR), in human cells. Surprisingly, the single-gene reassortant with H5N1 *PB1* or *PA* gene was much more active, and that of H5N1 *PB2* gene was less active, compared with wild-type H3N2 polymerase complex, for all four H5N1 strains (*P* < 0.0001, Fig. 6a and b, Fig. S18A–D), indicating an accuracy of more than 75% (Fig. S18E). However, the H5N1 *NP* did not significantly affect the polymerase activity, though high human adaptation was also predicted in the context of H5N1 *NP*/H3N2 reassortment. This might be primarily because the multifunctional roles (vRNA binding, nuclear export, immune modulation) of NP required context-dependent synergy with other viral components that single-phenotype assays failed to replicate, and computational predictions might generate subtype-sensitive false-positive adaptive signals. Additionally, the reassortants of H7N9/H3N2 did not indicate significance, compared with wild-type H3N2 polymerase complex (Fig. S18F). On the other hand, the upregulation by H3N2 *PB2*, and the downregulation by H3N2 *PB1* or *PA* were indicated in such an assay (Fig. S18A–D). We repeated such an assay with another polymerase reporter with avian IAV UTR, in human cells. Almost similar consistency between the human adaptation and high polymerase activity in human cells was observed (Fig. S18G–L). The prediction accuracies for the *PB2* gene were both 100%. For the *PB1* gene, the accuracies were 75% and 50%, respectively. For the *PA* gene, the accuracies were 75% and 100%, respectively. For the *NP* gene, the accuracies were 25% and 50%, respectively.

Phylogenic analysis of randomly sampled H5N1 IAVs (Table S11) based on the four polymerase-related genes were manifest in a tree (Fig. 6c). Phylogenetic reconstruction partitioned contemporary H5N1 viruses into two primary lineages: a branch in which clade 2.3.4.4b groups most closely with the American non-Gs/GD (Am_nonGsGD) lineage, and another branch comprising clades 2.3.2.1a, 2.3.2.1c and related sub-clades. The branch of clades 2.3.4.4b and Am_nonGsGD viruses, dominant in North America and South America from 2021 to 2024, indicated a higher human adaptation ratio for *PB2* (5% vs 0%), *PA* (93.3% vs 47.5%), *NP* (90.0% vs 50%), compared with the viruses in the branch of clades 2.3.2.1a, 2.3.2.1c and others. Eighty-five percent of the H5N1 *PB1* genes of clade 2.3.4.4b were human-adapted, though the predominant sub-lineage (left-hand branch of the phylogeny) was a little less human-adapted, compared with the right-hand branch (77.5% vs 70%). The phylogenic analysis of each of the four genes also indicated a dominant adaptation of clade 2.3.4.4b (Fig. S19). Taken together, high human adaptation was predicted for the H5N1 IAVs, particularly in clade 2.3.4.4b, from 2021 to 2024.

To assess the pandemic potential of the prevalent H5N1 IAVs, timely evaluation of viral adaptation and adaptive reassortment is essential. Hundreds of H5N1 *PB1, PA* and *NP* from H5N1 were human-adapted in the H3N2 reassortants; chicken, duck, bald eagle and turkey topped the list of hosts of human-adapted H5N1/H3N2 reassortants with H5N1 *PB1, PA* or *NP* (Fig. 6d). A relatively high risk of H5N1 for each of the four genes fluctuated from 2001 to 2019, with a transient steep rise in Africa in 2015 and 2016 (Fig. 6e and f, Fig. S20). Worryingly, the prevalence of H5N1 viruses in various types of birds since 2022 has increased the average risk index. Based on the analysis of global H5N1 samples in the existing database, North America led this trend in 2022, with risk indices for all four genes rising sharply, indicating a high adaptive reassortment risk of H5N1 IAVs with the prevalent human H3N2 IAVs.

## DISCUSSION

Viral RNA polymerase-related genes have been increasingly recognized to mediate IAV adaptation via regulation of viral replication efficiency [8,13]. Reverse genetics-based explorations have identified the dependence of IAV adaptation on the amino acid residues 627 [54] and 701 [55] in *PB2*, on the residues in the C-terminal of *PB1* [56], on the residues in *PA* [57], and also on the residues in *NP* [55]. On the vRNA level, host-specified codon content has been indicated to be associated with IAV adaptation [58]. However, such an experimental strategy would always be left behind the mutation or reassortment-mediated adaptation of IAVs, and thus was satisfied to generally and intelligently assess adaptive IAV reassortment. The non-pretrained embedder Codon2Vec in our study was designed to represent each codon and it coded amino acid residues dependent on its upstream and downstream codons or residues, and thus could interpretably embed genomic context. Furthermore, Codon2Vec, without being pretrained on large gene sequence data of viruses and other pathogens, posed less worry about secondary biosecurity risk of malicious misuse to generate viruses with gain of function. Embedding benchmarking results recognized the competitive performance of Codon2Vec over several reported language embedders, such as ESM2, DNABERT2 and LucaOne, based on both serotype- and host-based gene clustering. Interestingly, further embedding-based analysis identified the adaptation-associated codon clusters in these genes and the high inter-cluster or inter-gene association, collectively indicating high compatibility and constraints on these polymerase-related genes. We speculated that the high embedding performance of Codon2Vec might be due to the embedder’s highlighting of the indispensable importance of viral codon clusters and their context [46,47], as these might be ignored by other more general embedders. Of course, our benchmarking results did not imply a disadvantage of LucaOne and other language embedders on gene embedding of IAV and other viruses. There might be two reasons to explain their slightly inferior performance: (i) these pretrained embedders were not fine-tuned on IAV polymerase genes; and (ii) the much larger dimension of these models’ embedding results might dilute the genetic importance with higher data dimension and thus did not fit the clustering algorithm. This was validated by fine-tuning ESM2-based IAV genes.

Though current phylogenetic tools and molecular virological methods remain essential and complementary in uncovering viral evolution and host adaptation, HAIRANGE provided an additional layer to explore the importance of codon context. The Codon2Vec-embedded polymerase-related genes predicted accurately viral adaptive hosts with a ResNet architecture. Moreover, HAIRANGE based on Codon2Vec embedding predicted natural reassortant IAVs, with high sensitivity, specificity and accuracy, on two validation datasets. The core function of HAIRANGE was to predict host suitability. It assessed the potential of a given influenza virus gene, or a gene combination created through reassortment, to support efficient viral replication within a target host like humans. This approach differs from methods that directly predict the molecular probability of the reassortment event itself, which are often based on factors like genome packaging signal compatibility or co-infection frequency. Viruses predicted to have high host suitability in a new host (e.g. human) represent a significant phenotypic risk because they possess the core functional competence (efficient replication) necessary to establish infection and potentially transmit within that host population, should a reassortment event introduce them. Thus, HAIRANGE identifies reassortant combinations with a high functional threat potential, prioritizing them for further surveillance and experimental characterization, even if the exact probability of the reassortment event occurring remains unknown. Interestingly, similar ResNeSt architecture based on the embedding of LucaOne, ESM2 or DNABERT2 was much harder to train, requiring more training epochs and time, larger memory and occupation for similar performance to HAIRANGE. Additionally, a simple network like a CNN was not trainable even for Codon2Vec embedding, with a gradient loss at the early training epochs. Thus, more complicated supervised architecture was worth trying to represent the fine granularity in gene embedding of LucaOne and others. The indirect pandemic caused by an adaptive reassortment of H5N1 virus with human IAVs stood out since its definite avian receptor binding reduced the possibility of directly arousing an IAV pandemic. The bovine H5N1 in the USA [33] frequently caused spillover infection in humans and other mammals and even limited inter-ferret transmission [59], and had the chance to co-infect and to meet timely the need to intelligently assess the adaptive reassortment risk of the bovine HPAI H5N1 with human IAVs (including reassortment with H3N2 viruses). Thus, HAIRANGE timely met the need to intelligently assess the reassortment adaptation of the bovine H5N1. The HAIRANGE-predicted adaptive reassortment of avian H5N1 and human H3N2 viruses is worthy of vigilance. A high number of H3N2 backbone-based reassortments with H5N1 *PB1, PA* or *NP* were adaptive. A risk index based on H5N1 prevalence and adaptive reassortment potential demonstrated a marked high pandemic risk up to now. The following phylogenetic analysis traced clade 2.3.4.4b H5N1 strains in the branch with high adaptive reassortment, and the higher pathogenicity and transmissibility of this H5N1 clade [27,30] reconfirmed the higher public health risk.

Most of the currently available phylogeny-based pipelines of GiRaF and others [42–45] were neither competent to assess the reassortment risk of H5N1 polymerase-related genes with human H3N2 virus backbone, nor validated by experimental results. The prediction reliability of HAIRANGE was validated by the *in vitro* polymerase assay. There was a consistency between the HAIRANGE-predicted adaptive reassortment of H5N1 *PB1* or *PA* and the much higher polymerase activity of such a reassortment. On the other hand, the predicted inadaptable reassortment of H5N1 *PB2* was also consistent with its much lower polymerase activity. However, we also acknowledged an important observation regarding the NP segment prediction that while HAIRANGE predicted high human adaptation potential for H5N1 NP in the H3N2 backbone, our *in vitro* polymerase activity assay did not show a significant enhancement compared to the wild-type H3N2 complex. This discrepancy highlights the multi-faceted nature of host adaptation. While polymerase activity is a gold standard for measuring replicative fitness, and our model exhibited good predictive consistency on the *PB2, PB1* and *PA* genes, the *NP* case did highlight the limitations of single-phenotype validation. The function of NP is more complex, involving vRNA binding, nuclear export and even participation in immune modulation; its fitness contribution may be more dependent on specific contexts or synergy with other genes, which may not be fully recapitulated in simple reporter gene systems. At the same time, we also acknowledge the potential for false positives inherent to the model itself. The adaptive signals detected in the *NP* gene may be more subtle or more dependent on specific subtype or lineage backgrounds. Future work could improve the model by incorporating more comprehensive functional experiments (such as multi-cycle growth curves and animal model adaptation assays), or by differentiating more precisely the effects of NP subtypes and key mutations within the training data. In addition, the single key mutation of PB2-627E/K was also assessed via both reporting polymerase assay and adaptation prediction, though our models were not fine-tuned on single-mutation data. PB2-627K was only human-adapted when matched to the other three genes, based on the results of both single-PB2 model prediction and *in vitro* reporting assay. PB2-627K (H5N1) was not adapted when it reassorted with human H3N2 backbone. Considering the biosafety and the ethical issue of the reassortment test between the current H5N1 strain and human IAVs of H3N2 or H1N1, such high-risk reassortment at the virus level was not performed. Therefore, on the one hand, the higher polymerase activity of the reassortant H3N2 polymerase complex with H5N1 *PB1* or *PA* might deteriorate the current H3N2 endemic. On the other hand, a more worrying scenario is that a more adaptive H5N1 IAV with human-adapted *NP* or/and *PB2* might promote the human adaptation of the H5N1 virus, such as human receptor binding. Taken together, the high reassortment potential of the current H5N1 virus is posing a high risk of generating a potential pandemic IAV strain via reassorting with human IAVs.

## CONCLUSION

In summary, there is a human adaptation-specific genomic codon context in RNA polymerase-related genes of IAVs, with which deep learning can predict the human adaptation potential of reassortants between avian and human IAVs. A high human adaptation potential was predicted for reassortants of the current bovine H5N1 virus with human H3N2 IAVs, indicating that such reassortants, if they arise, pose a significant public health risk.

## METHODS

### Genomic context embedder with attentional Codon2Vec

Contextual codons in polymerase-related genes were embedded with Codon2Vec, by which the codon at each position in gene was embedded as a vector of co-occurrence frequency for each of 64 codon types within a sliding window (window size = 128 codons, stride = 1 codon). Each codon was embedded as a codon frequency vector with 64 dimensions, and full gene as a frequency matrix with a shape of (length of each gene) ×64. To satisfy the parameters of subsequent models, the feature matrix was enriched with 0 vectors. The inter-codon association was pretrained via adding attentional layers into the embedding matrix by a transformer encoder [48] named attentional pretrainer, which contains self-attention layers and output of positional encoding layer (Formula 1), where keys, values and queries come from the origin input of the encoder layer.


(1) \begin{eqnarray*}
P{E_{\left( {pos,2i} \right)}} &=& \sin \left(\displaystyle\frac{{pos}}{{{{10000}^{2i/{d_{\rm model}}}}}}\right),\\
P{E_{\left( {pos,2i + 1} \right)}} &=& \cos \left(\displaystyle\frac{{pos}}{{{{10000}^{2i/{d_{\rm model}}}}}}\right).
\end{eqnarray*}

In Formula 1,$\,\,pos$ is position of value, ${d_{\mathrm{model}}}$ is the dimension of embedding, for *i* in the range of ${d_{\mathrm{model}}}$.

### Prediction of adaptive hosts with four single-gene models and a reassortment model

Based on feature matrices of each RNA-dependent RNA polymerase (RdRp) gene from the training set and ResNet architecture, corresponding single-gene host adaptation prediction models were trained. Ablation experiments and Bayesian analysis, among others, verified the models’ reliability. To construct a reassortment model, all avian IAVs were taken as negative samples and 95% human H1N1 plus 5% human H3N2 IAVs were taken as positive samples in the training dataset. The *PB2, PB1, PA* and *NP* embedding matrices were concatenated into one matrix for each sample, which was filled with 0 vectors due to the demand of the model for reassortment model training. The architecture of the reassortment model was similar to the single model, with concatenated embedding data for *PB2, PB1, PA* and *NP* as input. Two validation sets validated the model’s accuracy. To evaluate possible adaptive reassortment between human and avian IAVs, a reassortment simulation was performed to reassort *PB2, PB1, PA* and *NP* genes into one viral genome. Human H3N2 reference strain A/Kansas/14/2017 was selected as a simulated backbone virus, and human feature matrices with one, two or three genes were replaced with those in avians randomly, with 14 situations for each virus, then all simulated genomes based on the human H3N2 reference strain were obtained. The trained reassortment model was utilized to predict host adaptation of simulated avian H5N1 IAVs, with similar embedding data concatenation strategy.

### Adaptation index

The adaptation index was defined as the risk of post-adaptation reassortment for serotype *i* after simulating reassortment among four single genes, with adjustments made for bias in number of serotypes. To evaluate the adaptation index for each serotype, an adaptation index algorithm that balances the initial quantity of virus strain was developed with its predicted human-adapted number, which was defined by following Formula 2:


(2) \begin{eqnarray*}
{\mathrm{adaptive\,\,\textit{index}\,\,}}\left( {\mathrm{i}} \right){\mathrm{\,\,}} = {{\mathrm{e}}^{{{{{{\mathrm{B}}_{\mathrm{i}}}}} \!\, /\, \!{{\mathop \sum \nolimits_{{\mathrm{i}} = 1}^{\mathrm{n}} {{\mathrm{B}}_{\mathrm{i}}}}}}}} + \mathop \sum \limits_{{\mathrm{j}} = 1}^4 {{\mathrm{e}}^{{{{{{\mathrm{A}}_{{\mathrm{ij}}}}}} \!\,/\, \!{{\mathrm{B}}_{\mathrm{i}}}}}}.
\end{eqnarray*}

In Formula 2, *i* is the serotype, *j* is the segment, *n* is the total number of serotypes,${\mathrm{\,\,}}{{\mathrm{A}}_{{\mathrm{ij}}}}$ is the adaptive number of viral strains for segment *j* and serotype *i*, and B_i_ is the total number of viral strains for serotype *i*.

## Supplementary Material

nwaf396_Supplemental_Files

## Data Availability

All data for this study are available on the website https://zenodo.org/records/12747426. Codes have been deposited on GitHub (https://github.com/Jamalijama/HAIRANGE). Any information about the methodology and results of this work is available upon request from the lead contact (Jing Li, lj-pbs@163.com). However, the ESM2_fine-tuned model is only available upon a request with a formal commitment to no malicious misuse.
