# Supplementary material for: Deep learning predicts potential reassortments of avian H5N1 with human influenza viruses
Source: Natl Sci Rev. 2025 Sep 17;12(12):nwaf396. doi: 10.1093/nsr/nwaf396 (PMC12707066; doi:10.1093/nsr/nwaf396)
Supplement: nwaf396_Supplemental_Files [file nwaf396_supplemental_files.zip › Supplementary Figure Legends.docx]

**Supplementary Figure Legends:**

**Supplementary Figure 1. Distribution of all IAV sequences before sampling.** The distribution of IAV by country **(A)**, serotype **(B)**, host **(C)**, and year **(D)** as of 2023.

**Supplementary Figure 2. The overall data distribution of human influenza viruses before 2020 was obtained by random sampling based on serotype, year, and other relevant information.** The data was divided into two parts before 2020 and after 2020. The data before 2020 was divided into the model training set and the validation set, and the data after 2020 was the model testing set. To reduce the impact of uneven data distribution on model training, human influenza viruses were randomly sampled **(A)** before 2020 according to relevant information such as countries and serotypes and kept the ratio of human and avian host source sequences 1:1. The data before 2020 were randomly sampled and distributed by country **(B)**, serotype **(C)**, and year **(D)**

**Supplementary Figure 3. UMAP analysis of the embedded codons between human and avian IAVs.** The human and avian IAV RdRp genes were visualized using UMAP, which projected the matrices into a low-dimensional (UMAP1 and UMAP2) dataset and then were plotted with pair plots. The pair plots showed dimensionality reduction results from Codon2Vec without the utilization of the attention mechanism **(A, D, G, J)**, Codon2Vec with the utilization of the attention mechanism **(B, E, H, K)**, and the utilization of DCR **(C, F, I, L)**, respectively for *PB2* **(A, B, C)**, *PB1* **(D, E, F)**, *PA* **(G, H, I)**, and *NP* **(J, K, L)**

**Supplementary Figure 4. Comparison of embedding performance between different methods of 2000 sampled IAVs with host label.** RdRp genes were sampled and embedded using Codon2Vec, Word2Vec, DCR, ESM2, ESM2_finetuned, LucaOne, and DNABERT2, and then PCA analysis was utilized to reduce the dimensionality into two dimensions, visualized with scatter plots. The first dimension (PCA1) of all the samples was utilized as a vector for correlation analysis. The vectors of the two methods were also visualized. PCA reduced embedding data by Codon2Vec **(A)**, Word2Vec **(B)**, DCR **(C)**, ESM2 **(D),** ESM2_finetuned **(E),** LucaOne **(F),** or DNABERT2 **(G),** was plotted, respectively forh the segment of *PB2*, *PB1*, *PA*, and *NP*.

**Supplementary Figure 5. Comparison of embedding performance between different methods of 330 sampled IAVs with serotype labels.** RdRp genes were sampled and embedded using Codon2Vec, Word2Vec, DCR, ESM2, ESM2_finetuned, LucaOne, and DNABERT2, and then PCA analysis was utilized to reduce the dimensionality into two dimensions, visualized with scatter plots. The first dimension (PCA1) of all the samples was utilized as a vector for correlation analysis. The vectors of the two methods were also visualized. PCA reduced embedding data Codon2Vec **(A)**, Word2Vec **(B)**, DCR **(C)**, ESM2 **(D),** ESM2_finetuned **(E),** LucaOne **(F),** or DNABERT2 **(G),** was plotted, respectively for the segment of *PB2*, *PB1*, *PA*, and *NP*.

**Supplementary Figure 6. The evaluation index results of clustering for embedding results of different embedding methods on sampled datasets.** Embedding of the sampled dataset with host labels (**A**) and serotype labels (**B**), were clustered without dimensionality reduction with PCA, or with PCA reduction (**C** and **D**).

**Supplementary Figure 7. Training and prediction performance of a ResNet classifier for human adaptive IAVs.** The training loss on the train data set **(A-D)** and valid accuracy on the test set **(E-H)** of the *PB2*, *PB1*, *PA*, and *NP* ResNet classifier for 4 RdRp genes using different parameters were shown. The ROC curve **(I-L)** and confusion matrix **(M-P)** with independent validation set 2 were validated using the trained predictor of *PB2*, *PB1*, *PA*, and *NP*. Additionally, five independent repeated tests were sampled for each segment while validating the model through the ROC curve, with the segment of *PB2* **(A, E, I, M)**, *PB1* **(B, F, J, N)**, *PA* **(C, G, K, O)**, or *NP* **(D, H, L, P)**

**Supplementary Figure 8. The sequence prediction results for the 627EK protein site on the PB2 gene.** Host adaptation predictions were made for the wild-type avian-human IAV sequence, and confusion matrices for the predictions under the avian-human label **(A)** and the KE label **(B)** were plotted.

**Supplementary Figure 9. The training loss and validation accuracy performance of models using different embedding methods.** Data embedded by Codon2Vec, Word2Vec, ESM2, LucaOne, and DNABERT2 were trained using ResNet with the same parameter set of learning rate (1150) and epoch (30). .The training losses **(A-D),** and validation accuracies **(E-H)** were presented here, specifically for the training results on the *PB2* **(A, E)**, *PB1* **(B, F)**, *PA* **(C, G)**, or *NP* **(D, H)**.

**Supplementary Figure 10. The confusion matrix performance of models using different embedding methods.** Data embedded by Codon2Vec, Word2Vec, ESM2, LucaOne, and DNABERT2 were trained using ResNet with the same parameter set of learning rate (1150) and epoch (30). The training confusion matrices of Codon2Vec **(A-D)**, Word2Vec **(E-H)**, ESM2 **(I-L)**, LucaOne **(M-P)**, and DNABERT2 **(Q-T)** were presented here, specifically for the training results on the *PB2* **(A, E, I, M, Q)**, *PB1* **(B, F, J, N, R)**, *PA* **(C, G, K, O, S)**, or *NP* **(D, H, L, P, T)**.

**Supplementary Figure 11. The ROC performance of models using different embedding methods.** Data embedded by Codon2Vec, Word2Vec, ESM2, DNABERT2, and LucaOne were trained using ResNet with the same parameter set of learning rate (1150) and epoch (30). The ROC curves and AUC of Codon2Vec **(A-D)**, Word2Vec **(E-H)**, ESM2 **(I-L)**, LucaOne **(M-P)**, and DNABERT2 **(Q-T)** were presented here, specifically for the training results on the *PB2* **(A, E, I, M, Q)**, *PB1* **(B, F, J, N, R)**, *PA* **(C, G, K, O, S)**, or *NP* **(D, H, L, P, T)**.

**Supplementary Figure 12. The PR performance of models using different embedding methods.** Data embedded by Codon2Vec, Word2Vec, ESM2, DNABERT2, and LucaOne were trained using ResNet with the same parameter set of learning rate (1150) and epoch (30). The Precision and Recall curves of Codon2Vec **(A-D)**, Word2Vec **(E-H)**, ESM2 **(I-L)**, LucaOne **(M-P)**, and DNABERT2 **(Q-T)** were presented here, specifically for the training results on the *PB2* **(A, E, I, M, Q)**, *PB1* **(B, F, J, N, R)**, *PA* **(C, G, K, O, S)**, or *NP* **(D, H, L, P, T)**.

**Supplementary Figure 13. The training process for reconfigured benchmarking models.** Utilizing the ResNet framework to train the reconfigured prediction models for Codon2Vec and Word2Vec, and employing CNN to train the DCR reconfigured prediction model. (**A, B**) represents the confusion matrices during the training process, while (**C, D**) depicts the ROC curves. Specifically, (**A, C**) pertain to the Codon2Vec reassortment model, and (**B, D**) to the Word2Vec model.

**Supplementary Figure 14. Ablation of human adaptation, adaptation-associated codon contexts of polymerase-related genes, and the codon importance results.** The ablation of adaptation-important contextual codons was performed with a ResNet classifier for human and avian IAV genes based on embedded genes post sliding masking window, with a classification accuracy reduction as codon importance index (CI) **(A)**. **(B)** The codon importance similarities of the above two methods of four segments. **(C)** The embedded matrices with concatenated codon contexts of human and avian were respectively reduced into one UMAP component, with peak distance between avian and human polymerase-related genes distribution indicated. Co-evolution of all codons within and between polymerase-related genes was performed with Direct coupling analysis (DCA) **(D)** and Spearman co-occurrence method **(E)**. After the ResNet classifier processed the RdRp gene sequences, the importance of each codon in *PB2* **(F)**, *PB1* **(G)**, *PA* **(H)**, and *NP* **(I)** genes was determined through the sliding window ablation and Bayes model.

**Supplementary Figure 15.** **Fluorescent observation of H3N2 and H9N2 IAV polymerase activity.** The polymerase activity of the reassorted H3N2 backbone with H9N2 was visualized with the pHH21 vector connecting with a green fluorescent protein (**A-P**). The stronger the fluorescence intensity, the higher the reassorted polymerase activity.

**Supplementary Figure 16.** **Fluorescent observation of H3N2 and H7N9 IAV polymerase activity.** The polymerase activity of the reassorted H3N2 backbone with H7N9 was visualized with the pHH21 vector connecting with a green fluorescent protein (**A-P**). The stronger the fluorescence intensity, the higher the reassorted polymerase activity.

**Supplementary Figure 17. Phylogenetic analysis of four genes from different serotypes of IAV.** Phylogenetic analysis was conducted on the *PB2* **(A)***, PB1* **(B)***, PA* **(C),** and *NP* **(D)**, which were randomly selected from H3N2, H1N1, H9N2, H5N1, and H7N9 serotype IAV for 20 strains each. Subsequently, the average genetic distance between pairs was calculated using Mega software.

**Supplementary Figure 18. Polymerase activity validation of human-adapted H5N1/H3N2 reassortants.** The polymerase activity was assayed by a dual luciferase reporter system with human IAV UTR for the reassortment of a H3N2 backbone with A/Texas/37/2024 (H5N1) **(A)**, A/hooded merganser/South Carolina/W24-028/2024 (H5N1) **(B)**, A/mute swan/Mangystau/1-S24R-2/2024 **(C)** and A/wood duck/North Carolina/W24-026/2024 **(D)**. **(E)** Prediction accuracy of simulated adaptive reassortants with the polymerase activity by the reporter with human IAV UTR as the true label. (**F)** The polymerase activity by dual luciferase reporter system with human IAV UTR for H7N9 / H3N2 reassortment as negative control. **(G-J)** The polymerase activity was assayed with the dual luciferase reporter system with avian IAV UTR, respectively for the four H5N1 strains. **(K)** Prediction accuracy of simulated adaptive reassortants with the polymerase activity by the reporter with avian IAV UTR as the true label. **(L)** The polymerase activity by the reporter with avian IAV UTR for H7N9 / H3N2 reassortment as negative control. Data of polymerase activity are represented as the mean + SD of three independent experiments. Statistical significance was calculated using Student’s t-test (*p < 0.05; **p < 0.01; ***p < 0.001).

**Supplementary Figure 19. Phylogenetic analysis of randomly sampled IAVs with various adaptations.** Diverse host influenza viruses were randomly sampled, including those belonging to distinct clades, distinct years, and distinct continents. Then phylogenetic analysis were conducted on *PB2* **(A)**, *PB1* **(B),** *PA* **(C),** and *NP* **(D)**.

**Supplementary Figure 20. Risk index of H5N1 over the years.** The host adaptation of all H5N1 viruses was predicted utilizing the trained reassortment prediction model, and based on the prediction results, the risk index is calculated for the four segments: *PB2* **(A)**, *PB1* **(B)**, *PA* **(C)**, and *NP* **(D)**
