# Supplementary material for: Deep learning predicts potential reassortments of avian H5N1 with human influenza viruses
Source: Natl Sci Rev. 2025 Sep 17;12(12):nwaf396. doi: 10.1093/nsr/nwaf396 (PMC12707066; doi:10.1093/nsr/nwaf396)
Supplement: nwaf396_Supplemental_Files [file nwaf396_supplemental_files.zip › Supplementary methods.docx]

**Supplementary methods**

**Data preparation of IAV genome sequences and annotations**

Full IAV genome sequences (number = 79, 630) were downloaded from the websites of NCBI (https://www.ncbi.nlm.nih.gov/nuccore), GISAID (https://www.gisaid.org/), and BVBRC (https://www.bv-brc.org/). A data cleaning was performed to filter the IAV sequence samples without full length, and those with low-quantity. A total of 73, 558 IAV Open Reading Frames (ORFs) were obtained post-deduplication based on all four RNA polymerase-related genes of PB2, PB1, PA, and NP, which were aligned with Mafft (v7.520) ^[1]^. The four polymerase-related ORFs before 2020 were utilized for model training after 1:1 sampling (number = 23, 707) and those post-2020 (inclusive 2020, number = 27, 016) were utilized for ablation experiments and testing. The IAV strains obtained by searching from NCBI with the keyword "reassortant" were utilized as validation set 1 (valid_1, number = 478), and the IAV strains whose strain name contains the ‘reassortant’ was utilized as validation set 2 (valid_2, number = 89). Human IAV were all full-length IAV genomes whose host field is “Human”, including all pandemic strains (1918 H1N1, 1957 H2N2, 1968 H3N2, 2009 H1N1) and their descendant seasonal strains. Lab-adapted strains were explicitly excluded. Additionally, IAVs of the H7N9 serotype (number = 2, 367) and the H9N2 serotype (number = 1, 622) were selected separately to verify the model from the perspective of biological experiments. IAVs of the H5N1 serotype (number = 6, 199) were selected separately for adaptive reassortment prediction.

**Data processing and sequence embedding**

Full IAV genome sequences (with all eight genes comple in coding sequence) were downloaded and selected for embedding and adaptation prediction. The Open Reading Frames (ORFs) for *PB2, PB1, PA* or *NP* were utilized for embedding with Codon2vec, Word2vec, DNABERT2, or DCR at nucleotide level, while the ORF-coded protein sequences were utilized for embedding with ESM2 and its finetuned model, in protein level. The PB1-F2 and PA-X sequences were not independently utilized in the present study. Alignment of ORF sequences were performed with Mafft (v7.520) before embedding, with same sequence length of 760, 758, 717 and 499, respectively for the four genes. The Codon2vec embedder was designed to calculate the richness of each of the 64 types of codons with context window of upper stream 192 (64 codon length) nucleotides downstream192 nucleotides for each codon, serial sliding windows were taken to to count all codon frequency as the embedding result of the codon in the middle of the sequence window. The Codon2vec was not pretrained and available for embedding any ORF sequence, with matched dimension number of 64-dimensioned codon vectors, with ORF sequence length. The word2vec for codon embedding was pretrained with the word2vec architecture and with IAV polymerase ORF as training data. The ESM2 was finetuned with deduplicated four polymerase-related viral protein sequences of PB2 (sampled 6159 from 11576), PB1(sampled 6159 from 10393), PA (sampled 6159 from 11331) and NP (all 6159). based on the esm2_t33_650M_UR50D model. The pretrained ESM2 (esm2_t33_650M_UR50D), DNABERT2 (DNABERT-2-117M), LucaOne (llm_version:lucaone, llm_step:36000000) was also directly utilized for embedding. A matrix with a dimension of seqlen (sequence length) x 64, seqlen x 1280, or seqlen x 2560 was obtained for the embedding by Codon2vec/Word2vec, ESM2/ESM2_finetuned, or DNABERT2/LucaOne. A uniform vector of 1536 dimension was obtained by DCR embedding for any ORF.

**Acquisition of naturally reassortant validation sets**

To obtain naturally reassorted influenza virus sequences that exist in nature, we conducted a search on NCBI based on the keywords "influenza" and "reassortant", and processed the retrieved data using the same methods as those applied to the complete dataset. Ultimately, we obtained 1,168 genomes for all four segments.

**Unsupervised machine learning**

To visualize feature distribution and the effect of attentional pre-training, dimensionality reduction was performed using Uniform Manifold Approximation and Projection (UMAP)^[2]^ for features of the four polymerase-related genes with or without attention pre-training. Before the dimensionality reduction, the feature matrixes were reshaped and then reduction into two main components was performed with UMAP using umap.UMAP. Two main components (UMAP1 and UMAP2) for all three methods were plotted with the host label for each segment using the Python Seaborn package. Another unsupervised machine learning (ML) approach, agglomerative clustering^[3]^, was utilized for cluster analysis of features of the four genes with or without attentional pretraining after dimensionality reduction. According to Euclidean distance (formula I), two dimension reduction features were clustered into two groups using sklearn.cluster.AgglomerativeClustering (n_cluster=2, linkage = “complete”). Two evaluation indicators, Adjusted Rand Index (ARI) and Normalized Mutual Information (NMI) were utilized to evaluate the clustering results. Before the clustering, the dataset was randomly sampled three times.

$\left\| a-b \right\|_{2}=\sqrt{\sum{(a-b)}^{2}}$, (I)

**The analysis of PCA**

To visualize data distribution and clustering, Principal Component Analysis (PCA)^[4]^ was used to reduce the dimensionality of the full-dimensional features of four RNA-dependent RNA polymerase (RdRp) genes. PCA was performed using sklearn.decomposition.PCA. The two primary components (PCA1 and PCA2) utilized the Python Seaborn package to visually represent the host label associated with each data point.

**Architecture for predicting adaptive viral genes or viral reassortants**

The coding DNA sequence (*ORF*) of *PB2, PB1, PA* or *NP* was utilized for embedding with Codon2vec, Word2vec, DNABERT2, or DCR, the coded protein sequences of each of the four genes was utilized for embedding with ESM2 or the ESM2-finetuned embedder, ESM2_finetuned. To predict the adaptation of the IAV gene, a ResNet classifier with 34 layers and about 21.8 million parameters was built based on codon feature matrixes and host labels (0: “Avian” or 1: “Human”), with Convolutional Neural Networks (CNN) and four residual blocks. To satisfy the parameters demand of the training, the PB2 features matrixes was padded with zeros, with dimension changed from (760, 64) to (768, 64). Then the feature matrixes were reshaped into (3, 128, 128) as an input of the classifier. The ResNet predictor was constructed with a CNN layer and with 64 out channels, then a Maximum Pooling layer with 64 out channels, and then residual block calculation for 3, 4, 6, 3 layers, with 64, 128, 256, and 512 out channels sequentially. In all the above calculations, BatchNorm normalization was followed for each convolution calculation, and ReLU activation (formula II) was followed for most of the normalization. An Average Pooling layer and a Dropout layer with a dropout ratio of 0.2 were utilized after residual block calculation, and then a Full-Connected layer was utilized to convert 512 dimensions to 2 dimensions for output prediction, and the Softmax (formula III) was utilized for prediction probability. The model for *PB1, PA* or *NP* was built with similar ResNet architecture, with features matrixes of each sequence changed to (768, 64) for *PB1* and *PA*, to 512 for *NP*. The reassortment classifier was trained with similar ResNest architecture to single gene model, with concatenated embedding data as input ( with dimension of (768 + 768 + 768 + 512, 64) for each reassortant sample). A reassortment classifier was not trained for the emedding data by ESM2, ESM2_finetuned or LucaOne, due to the incapability of our computing device.

The data for training was split into five training/validation sets before constructing the model for 5-fold cross-validation. To validate the ResNet performance, confusion matrixes, accuracy curves, F1 score, and Receiver Operating Characteristic (ROC)^[5]^ curves with Area Under Curve(AUC) using two validation sets were drawn.

$ReLU(x) = max(0, x)$, (II)

$Softmax(x_{i}) = \frac{e^{x_{i}}}{\sum_{i}^{c} e^{x_{i}}}$, (III).

A ResNet architecture was utilized to predict the human adaptation of each of the reassortant IAV polymerase-related genes. The ResNet classifier was built based on codon feature matrixes and host labels (0: “Avian” or 1: “Human”) of IAV sequences, with Convolutional Neural Networks (CNN) and four residual blocks. The data for training was split into five training/validation sets before constructing the model for 5-fold cross-validation. To validate the ResNet performance, confusion matrixes, accuracy curves, F1 score, and ROC curves with AUC using two validation sets were drawn.

**Training configuration**

Device Name: inspur-NF5468Mб

Memory: 503.5 GiB

Processor: Intel® Xeon® Platinum 8358 CPU @ 2.60GHz × 128

OS Name: Ubuntu 20.04.6 LTS

GPU: NVIDIA A800 80GB PCIe

NVIDIA-SMI Driver Version: 535.183.01

CUDA Version: 12.2

**Benchmarking with Word2Vec, DCR, ESM2, ESM2_finetuned, DNABERT2 and LucaOne**

To benchmark the embedding effectiveness with other embedding methods, RdRp genes were sampled 1000 for each host label (human and avian) and embedded using Codon2Vec, Word2Vec^[6]^, DCR^[7]^, ESM2^[8,9]^, DNABERT2^[10,11]^, LucaOne^[12]^, and random embedding matrices. All the embeddings were conducted using the trained embedding model. The gensim.models.Word2Vec Python package was utilized to calculate the Word2Vec sequence compositional features. By setting the parameter ‘vector_size’ to 64, the vector of each codon was calculated separately, then the vectors of codons were formed into matrices according to the sequences. ESM2, ESM2_finetuned, DNABERT2, and LucaOne all utilized the embedding code provided in the README file of the Lucaone, where ESM2 and LucaOne used protein sequences as input, and DNABERT2 used nucleotide sequences as input. The ESM2 was finetuned based on the scripts_ems2_650M with the four viral protein sequence of PB2, PB1, PA and NP, with training_args (num_train_epochs=40, learning_rate=5e-5). All 6159 unique NP sequences and the same number of each of the other three viral protein sequences from the dataset of IAV NP sequences before 2020 were utilized for ESM2 finetuning. The finetuning details were uploaded on Github. The finetuned model, ESM2_finetuned was evaluated with other models for its performance on unsupervised clustering and supervised classification. LucaOne embedding used the LucaOne model whose checkpoint was 3600000, while DNABERT2 embedding used the DNABERT-2-117M model, and then extracted embeddings from the pt file. A dataset with 2000 samples was re-set for embedding benchmarking of influenza genes with 11 types of serotype labels, via a statistical of samples for each serotype. Then Principal Component Analysis (PCA)^[4]^ analysis was utilized to reduce the dimensionality into two dimensions. Sklearn.decomposition.PCA package was used for dimensional reduction of all the embedding features, and PCA1 and PCA2 of each sequence with host labels and serotype labels were standardized and visualized. Additionally, clustering was performed using MiniBatchKMeans, and the clustering results were evaluated using five clustering evaluation metrics: Silhouette Coefficient (SC), Calinski-Harabasz Index (CH), Davies-Bouldin Index (DB), Adjusted Rand Index (ARI), and Normalized Mutual Information (NMI). At the same time, clustering was performed without PCA dimensionality reduction, using the above indicators to compare the representation effects of different representation methods under the serotype labels. To benchmark Word2vec and other models on sample clustering comporehensively, a integrated clustering score was designed based on all the five types of clustering index. A median value of each of the five clustering index values for PB2, PB1, PA and NP was firstly calculated and then normalized for all benchmarked models. All the five normalized clusteing value were taken to integratedly evaluate the relative embedding performance under host or serotype label.

To benchmark the model performance on classification with other embedding methods, the existing ResNet model, performed zero-padding and adjusted relevant model parameters according to the embedding dimensions of Codon2Vec, Word2Vec, ESM2, DNABERT2, and LucaOne. A randomly sampled dataset with 5000 avian viruses and 5000 human viruses was utilized to supervise learning benchmarking for these embedders. Avian/human adaptation of PB2, PB1, PA, and NP genes were predicted via a ResNet model based on each type of these embeddings, respectively for comparing the single-gene prediction models. Subsequently, adaptive prediction models using all five embedders were trained with the same parameter set of learning rate (2e-3), batch size (1150) and epoch (30), for four single polymerase segments respectively. When comparing the reassortment prediction models, due to the excessively high dimensionality of ESM2, DNABERT2, and LucaOne, the Codon2Ve candWord2Vec embeddings and their respective ResNet frameworks with combined four genes for simulating reassortment data were used to train the reassortment prediction models. Here, the model training time, memory consumption, loss during training, and so on were compared. Then, utilizing all the trained models to make predictions on validation set 1, the prediction accuracy, precision, recall, F1 score, and so on were compared to evaluate the model performance.

**Ablation networks.**

Ablation experiments were performed to evaluate the biological interpretation of a classifier for IAV adaptation with a ResNet network and codon feature matrixes post-sliding masking window method. Window size = 80 vectors and stride = 1 vector were utilized to mask the corresponding vectors in the feature matrixes of four segments in the post-2020 set, predict the masked matrixes using the trained single models, and record the accuracy reductions calculated from the accuracies of origin prediction and current masking sliding window prediction. According to the sliding masking window size, the accuracies calculated by the 80 masked matrixes including each vector were averaged to obtain the importance of each vector, and then the importance of the 128 vectors including each codon was averaged to obtain the importance of each codon. The compositional distribution difference of codons at each of all codon sites in each of the four genes between avian and human IAVs was also evaluated with a Naïve Bayes model^[6]^. The importance value was calculated based on the significance of each codon between the two types of IAVs. The prediction accuracy reduction of the predictor was taken as codon importance score, and the control importance score was inferred with a Naïve Bayes model based on avian and human IAV genes. The similarity between those two results was explored based on Euclidean distance and Mean Absolute Error distance (Formula I and Formula IV), and the similarity was calculated by using Formula V.

$MAE =\frac{1}{n} \sum_{i=1}^{n} \left| x_{i} - y_{i} \right|$, (IV)

$similarity (a, b) = \frac{MAX (len(a), len(b)) - DISTANCE (a,b)}{MAX (len(a), len(b))}$, (V)

**Co-occurrence and direct coupling analysis**

Co-occurrence was evaluated as the Spearman correlation^[13]^ of each pair of codon vectors with the Scipy package (scipy.stats.spearman). Direct Coupling Analysis (DCA) was calculated with the pydca package (<https://pypi.org/project/pydca/>)^[14-16]^ as the mean field DCA (mfDCA). On this basis, to enable calculations based on codon sites, some content in the pydca package was modified. Each codon of the original nucleotide sequence was converted into a single character to obtain the codon sequence for computation. A new dictionary was added to the fasta reader of the package for reading the generated codon sequences. The string “codon” has been added to the value of the “biomolecule” parameter in the package, corresponding to a value of 65 for the “num_site_states” parameter. Based on the modified pydca package, the coevolutionary scores between the selected codon sites in *PA, NP, PB1*, and *PB2* genes were calculated separately. Coevolutionary scores were calculated from the direct information ${DI}_{ij}$, which is defined by the following Formula VI:

${DI}_{ij}=\sum_{a=1}^{q-1} \sum_{b=1}^{q-1} P_{ij}^{dir}\left( a,b \right)log\frac{P_{ij}^{dir}\left( a,b \right)}{f_{i}(a)f_{j}(b)}$, (VI)

In Formula VI, $f_{i}(a)$ means the single-site frequency of $a$ and $q-1$ states other than gap states were considered (the value of $q$ is 5 for RNA, 21 for proteins, and 65 for codons).

$P_{ij}^{dir}\left( a,b \right)= \frac{1}{Z_{ij}}\exp\left[ J_{ij}\left( a,b \right)+\tilde{h_{i}}\left( a \right)+\tilde{h_{j}}\left( b \right) \right],$ (VII)

In Formula VII,$Z_{ij}$ is the normalization constant, $\tilde{h_{i}}$ is the single site fields and $J_{ij}\left( a,b \right)$ is the coupling strength of site $i$ and site $j$ for position $a$ and $b$.

**Risk index**

The risk index was defined as the risk of post-adaptation reassortment for the H5N1 serotype IAV with H3N2 reference IAV in year *i* and continent *j*, after simulating reassortment with *k* single gene, with adjustments made for the bias in the number of years and continents. To evaluate the risk index for each year, a risk index algorithm that balances the initial quantity of the virus strain was developed with its adaptive number, which was defined by the following Formula VIII:

$$risk index (i, j, k) = \frac{A_{\mathrm{ik}}}{\sum_{i=1}^{n} A_{\mathrm{ik}}}+\frac{A_{\mathrm{ijk}}}{A_{\mathrm{ik}}}+\frac{ln(B_{i})}{\sum_{i=1}^{n} ln(B_{i})}+\frac{ln(B_{\mathrm{ij}})}{ln(B_{i})}, (VIII)$$

In Formula VIII, *i* is the year, *j* is the continent, *k* is the segment, *n* is the total number of the years, $A_{\mathrm{ik}}$ is the number of adaptive H5N1 viral strains for year *i* and segment *k*, $B_{i}$ is the total number of H5N1 viral strains for year *i*, $B_{ij}$ is the total number of H5N1 viral strains for year *i* and continent *j*, and $A_{ijk}$ is the number of adaptive H5N1 viral strains for year *i*, continent *j* and segment *k*.

**Cell Culture**

HEK293T (Human embryonic kidney) cells (ATCC, CRL-3216) were cultured at 37℃/5% CO2 in Dulbecco’s modiﬁed Eagle’s medium (Thermo Fisher Scientific) containing 10% fetal bovine serum (Gibco), 1% penicillin-streptomycin and 1% Hepes.

**Detection of** **reassortment polymerase activity**

293T cells were seeded in 48-well plates and cultured until reaching approximately 80% confluence, at which point they were co-transfected using the Lipofectamine 3000 reagent (Thermo Fisher Scientific) with a combination of pHW2000 plasmids encoding the PB2, PB1, PA, and NP proteins, with their own original complete UTR region and coding region (representing all possible reassortment combinations), pHH21 plasmids designed to express negative-sense vRNA-like firefly luciferase (Fluc) RNA containing either human or avian IAV untranslated regions (UTRs) at the two ends, and plasmids encoding renilla luciferase (Rluc) to serve as an internal control. Six hours post-transfection, the cells were subjected to three consecutive washes with PBS, after which the culture medium was replaced with fresh medium to ensure optimal conditions for continued expression. At 24 hours post-transfection, the cells were washed again with PBS, lysed, and subsequently analyzed for polymerase activity using the Dual-Luciferase Reporter Assay System kit (Promega, Madison, WI, USA). To compare the relative strength of the polymerase activity after reassortment with that of the control group, the Fluc/Rluc ratios were calculated and then performed normalization. The specific method for normalization was as follows: within the same plate, the raw ratio of each test polymerase activity was divided by the mean raw ratio of the control group to obtain “Relative activity”. All experimental procedures were conducted independently in triplicate to ensure reproducibility and statistical validity.

For the visualization of reassortment polymerase activity via fluorescence, the transfection conditions and methodologies were identical to those described previously, with the exception that the pHH21-Fluc plasmid was substituted with the pHH21-GFP plasmid, and there was no need to transfect the Rluc plasmid. At 24 hours post-transfection, the fluorescence intensity was examined using the Agilent BioTek Cytation 5 imaging system. Images were subsequently captured to document the fluorescence expression, providing a visual representation of polymerase activity under the specified experimental conditions.

**Phylogenetic analysis of IAV**

To investigate the evolutionary dynamics and potential human adaptation of avian H5N1 IAVs, phylogenetic analyses were conducted utilizing polymerase gene sequences, with 100 representative samples randomly selected for each of the PB2, PB1, PA, and NP segments. Initially, all available avian IAV sequences with associated collection dates were aligned using the MAFFT^[17]^ to ensure accurate sequence comparison. Subsequently, maximum likelihood phylogenetic trees were generated employing RAxML (v8.2.12)^[18]^, with 100 bootstrap repetitions, while default parameters were retained for other settings. The resulting phylogenetic trees were then visualized and annotated using the interactive Tree of Life (iTOL) platform^[19]^, enabling a comprehensive and detailed interpretation of the evolutionary relationships of these viral strains.

**Statistical Analysis**

The GraphPad Prism 10 software was used to conduct statistical analysis. Data are presented as means ± SD of at least three independent experiments. Differences between the two groups were evaluated using Student’s t-test. Significance levels were set as follows: * *p* < 0.05; ** *p* < 0.01; *** *p* < 0.001.

**Reference:**

[1] Katoh K, Misawa K, Kuma K, Miyata T. MAFFT: a novel method for rapid multiple sequence alignment based on fast Fourier transform. Nucleic Acids Res. 2002;30(14):3059-3066.

[2] McInnes L, Healy J, Melville J. UMAP: Uniform Manifold Approximation and Projection for Dimension Reduction[J]. 2018.

[3] Müllner D. Modern hierarchical, agglomerative clustering algorithms[J]. 2011.

[4] Jolliffe I T, Cadima J. Principal component analysis: a review and recent developments[J]. Philos Trans A Math Phys Eng Sci, 2016,374(2065):20150202.

[5] Metz C E. Basic principles of ROC analysis[J]. Semin Nucl Med, 1978,8(4):283-298.

[6] Matsuki M, Lago P, Inoue S. Characterizing Word Embeddings for Zero-Shot Sensor-Based Human Activity Recognition[J]. Sensors (Basel), 2019,19(22).

[7] Li J, Wu Y N, Zhang S, et al. Deep learning based on biologically interpretable genome representation predicts two types of human adaptation of SARS-CoV-2 variants[J]. Brief Bioinform, 2022,23(3).

[8] Lin Z, Akin H, Rao R, et al. Evolutionary-scale prediction of atomic-level protein structure with a language model[J]. Science, 2023,379(6637):1123-1130.

[9] Rives A, Meier J, Sercu T, et al. Biological structure and function emerge from scaling unsupervised learning to 250 million protein sequences[J]. Proc Natl Acad Sci U S A, 2021,118(15).

[10] Ji Y, Zhou Z, Liu H, et al. DNABERT: pre-trained Bidirectional Encoder Representations from Transformers model for DNA-language in genome[J]. Bioinformatics, 2021,37(15):2112-2120.

[11] Zhou Z, Ji Y, Li W, et al. DNABERT-2: Efficient Foundation Model and Benchmark For Multi-Species Genome[J]. 2023.

[12] Hou X, He Y, Fang P, et al. Using artificial intelligence to document the hidden RNA virosphere[J]. Cell, 2024,187(24):6929-6942.

[13] Wissler C. THE SPEARMAN CORRELATION FORMULA[J]. Science, 1905,22(558):309-311.

[14] Morcos F, Pagnani A, Lunt B, et al. Direct-coupling analysis of residue coevolution captures native contacts across many protein families[J]. Proc Natl Acad Sci U S A, 2011,108(49):E1293-E1301.

[15] Ekeberg M, Lovkvist C, Lan Y, et al. Improved contact prediction in proteins: using pseudolikelihoods to infer Potts models[J]. Phys Rev E Stat Nonlin Soft Matter Phys, 2013,87(1):12707.

[16] Zerihun M B, Pucci F, Peter E K, et al. pydca v1.0: a comprehensive software for direct coupling analysis of RNA and protein sequences[J]. Bioinformatics, 2020,36(7):2264-2265.

[17] Katoh K, Misawa K, Kuma K, et al. MAFFT: a novel method for rapid multiple sequence alignment based on fast Fourier transform[J]. Nucleic Acids Res, 2002,30(14):3059-3066.

[18] Stamatakis A. RAxML version 8: a tool for phylogenetic analysis and post-analysis of large phylogenies[J]. Bioinformatics, 2014,30(9):1312-1313.

[19] Letunic I, Bork P. Interactive tree of life (iTOL) v3: an online tool for the display and annotation of phylogenetic and other trees[J]. Nucleic Acids Res, 2016,44(W1):W242-W245.
