# Supplementary material for: Deep learning predicts potential reassortments of avian H5N1 with human influenza viruses
Source: Natl Sci Rev. 2025 Sep 17;12(12):nwaf396. doi: 10.1093/nsr/nwaf396 (PMC12707066; doi:10.1093/nsr/nwaf396)
Supplement: nwaf396_Supplemental_Files [file nwaf396_supplemental_files.zip › Supplementary tables.docx]

**Supplementary Table 1. Evaluation results of indicators after clustering using host labels with dimensionality reduction using PCA on 4 segments.**

| Cluster index | Segment | Embedding method | | | | | | | | | |
| --- | --- | --- | --- | --- | --- | --- | --- | --- | --- | --- | --- |
|  |  | non-pretrained | |  | pretrained | | | | |  | Random |
|  |  | Codon2Vec | DCR |  | Word2Vec | LucaOne | ESM2 | ESM2_finetuned | DNABERT2 |  |  |
| ARI | *PB2* | 0.4068 | 0.4055 |  | 0.4068 | 0 | -0.0001 | **0.9821** | 0.0776 |  | -0.0004 |
|  | *PB1* | **0.937** | 0.9331 |  | **0.937** | 0.0007 | 0.0006 | 0.9293 | 0.1503 |  | -0.0004 |
|  | *PA* | **0.4367** | 0.4081 |  | **0.4367** | 0.4235 | 0.0862 | 0.4081 | 0.3055 |  | -0.0004 |
|  | *NP* | 0.4068 | **0.4119** |  | 0.4068 | 0.4068 | 0.4068 | 0.4068 | 0.367 |  | -0.0005 |
| NMI | *PB2* | 0.453 | 0.4522 |  | 0.453 | 0.0001 | 0 | **0.9585** | 0.1919 |  | 0.0001 |
|  | *PB1* | **0.8921** | 0.8871 |  | **0.8921** | 0.0076 | 0.0067 | 0.8787 | 0.2667 |  | 0.0001 |
|  | *PA* | **0.4735** | 0.4539 |  | **0.4735** | 0.4137 | 0.1305 | 0.4539 | 0.3833 |  | 0.0001 |
|  | *NP* | 0.453 | **0.4565** |  | 0.453 | 0.453 | 0.453 | 0.453 | 0.4258 |  | 0 |
| SC | *PB2* | 0.7092 | 0.6832 |  | 0.7092 | **0.8409** | 0.8223 | 0.6666 | 0.4812 |  | 0.3049 |
|  | *PB1* | 0.715 | 0.6974 |  | 0.715 | **0.9577** | 0.9446 | 0.7935 | 0.4342 |  | 0.3049 |
|  | *PA* | 0.7306 | 0.7203 |  | 0.7306 | **0.8161** | 0.5755 | 0.6951 | 0.7053 |  | 0.3049 |
|  | *NP* | 0.7046 | 0.6808 |  | 0.7046 | **0.8889** | 0.7828 | 0.6647 | 0.7288 |  | 0.2972 |
| CH | *PB2* | **4845.732** | 3865.058 |  | **4845.732** | 2657.32 | 2433.704 | 3162.183 | 814.4634 |  | 933.6054 |
|  | *PB1* | 3983.382 | 3256.509 |  | 3983.382 | 4710.246 | **4752.941** | 4501.1201 | 962.351 |  | 933.6054 |
|  | *PA* | **5732.615** | 4563.785 |  | **5732.615** | 1368.615 | 1433.864 | 3814.1717 | 2769.509 |  | 933.6054 |
|  | *NP* | 3342.693 | 2812.234 |  | 3342.693 | 5110.017 | **5656.573** | 2644.3136 | 3367.5467 |  | 897.2425 |
| DB | *PB2* | 0.3631 | 0.3767 |  | 0.3631 | **0.3526** | 0.3576 | 0.6218 | 0.6636 |  | 1.267 |
|  | *PB1* | 0.5552 | 0.606 |  | 0.5552 | 0.5066 | 0.5293 | **0.3291** | 0.9078 |  | 1.267 |
|  | *PA* | **0.3433** | 0.3557 |  | **0.3433** | 0.6049 | 0.6594 | 0.3958 | 0.7143 |  | 1.267 |
|  | *NP* | 0.416 | 0.442 |  | 0.416 | **0.1959** | 0.3234 | 0.4657 | 0.6113 |  | 1.2894 |

**Supplementary Table 2. Evaluation results of indicators after clustering using subtype labels with dimensionality reduction using PCA on 4 segments.**

| Cluster index | Segment | Embedding method | | | | | | | | | |
| --- | --- | --- | --- | --- | --- | --- | --- | --- | --- | --- | --- |
|  |  | non-pretrained | |  | pretrained | | | | |  | Random |
|  |  | Codon2Vec | DCR |  | Word2Vec | LucaOne | ESM2 | ESM2_finetuned | DNABERT2 |  |  |
| ARI | *PB2* | 0.4068 | 0.4055 |  | 0.4068 | 0 | -0.0001 | **0.9821** | 0.0776 |  | -0.0004 |
|  | *PB1* | **0.937** | 0.9331 |  | **0.937** | 0.0007 | 0.0006 | 0.9293 | 0.1503 |  | -0.0004 |
|  | *PA* | **0.4367** | 0.4081 |  | **0.4367** | 0.4235 | 0.0862 | 0.4081 | 0.3055 |  | -0.0004 |
|  | *NP* | 0.4068 | **0.4119** |  | 0.4068 | 0.4068 | 0.4068 | 0.4068 | 0.367 |  | -0.0005 |
| NMI | *PB2* | 0.453 | 0.4522 |  | 0.453 | 0.0001 | 0 | **0.9585** | 0.1919 |  | 0.0001 |
|  | *PB1* | **0.8921** | 0.8871 |  | **0.8921** | 0.0076 | 0.0067 | 0.8787 | 0.2667 |  | 0.0001 |
|  | *PA* | **0.4735** | 0.4539 |  | **0.4735** | 0.4137 | 0.1305 | 0.4539 | 0.3833 |  | 0.0001 |
|  | *NP* | 0.453 | **0.4565** |  | 0.453 | 0.453 | 0.453 | 0.453 | 0.4258 |  | 0 |
| SC | *PB2* | 0.7092 | 0.6832 |  | 0.7092 | **0.8409** | 0.8223 | 0.6666 | 0.4812 |  | 0.3049 |
|  | *PB1* | 0.715 | 0.6974 |  | 0.715 | **0.9577** | 0.9446 | 0.7935 | 0.4342 |  | 0.3049 |
|  | *PA* | 0.7306 | 0.7203 |  | 0.7306 | **0.8161** | 0.5755 | 0.6951 | 0.7053 |  | 0.3049 |
|  | *NP* | 0.7046 | 0.6808 |  | 0.7046 | **0.8889** | 0.7828 | 0.6647 | 0.7288 |  | 0.2972 |
| CH | *PB2* | **4845.732** | 3865.058 |  | **4845.732** | 2657.32 | 2433.704 | 3162.183 | 814.4634 |  | 933.6054 |
|  | *PB1* | 3983.382 | 3256.509 |  | 3983.382 | 4710.246 | **4752.941** | 4501.1201 | 962.351 |  | 933.6054 |
|  | *PA* | **5732.615** | 4563.785 |  | **5732.615** | 1368.615 | 1433.864 | 3814.1717 | 2769.509 |  | 933.6054 |
|  | *NP* | 3342.693 | 2812.234 |  | 3342.693 | 5110.017 | **5656.573** | 2644.3136 | 3367.5467 |  | 897.2425 |
| DB | *PB2* | 0.3631 | 0.3767 |  | 0.3631 | **0.3526** | 0.3576 | 0.6218 | 0.6636 |  | 1.267 |
|  | *PB1* | 0.5552 | 0.606 |  | 0.5552 | 0.5066 | 0.5293 | **0.3291** | 0.9078 |  | 1.267 |
|  | *PA* | **0.3433** | 0.3557 |  | **0.3433** | 0.6049 | 0.6594 | 0.3958 | 0.7143 |  | 1.267 |
|  | *NP* | 0.416 | 0.442 |  | 0.416 | **0.1959** | 0.3234 | 0.4657 | 0.6113 |  | 1.2894 |

**Supplementary Table 3. Evaluation results of indicators after clustering using subtype labels without dimensionality reduction on 4 segments.**

| Cluster index | Segment | Embedding method | | | | | | | | | |
| --- | --- | --- | --- | --- | --- | --- | --- | --- | --- | --- | --- |
|  |  | non-pretrained | |  | pretrained | | | | |  | Random |
|  |  | Codon2Vec | DCR |  | Word2Vec | LucaOne | ESM2 | ESM2_finetuned | DNABERT2 |  |  |
| ARI | *PB2* | 0.2611 | **0.3022** |  | 0.2579 | 0.1881 | 0.1717 | 0.2213 | 0.1193 |  | -0.0035 |
|  | *PB1* | **0.3251** | 0.2885 |  | 0.2902 | 0.1352 | 0.1774 | 0.2301 | 0.1794 |  | -0.0035 |
|  | *PA* | 0.3071 | 0.2751 |  | **0.3146** | 0.1657 | 0.1939 | 0.2438 | 0.0938 |  | -0.0035 |
|  | *NP* | 0.2884 | **0.3117** |  | 0.2961 | 0.2095 | 0.1968 | 0.234 | 0.1958 |  | -0.0002 |
| NMI | *PB2* | 0.4698 | **0.4867** |  | 0.4551 | 0.45 | 0.429 | 0.2213 | 0.2852 |  | 0.0682 |
|  | *PB1* | **0.5024** | 0.4983 |  | 0.4949 | 0.4253 | 0.4384 | 0.2301 | 0.3608 |  | 0.0682 |
|  | *PA* | 0.4774 | 0.4679 |  | **0.4901** | 0.448 | 0.411 | 0.2438 | 0.2714 |  | 0.0682 |
|  | *NP* | 0.4669 | 0.4785 |  | 0.4578 | **0.4861** | 0.4505 | 0.234 | 0.4258 |  | 0.0928 |
| SC | *PB2* | 0.2912 | 0.2559 |  | 0.2657 | 0.1295 | 0.147 | **0.3138** | 0.0421 |  | -0.001 |
|  | *PB1* | 0.2647 | 0.2703 |  | 0.2455 | 0.1921 | 0.1449 | **0.3913** | 0.0539 |  | -0.001 |
|  | *PA* | 0.296 | 0.2342 |  | 0.3305 | 0.1038 | 0.0557 | **0.3767** | 0.054 |  | -0.001 |
|  | *NP* | 0.2583 | 0.2457 |  | 0.2696 | 0.2184 | 0.2539 | **0.454** | 0.0744 |  | -0.001 |
| CH | *PB2* | 53.1981 | 47.4042 |  | **55.126** | 38.9893 | 33.6164 | 45.7591 | 4.8918 |  | 1.0146 |
|  | *PB1* | 54.9104 | 48.5507 |  | 52.8791 | 42.2475 | **126.1776** | 93.2736 | 5.7792 |  | 1.0146 |
|  | *PA* | 48.1204 | 38.6991 |  | 51.9807 | 32.556 | 33.9654 | **75.7403** | 5.684 |  | 1.0146 |
|  | *NP* | 55.7336 | 51.3049 |  | 52.392 | 68.8639 | 65.1261 | **129.7019** | 7.939 |  | 1.0164 |
| DB | *PB2* | 1.4496 | 1.7966 |  | 1.5185 | 2.0025 | 1.9502 | **1.3877** | 4.2507 |  | 6.89 |
|  | *PB1* | 1.8053 | 1.9867 |  | 1.8956 | 1.5381 | 1.5043 | **1.0449** | 3.9273 |  | 6.89 |
|  | *PA* | 1.552 | 1.7863 |  | 1.5693 | 1.6484 | 1.5063 | **1.1099** | 3.7438 |  | 6.89 |
|  | *NP* | 1.9348 | 2.1044 |  | 1.9057 | 1.6624 | 1.8482 | **1.2037** | 3.3294 |  | 7.1064 |

**Supplementary Table 4. Evaluation results of indicators after clustering using host labels without dimensionality reduction on 4 segments.**

| Cluster index | Segment | Embedding method | | | | | | | | | |
| --- | --- | --- | --- | --- | --- | --- | --- | --- | --- | --- | --- |
|  |  | non-pretrained | |  | pretrained | | | | |  | Random |
|  |  | Codon2Vec | DCR |  | Word2Vec | LucaOne | ESM2 | ESM2_finetuned | DNABERT2 |  |  |
| ARI | *PB2* | 0.4068 | 0.4055 |  | 0.4068 | 0.4017 | 0.3879 | 0.4353 | **0.6854** |  | 0.0001 |
|  | *PB1* | **0.9370** | 0.9331 |  | 0.9331 | 0.8968 | 0.8556 | 0.9293 | 0.2058 |  | 0.0001 |
|  | *PA* | 0.4367 | 0.4068 |  | 0.4367 | 0.4235 | **0.5744** | 0.4093 | 0.3122 |  | 0.0001 |
|  | *NP* | **0.9867** | 0.9880 |  | **0.9867** | 0.4110 | 0.4025 | 0.1273 | 0.3693 |  | 0.0022 |
| NMI | *PB2* | 0.4530 | 0.4522 |  | 0.4530 | 0.4495 | 0.3734 | 0.4644 | **0.6544** |  | 0.0008 |
|  | *PB1* | **0.8921** | 0.8871 |  | 0.8871 | 0.8243 | 0.7925 | 0.8787 | 0.3112 |  | 0.0008 |
|  | *PA* | 0.4735 | 0.4530 |  | 0.4735 | 0.4137 | **0.4807** | 0.4548 | 0.3880 |  | 0.0008 |
|  | *NP* | 0.9679 | **0.9708** |  | 0.9679 | 0.4278 | 0.4497 | 0.1914 | 0.4269 |  | 0.0026 |
| SC | *PB2* | 0.3233 | 0.3008 |  | 0.3111 | 0.3954 | **0.5413** | 0.5053 | 0.0351 |  | 0.0001 |
|  | *PB1* | 0.2969 | 0.2680 |  | 0.3088 | 0.2701 | 0.3197 | **0.4936** | 0.0417 |  | 0.0001 |
|  | *PA* | 0.3673 | 0.3222 |  | 0.3647 | **0.4371** | 0.2442 | 0.3972 | 0.0587 |  | 0.0001 |
|  | *NP* | 0.3327 | 0.3193 |  | 0.3150 | **0.5630** | 0.5242 | 0.2042 | 0.0939 |  | 0.0000 |
| CH | *PB2* | 927.6256 | 835.1504 |  | 854.4034 | 457.9445 | 634.6224 | **1422.1690** | 72.2037 |  | 1.0492 |
|  | *PB1* | 805.3344 | 679.9488 |  | 852.8267 | 229.4331 | 191.9719 | **1305.2044** | 96.8550 |  | 1.0492 |
|  | *PA* | **1177.8472** | 945.1821 |  | 1150.4437 | 564.2139 | 367.9609 | 928.1054 | 142.2650 |  | 1.0492 |
|  | *NP* | 700.2746 | 901.7241 |  | 641.6455 | **1237.0291** | 1227.9903 | 214.2648 | 160.4277 |  | 1.1078 |
| DB | *PB2* | 1.1196 | 1.1887 |  | 1.1575 | 1.0456 | 1.0721 | **0.7926** | 5.1381 |  | 27.7173 |
|  | *PB1* | 1.5176 | 1.6588 |  | 1.4712 | 1.7767 | 1.5599 | **0.9417** | 3.6571 |  | 27.7173 |
|  | *PA* | 1.0257 | 1.1240 |  | 1.0293 | 1.2997 | 1.9636 | **1.0191** | 3.2281 |  | 27.7173 |
|  | *NP* | 1.4330 | 1.4592 |  | 1.4978 | 0.7561 | **0.7260** | 2.0057 | 2.6872 |  | 32.1432 |

**Supplementary Table 5. Adaptation prediction and true labels of the naturally reassortant influenza A viruses for validation.**

| Virus strain name | Subtype | True label | Predicted label | Probability |
| --- | --- | --- | --- | --- |
| A/reassortant/FluMist(California/07/2009 x Ann Arbor/6/1960) | H1N1 | 1 | 1 | 0.9999 |
| A/reassortant/NYMC X-147 | H3N2 | 1 | 1 | 0.9975 |
| A/reassortant/NYMC X-151 | H3N2 | 1 | 1 | 1 |
| A/reassortant/NYMC X-153A | H3N2 | 1 | 1 | 0.9981 |
| A/reassortant/NYMC X-155 | H3N2 | 1 | 1 | 0.9959 |
| A/reassortant/NYMC X-157 CL-3 | H3N2 | 1 | 1 | 0.9981 |
| A/reassortant/NYMC X-165 | H3N2 | 1 | 1 | 0.9886 |
| A/reassortant/NYMC X-167 | H3N2 | 1 | 1 | 0.986 |
| A/reassortant/NYMC X-167A | H3N2 | 1 | 1 | 0.985 |
| A/reassortant/NYMC X-167B | H3N2 | 1 | 1 | 1 |
| A/reassortant/NYMC X-167C | H3N2 | 1 | 1 | 1 |
| A/reassortant/NYMC X-169 | H3N2 | 1 | 1 | 0.9978 |
| A/reassortant/NYMC X-169A | H3N2 | 1 | 1 | 0.9889 |
| A/reassortant/NYMC X-173 | H1N1 | 1 | 1 | 0.9981 |
| A/reassortant/NYMC X-173A | H1N1 | 1 | 1 | 0.9979 |
| A/reassortant/NYMC X-173B | H1N1 | 1 | 1 | 0.9981 |
| A/reassortant/NYMC X-173C | H1N1 | 1 | 1 | 0.9981 |
| A/reassortant/NYMC X-175A | H3N2 | 1 | 1 | 0.9896 |
| A/reassortant/NYMC X-175C | H3N2 | 1 | 1 | 0.9975 |
| A/reassortant/NYMC X-177 | H1N1 | 1 | 1 | 0.9982 |
| A/reassortant/NYMC X-177A | H1N1 | 1 | 1 | 0.9975 |
| A/reassortant/NYMC X-177B | H1N1 | 1 | 1 | 0.9981 |
| A/reassortant/NYMC X-179 | H1N1 | 1 | 1 | 0.9989 |
| A/reassortant/NYMC X-179(NYMC X-157 x A/California/07/2009) | H1N1 | 1 | 1 | 0.9992 |
| A/reassortant/NYMC X-179A | H1N1 | 1 | 1 | 0.999 |
| A/reassortant/NYMC X-179A(NYMC X-157 x A/California/07/2009) | H1N1 | 1 | 1 | 0.9993 |
| A/reassortant/NYMC X-181 | H1N1 | 1 | 1 | 0.9993 |
| A/reassortant/NYMC X-181A | H1N1 | 1 | 1 | 0.9993 |
| A/reassortant/NYMC X-181B | H1N1 | 1 | 1 | 0.9992 |
| A/reassortant/NYMC X-183 | H3N2 | 1 | 1 | 0.9975 |
| A/reassortant/NYMC X-185 | H3N2 | 1 | 1 | 0.9983 |
| A/reassortant/NYMC X-185xp | H3N2 | 1 | 1 | 0.9974 |
| A/reassortant/NYMC X-187 | H3N2 | 1 | 1 | 0.9974 |
| A/reassortant/NYMC X-187A | H3N2 | 1 | 1 | 0.9976 |
| A/reassortant/NYMC X-189 | H3N2 | 1 | 1 | 0.9976 |
| A/reassortant/NYMC X-191 | H3N2 | 1 | 1 | 0.9981 |
| A/reassortant/NYMC X-193 | H3N2 | 1 | 1 | 0.9982 |
| A/reassortant/NYMC X-193A | H3N2 | 1 | 1 | 0.9807 |
| A/reassortant/NYMC X-195A | H3N2 | 1 | 1 | 1 |
| A/reassortant/NYMC X-197 | H3N2 | 1 | 1 | 1 |
| A/reassortant/NYMC X-197(Brisbane/11/2010 x Puerto Rico/8/1934) | H3N2 | 1 | 1 | 0.9845 |
| A/reassortant/NYMC X-199 | H3N2 | 1 | 1 | 1 |
| A/reassortant/NYMC X-203(A/Puerto Rico/8/1934 x A/Minnesota/11/2010) | H3N2 | 1 | 1 | 0.9984 |
| A/reassortant/NYMC X-203A(A/Puerto Rico/8/1934 x A/Minnesota/11/2010) | H3N2 | 1 | 1 | 0.9983 |
| A/reassortant/NYMC X-205 | H1N1 | 1 | 0 | 0.4014 |
| A/reassortant/NYMC X-205A | H1N1 | 1 | 1 | 0.9993 |
| A/reassortant/NYMC X-207 | H3N2 | 1 | 1 | 0.998 |
| A/reassortant/NYMC X-207A | H3N2 | 1 | 1 | 0.9981 |
| A/reassortant/NYMC X-211 | H1N1 | 1 | 1 | 0.9699 |
| A/reassortant/NYMC X-211A | H1N1 | 1 | 1 | 0.9699 |
| A/reassortant/NYMC X-211B | H1N1 | 1 | 1 | 0.9691 |
| A/reassortant/NYMC X-213(A/Puerto Rico/8/1934 x A/Indiana/10/2011) | H3N2 | 1 | 1 | 0.9994 |
| A/reassortant/NYMC X-215(A/Puerto Rico/8/1934 x A/Brisbane/299/2011) | H3N2 | 1 | 1 | 0.9987 |
| A/reassortant/NYMC X-217(A/Puerto Rico/8/1934 x A/Victoria/361/2011) | H3N2 | 1 | 1 | 0.9966 |
| A/reassortant/NYMC X-217A(A/Puerto Rico/8/1934 x A/Victoria/361/2011) | H3N2 | 1 | 1 | 0.9974 |
| A/reassortant/NYMC X-221(A/Puerto Rico/8/1934 x A/Ohio/2/2012) | H3N2 | 1 | 1 | 0.9692 |
| A/reassortant/NYMC X-223(A/Puerto Rico/8/1934 x A/Texas/50/2012) | H3N2 | 1 | 1 | 0.9977 |
| A/reassortant/NYMC X-223A(A/Puerto Rico/8/1934 x A/Texas/50/2012) | H3N2 | 1 | 1 | 0.9978 |
| A/reassortant/NYMC X-225(A/Puerto Rico/8/1934 x A/Hawaii/22/2012) | H3N2 | 1 | 1 | 0.9982 |
| A/reassortant/NYMC X-225A(A/Puerto Rico/8/1934 x A/Hawaii/22/2012) | H3N2 | 1 | 1 | 0.9989 |
| A/reassortant/NYMC X-227(A/Puerto Rico/8/1934 x A/Indiana/21/2012) | H3N2 | 1 | 0 | 0.1939 |
| A/reassortant/X-109 | H3N2 | 1 | 1 | 0.9996 |
| A/reassortant/X-119(Puerto Rico/8/1934 x Harbin/15/1992) | H3N2 | 1 | 1 | 1 |
| A/reassortant/X-121 | H3N2 | 1 | 1 | 0.9993 |
| A/reassortant/X-121(Puerto Rico/8/1934 x Shangdong/9/1993) | H3N2 | 1 | 1 | 0.9992 |
| A/reassortant/X-123A | H3N2 | 1 | 1 | 0.9974 |
| A/reassortant/X-129 | H3N2 | 1 | 1 | 0.9987 |
| A/reassortant/X-137 | H3N2 | 1 | 1 | 0.9975 |
| A/reassortant/X-139 | H1N1 | 1 | 1 | 0.9992 |
| A/reassortant/X-139(X-31B x New Caledonia/20/1999) | H1N1 | 1 | 1 | 0.9973 |
| A/reassortant/X-141 | H3N2 | 1 | 1 | 0.9981 |
| A/reassortant/X-143 | H3N2 | 1 | 1 | 0.9983 |
| A/reassortant/X-145 | H3N2 | 1 | 1 | 1 |
| A/reassortant/X-175C(Uruguay/716/2007 x Puerto Rico/8/1934) | H3N2 | 1 | 1 | 0.9974 |
| A/reassortant/X-37 | H3N2 | 1 | 1 | 0.9993 |
| A/reassortant/X-37a | H3N2 | 1 | 1 | 0.9994 |
| A/reassortant/X-41 | H3N2 | 1 | 1 | 1 |
| A/reassortant/X-47 | H3N2 | 1 | 1 | 0.9996 |
| A/reassortant/X-53(Puerto Rico/8/1934 x New Jersey/11/1976) | H1N1 | 1 | 1 | 0.9975 |
| A/reassortant/X-61 | H3N2 | 1 | 1 | 0.999 |
| A/reassortant/X-65 | H1N1 | 1 | 1 | 0.9981 |
| A/reassortant/X-7 | H1N2 | 1 | 1 | 0.9986 |
| A/reassortant/X-73 | H3N2 | 1 | 1 | 0.9992 |

**Supplementary Table 6. Computational expenditure and model performance of various single-segmented models.**

| Model | Segment | Time(s) | Memory usage(GB) | Total memory(GB) |
| --- | --- | --- | --- | --- |
| Codon2Vec+ResNet | *PB2* | 282.927 | 5.954 | 1259.104 |
|  | *PB1* | 272.683 | 6.016 | 1259.104 |
|  | *PA* | 260.935 | 6.022 | 1259.104 |
|  | *NP* | 227.673 | 4.923 | 1259.104 |
| Word2Vec+ResNet | *PB2* | 279.431 | 5.966 | 1259.104 |
|  | *PB1* | 278.896 | 6.039 | 1259.104 |
|  | *PA* | 270.645 | 6.053 | 1259.104 |
|  | *NP* | 232.222 | 4.963 | 1259.104 |
| ESM2+ResNet | *PB2* | 1539.956 | 136.144 | 503.509 |
|  | *PB1* | 2594.422 | 136.976 | 503.509 |
|  | *PA* | 1377.911 | 136.79 | 503.509 |
|  | *NP* | 1298.018 | 92.196 | 503.509 |
| lucaOne+ResNet | *PB2* | 1579.423 | 136.218 | 503.509 |
|  | *PB1* | 2563.993 | 136.465 | 503.509 |
|  | *PA* | 1386.156 | 137.359 | 503.509 |
|  | *NP* | 1303.094 | 92.143 | 503.509 |
| DNABERT2+ResNet | *PB2* | 752.929 | 90.938 | 503.509 |
|  | *PB1* | 877.863 | 80.947 | 503.509 |
|  | *PA* | 821.422 | 64.895 | 503.509 |
|  | *NP* | 300.007 | 28.187 | 503.509 |

**Supplementary Table 7. The importance of codons derived from ablation experiments**

| Location | Gene | | | | | | | |
| --- | --- | --- | --- | --- | --- | --- | --- | --- |
|  | *PB2* | | *PB1* | | *PA* | | *NP* | |
|  | Bayes | HAIRANGE | Bayes | HAIRANGE | Bayes | HAIRANGE | Bayes | HAIRANGE |
| 1 | 0.4657 | 0.0991 | 0.4211 | 0.0152 | 0.2572 | 0.4562 | 0.0605 | 0.2077 |
| 2 | 0.4599 | 0.1025 | 0.4227 | 0.0161 | 0.2238 | 0.4522 | 0.1031 | 0.2053 |
| 3 | 0.4723 | 0.1059 | 0.4246 | 0.0170 | 0.2028 | 0.4483 | 0.1323 | 0.2031 |
| 4 | 0.4729 | 0.1094 | 0.4246 | 0.0180 | 0.2223 | 0.4444 | 0.1323 | 0.2012 |
| 5 | 0.4730 | 0.1129 | 0.3485 | 0.0190 | 0.2225 | 0.4406 | 0.1323 | 0.1995 |
| 6 | 0.4325 | 0.1164 | 0.3485 | 0.0201 | 0.2242 | 0.4369 | 0.1343 | 0.1981 |
| 7 | 0.4289 | 0.1200 | 0.4201 | 0.0212 | 0.2314 | 0.4333 | 0.0731 | 0.1969 |
| 8 | 0.4485 | 0.1236 | 0.4243 | 0.0224 | 0.2232 | 0.4299 | 0.1010 | 0.1960 |
| 9 | 0.5467 | 0.1273 | 0.4261 | 0.0236 | 0.2146 | 0.4265 | 0.1004 | 0.1953 |
| 10 | 0.5405 | 0.1310 | 0.4942 | 0.0249 | 0.2198 | 0.4233 | 0.1003 | 0.1950 |
| 11 | 0.5405 | 0.1348 | 0.4339 | 0.0263 | 0.2545 | 0.4202 | 0.1524 | 0.1949 |
| 12 | 0.5129 | 0.1386 | 0.4224 | 0.0277 | 0.2774 | 0.4172 | 0.1427 | 0.1951 |
| 13 | 0.5119 | 0.1425 | 0.4112 | 0.0292 | 0.2744 | 0.4144 | 0.1427 | 0.1957 |
| 14 | 0.4859 | 0.1465 | 0.3866 | 0.0308 | 0.2534 | 0.4118 | 0.1385 | 0.1965 |
| 15 | 0.4866 | 0.1505 | 0.3868 | 0.0325 | 0.2730 | 0.4094 | 0.1267 | 0.1977 |
| 16 | 0.4863 | 0.1546 | 0.3872 | 0.0342 | 0.2730 | 0.4071 | 0.0950 | 0.1991 |
| 17 | 0.4917 | 0.1588 | 0.3796 | 0.0360 | 0.2970 | 0.4050 | 0.0161 | 0.2008 |
| 18 | 0.4848 | 0.1630 | 0.3071 | 0.0379 | 0.2596 | 0.4031 | 0.0587 | 0.2028 |
| 19 | 0.4462 | 0.1673 | 0.3065 | 0.0399 | 0.2732 | 0.4013 | 0.0604 | 0.2051 |
| 20 | 0.3576 | 0.1717 | 0.3174 | 0.0420 | 0.2731 | 0.3998 | 0.0314 | 0.2077 |
| 21 | 0.3522 | 0.1762 | 0.3174 | 0.0441 | 0.2733 | 0.3984 | 0.0315 | 0.2105 |
| 22 | 0.3456 | 0.1807 | 0.3172 | 0.0463 | 0.2734 | 0.3972 | 0.0439 | 0.2135 |
| 23 | 0.4112 | 0.1853 | 0.3172 | 0.0486 | 0.2832 | 0.3963 | 0.0437 | 0.2169 |
| 24 | 0.4017 | 0.1900 | 0.3168 | 0.0510 | 0.2832 | 0.3955 | 0.0000 | 0.2205 |
| 25 | 0.4328 | 0.1948 | 0.3230 | 0.0535 | 0.2825 | 0.3950 | 0.0118 | 0.2243 |
| 26 | 0.4328 | 0.1996 | 0.3234 | 0.0561 | 0.3438 | 0.3947 | 0.0053 | 0.2284 |
| 27 | 0.4273 | 0.2046 | 0.3180 | 0.0587 | 0.3423 | 0.3945 | 0.0053 | 0.2327 |
| 28 | 0.4275 | 0.2096 | 0.3180 | 0.0615 | 0.3488 | 0.3946 | 0.0202 | 0.2373 |
| 29 | 0.4286 | 0.2148 | 0.2415 | 0.0643 | 0.3489 | 0.3949 | 0.0624 | 0.2421 |
| 30 | 0.4120 | 0.2200 | 0.2415 | 0.0672 | 0.3596 | 0.3954 | 0.0223 | 0.2471 |
| 31 | 0.4310 | 0.2253 | 0.2416 | 0.0702 | 0.3575 | 0.3961 | 0.0397 | 0.2523 |
| 32 | 0.3842 | 0.2308 | 0.1625 | 0.0732 | 0.3319 | 0.3970 | 0.0274 | 0.2578 |
| 33 | 0.4293 | 0.2363 | 0.0771 | 0.0764 | 0.3304 | 0.3981 | 0.0901 | 0.2634 |
| 34 | 0.4290 | 0.2420 | 0.0771 | 0.0796 | 0.3218 | 0.3994 | 0.0998 | 0.2693 |
| 35 | 0.4392 | 0.2477 | 0.0689 | 0.0829 | 0.3271 | 0.4009 | 0.0996 | 0.2753 |
| 36 | 0.4273 | 0.2536 | 0.0697 | 0.0862 | 0.3316 | 0.4026 | 0.0758 | 0.2815 |
| 37 | 0.4422 | 0.2595 | 0.0374 | 0.0897 | 0.3432 | 0.4045 | 0.0753 | 0.2879 |
| 38 | 0.3935 | 0.2655 | 0.0375 | 0.0931 | 0.3432 | 0.4066 | 0.0739 | 0.2945 |
| 39 | 0.3847 | 0.2717 | 0.0355 | 0.0967 | 0.3432 | 0.4089 | 0.0738 | 0.3012 |
| 40 | 0.4062 | 0.2779 | 0.1056 | 0.1003 | 0.3542 | 0.4114 | 0.0738 | 0.3081 |
| 41 | 0.3133 | 0.2842 | 0.1092 | 0.1039 | 0.3474 | 0.4141 | 0.1376 | 0.3151 |
| 42 | 0.3122 | 0.2906 | 0.1090 | 0.1076 | 0.3646 | 0.4169 | 0.1693 | 0.3223 |
| 43 | 0.3902 | 0.2971 | 0.1692 | 0.1113 | 0.3781 | 0.4198 | 0.1518 | 0.3295 |
| 44 | 0.4423 | 0.3037 | 0.1769 | 0.1151 | 0.4356 | 0.4229 | 0.1516 | 0.3369 |
| 45 | 0.4569 | 0.3103 | 0.1769 | 0.1189 | 0.4317 | 0.4262 | 0.1502 | 0.3445 |
| 46 | 0.4167 | 0.3171 | 0.0987 | 0.1228 | 0.4317 | 0.4295 | 0.1491 | 0.3521 |
| 47 | 0.4167 | 0.3240 | 0.0686 | 0.1266 | 0.4317 | 0.4329 | 0.1907 | 0.3599 |
| 48 | 0.3829 | 0.3310 | 0.0683 | 0.1306 | 0.4315 | 0.4365 | 0.2004 | 0.3677 |
| 49 | 0.3829 | 0.3380 | 0.0598 | 0.1345 | 0.4118 | 0.4401 | 0.2004 | 0.3758 |
| 50 | 0.3829 | 0.3452 | 0.0598 | 0.1385 | 0.4118 | 0.4438 | 0.2020 | 0.3839 |
| 51 | 0.3830 | 0.3525 | 0.0395 | 0.1425 | 0.4205 | 0.4476 | 0.2592 | 0.3921 |
| 52 | 0.3829 | 0.3598 | 0.0345 | 0.1466 | 0.4674 | 0.4515 | 0.2496 | 0.4004 |
| 53 | 0.3829 | 0.3672 | 0.0201 | 0.1506 | 0.4669 | 0.4554 | 0.2491 | 0.4089 |
| 54 | 0.3827 | 0.3747 | 0.0570 | 0.1547 | 0.5147 | 0.4595 | 0.2672 | 0.4175 |
| 55 | 0.3733 | 0.3823 | 0.0570 | 0.1588 | 0.5656 | 0.4635 | 0.3299 | 0.4262 |
| 56 | 0.3730 | 0.3899 | 0.0562 | 0.1630 | 0.5646 | 0.4676 | 0.3776 | 0.4350 |
| 57 | 0.3024 | 0.3977 | 0.0558 | 0.1671 | 0.6128 | 0.4718 | 0.3774 | 0.4439 |
| 58 | 0.3638 | 0.4055 | 0.0003 | 0.1713 | 0.5621 | 0.4760 | 0.3512 | 0.4530 |
| 59 | 0.3972 | 0.4134 | 0.0000 | 0.1756 | 0.5614 | 0.4803 | 0.3405 | 0.4621 |
| 60 | 0.3958 | 0.4214 | 0.0120 | 0.1799 | 0.5614 | 0.4846 | 0.3405 | 0.4714 |
| 61 | 0.3706 | 0.4295 | 0.0119 | 0.1842 | 0.5614 | 0.4890 | 0.3384 | 0.4808 |
| 62 | 0.3699 | 0.4377 | 0.0780 | 0.1885 | 0.5284 | 0.4933 | 0.3384 | 0.4903 |
| 63 | 0.3698 | 0.4460 | 0.0767 | 0.1929 | 0.5340 | 0.4977 | 0.3387 | 0.4999 |
| 64 | 0.5074 | 0.4545 | 0.1488 | 0.1973 | 0.6034 | 0.5021 | 0.3986 | 0.5096 |
| 65 | 0.5039 | 0.4630 | 0.1991 | 0.2018 | 0.6367 | 0.5066 | 0.4380 | 0.5194 |
| 66 | 0.4828 | 0.4717 | 0.1970 | 0.2064 | 0.6792 | 0.5110 | 0.4381 | 0.5293 |
| 67 | 0.5016 | 0.4805 | 0.1962 | 0.2110 | 0.6585 | 0.5155 | 0.4597 | 0.5392 |
| 68 | 0.5642 | 0.4894 | 0.1728 | 0.2157 | 0.6512 | 0.5200 | 0.4565 | 0.5492 |
| 69 | 0.6380 | 0.4984 | 0.1728 | 0.2204 | 0.6512 | 0.5245 | 0.4564 | 0.5592 |
| 70 | 0.6191 | 0.5075 | 0.1705 | 0.2253 | 0.6511 | 0.5290 | 0.5209 | 0.5694 |
| 71 | 0.6230 | 0.5168 | 0.1026 | 0.2302 | 0.7261 | 0.5335 | 0.5209 | 0.5795 |
| 72 | 0.5794 | 0.5261 | 0.1032 | 0.2352 | 0.7504 | 0.5380 | 0.4946 | 0.5897 |
| 73 | 0.5390 | 0.5356 | 0.1389 | 0.2402 | 0.7921 | 0.5425 | 0.4354 | 0.5999 |
| 74 | 0.5421 | 0.5451 | 0.0703 | 0.2454 | 0.7873 | 0.5470 | 0.4607 | 0.6102 |
| 75 | 0.5365 | 0.5547 | 0.0668 | 0.2507 | 0.7876 | 0.5515 | 0.4187 | 0.6204 |
| 76 | 0.4533 | 0.5644 | 0.1245 | 0.2560 | 0.7875 | 0.5560 | 0.4863 | 0.6307 |
| 77 | 0.4901 | 0.5742 | 0.1240 | 0.2615 | 0.7323 | 0.5605 | 0.4838 | 0.6409 |
| 78 | 0.5161 | 0.5841 | 0.1846 | 0.2671 | 0.7323 | 0.5649 | 0.4767 | 0.6511 |
| 79 | 0.5460 | 0.5940 | 0.1843 | 0.2728 | 0.7202 | 0.5693 | 0.4728 | 0.6613 |
| 80 | 0.5439 | 0.6040 | 0.1841 | 0.2786 | 0.7469 | 0.5737 | 0.4426 | 0.6713 |
| 81 | 0.5883 | 0.6141 | 0.1709 | 0.2846 | 0.6741 | 0.5780 | 0.4461 | 0.6813 |
| 82 | 0.6553 | 0.6241 | 0.1709 | 0.2906 | 0.6740 | 0.5822 | 0.3930 | 0.6912 |
| 83 | 0.6553 | 0.6343 | 0.1725 | 0.2968 | 0.6587 | 0.5864 | 0.3924 | 0.7011 |
| 84 | 0.7363 | 0.6444 | 0.1615 | 0.3031 | 0.6715 | 0.5906 | 0.4154 | 0.7108 |
| 85 | 0.7463 | 0.6545 | 0.1710 | 0.3096 | 0.6712 | 0.5946 | 0.4047 | 0.7204 |
| 86 | 0.7538 | 0.6646 | 0.1635 | 0.3161 | 0.6715 | 0.5986 | 0.3555 | 0.7298 |
| 87 | 0.7677 | 0.6747 | 0.1633 | 0.3228 | 0.6423 | 0.6024 | 0.3290 | 0.7392 |
| 88 | 0.6709 | 0.6848 | 0.1517 | 0.3295 | 0.6423 | 0.6062 | 0.3678 | 0.7483 |
| 89 | 0.6712 | 0.6948 | 0.2195 | 0.3364 | 0.6430 | 0.6098 | 0.3100 | 0.7573 |
| 90 | 0.6712 | 0.7048 | 0.2921 | 0.3434 | 0.6193 | 0.6133 | 0.3139 | 0.7661 |
| 91 | 0.6753 | 0.7147 | 0.3751 | 0.3504 | 0.6413 | 0.6166 | 0.3137 | 0.7748 |
| 92 | 0.6752 | 0.7245 | 0.3751 | 0.3576 | 0.6383 | 0.6198 | 0.2882 | 0.7832 |
| 93 | 0.6718 | 0.7342 | 0.3819 | 0.3647 | 0.6379 | 0.6228 | 0.2429 | 0.7915 |
| 94 | 0.6962 | 0.7438 | 0.3818 | 0.3719 | 0.6214 | 0.6257 | 0.3446 | 0.7995 |
| 95 | 0.6707 | 0.7533 | 0.3818 | 0.3791 | 0.6195 | 0.6284 | 0.3903 | 0.8073 |
| 96 | 0.7601 | 0.7627 | 0.3816 | 0.3864 | 0.6452 | 0.6308 | 0.4229 | 0.8149 |
| 97 | 0.7148 | 0.7719 | 0.3817 | 0.3936 | 0.7135 | 0.6331 | 0.3475 | 0.8222 |
| 98 | 0.7151 | 0.7810 | 0.3816 | 0.4008 | 0.7410 | 0.6352 | 0.3103 | 0.8294 |
| 99 | 0.7152 | 0.7900 | 0.3910 | 0.4080 | 0.7198 | 0.6372 | 0.3535 | 0.8362 |
| 100 | 0.7230 | 0.7988 | 0.3909 | 0.4152 | 0.7971 | 0.6389 | 0.4409 | 0.8429 |
| 101 | 0.6864 | 0.8075 | 0.3954 | 0.4223 | 0.7912 | 0.6404 | 0.4148 | 0.8494 |
| 102 | 0.7354 | 0.8160 | 0.3944 | 0.4294 | 0.7583 | 0.6417 | 0.4048 | 0.8556 |
| 103 | 0.7422 | 0.8243 | 0.3963 | 0.4364 | 0.7902 | 0.6428 | 0.4024 | 0.8616 |
| 104 | 0.7263 | 0.8325 | 0.4682 | 0.4434 | 0.7846 | 0.6437 | 0.4024 | 0.8673 |
| 105 | 0.7735 | 0.8406 | 0.4646 | 0.4502 | 0.7903 | 0.6444 | 0.3332 | 0.8729 |
| 106 | 0.7739 | 0.8484 | 0.5181 | 0.4570 | 0.8262 | 0.6449 | 0.2930 | 0.8782 |
| 107 | 0.6948 | 0.8561 | 0.5956 | 0.4638 | 0.7614 | 0.6451 | 0.3183 | 0.8833 |
| 108 | 0.6643 | 0.8636 | 0.5953 | 0.4704 | 0.7038 | 0.6451 | 0.3815 | 0.8882 |
| 109 | 0.5736 | 0.8709 | 0.5959 | 0.4769 | 0.7011 | 0.6450 | 0.4077 | 0.8929 |
| 110 | 0.6056 | 0.8781 | 0.6712 | 0.4834 | 0.7011 | 0.6445 | 0.4727 | 0.8975 |
| 111 | 0.6568 | 0.8851 | 0.6209 | 0.4897 | 0.7118 | 0.6439 | 0.4314 | 0.9018 |
| 112 | 0.6901 | 0.8919 | 0.6209 | 0.4960 | 0.7118 | 0.6431 | 0.4320 | 0.9059 |
| 113 | 0.7193 | 0.8985 | 0.6338 | 0.5022 | 0.6954 | 0.6420 | 0.4320 | 0.9099 |
| 114 | 0.7012 | 0.9049 | 0.6335 | 0.5083 | 0.6936 | 0.6408 | 0.4495 | 0.9137 |
| 115 | 0.6712 | 0.9111 | 0.6491 | 0.5143 | 0.6942 | 0.6393 | 0.4348 | 0.9173 |
| 116 | 0.6705 | 0.9171 | 0.6516 | 0.5202 | 0.7226 | 0.6377 | 0.5052 | 0.9207 |
| 117 | 0.6695 | 0.9229 | 0.6660 | 0.5261 | 0.7231 | 0.6359 | 0.5043 | 0.9240 |
| 118 | 0.6638 | 0.9285 | 0.7083 | 0.5318 | 0.7090 | 0.6338 | 0.5170 | 0.9271 |
| 119 | 0.6504 | 0.9339 | 0.7038 | 0.5375 | 0.6575 | 0.6316 | 0.4924 | 0.9300 |
| 120 | 0.6747 | 0.9392 | 0.6872 | 0.5431 | 0.6584 | 0.6292 | 0.4447 | 0.9327 |
| 121 | 0.6726 | 0.9442 | 0.6874 | 0.5486 | 0.6097 | 0.6267 | 0.4992 | 0.9352 |
| 122 | 0.6047 | 0.9490 | 0.6622 | 0.5540 | 0.6108 | 0.6240 | 0.5253 | 0.9376 |
| 123 | 0.5714 | 0.9536 | 0.6554 | 0.5593 | 0.6113 | 0.6211 | 0.5761 | 0.9398 |
| 124 | 0.5724 | 0.9580 | 0.6434 | 0.5646 | 0.5922 | 0.6181 | 0.5749 | 0.9418 |
| 125 | 0.5400 | 0.9622 | 0.6365 | 0.5698 | 0.6203 | 0.6149 | 0.5514 | 0.9436 |
| 126 | 0.5406 | 0.9662 | 0.5690 | 0.5749 | 0.6210 | 0.6116 | 0.6147 | 0.9452 |
| 127 | 0.5404 | 0.9700 | 0.5702 | 0.5800 | 0.6170 | 0.6082 | 0.5972 | 0.9466 |
| 128 | 0.4996 | 0.9735 | 0.6210 | 0.5849 | 0.6178 | 0.6047 | 0.6432 | 0.9478 |
| 129 | 0.5030 | 0.9768 | 0.5706 | 0.5898 | 0.5844 | 0.6010 | 0.6050 | 0.9488 |
| 130 | 0.4670 | 0.9799 | 0.5648 | 0.5947 | 0.5986 | 0.5972 | 0.6691 | 0.9495 |
| 131 | 0.4432 | 0.9828 | 0.5656 | 0.5994 | 0.5986 | 0.5934 | 0.6604 | 0.9501 |
| 132 | 0.3786 | 0.9855 | 0.5889 | 0.6041 | 0.6360 | 0.5894 | 0.6633 | 0.9504 |
| 133 | 0.3192 | 0.9879 | 0.5853 | 0.6087 | 0.6016 | 0.5853 | 0.7076 | 0.9505 |
| 134 | 0.3218 | 0.9901 | 0.5792 | 0.6132 | 0.6074 | 0.5811 | 0.6666 | 0.9503 |
| 135 | 0.3403 | 0.9922 | 0.5228 | 0.6176 | 0.5324 | 0.5767 | 0.6642 | 0.9498 |
| 136 | 0.3447 | 0.9940 | 0.4874 | 0.6219 | 0.4806 | 0.5723 | 0.7254 | 0.9491 |
| 137 | 0.3378 | 0.9955 | 0.4763 | 0.6262 | 0.4205 | 0.5678 | 0.7853 | 0.9481 |
| 138 | 0.3406 | 0.9969 | 0.4761 | 0.6303 | 0.4568 | 0.5631 | 0.7704 | 0.9469 |
| 139 | 0.3776 | 0.9980 | 0.5587 | 0.6343 | 0.4234 | 0.5584 | 0.8125 | 0.9453 |
| 140 | 0.3457 | 0.9988 | 0.5133 | 0.6382 | 0.4478 | 0.5535 | 0.7544 | 0.9434 |
| 141 | 0.3099 | 0.9995 | 0.5137 | 0.6420 | 0.5012 | 0.5485 | 0.7567 | 0.9413 |
| 142 | 0.3065 | 0.9998 | 0.4552 | 0.6457 | 0.5135 | 0.5433 | 0.7601 | 0.9388 |
| 143 | 0.3637 | 1.0000 | 0.4000 | 0.6492 | 0.5061 | 0.5381 | 0.7834 | 0.9361 |
| 144 | 0.4448 | 0.9999 | 0.4001 | 0.6526 | 0.4794 | 0.5327 | 0.7841 | 0.9331 |
| 145 | 0.4281 | 0.9996 | 0.4207 | 0.6558 | 0.5245 | 0.5272 | 0.8110 | 0.9299 |
| 146 | 0.3440 | 0.9989 | 0.4899 | 0.6589 | 0.5245 | 0.5216 | 0.8401 | 0.9264 |
| 147 | 0.3344 | 0.9981 | 0.4900 | 0.6618 | 0.5017 | 0.5159 | 0.8403 | 0.9226 |
| 148 | 0.3387 | 0.9970 | 0.5575 | 0.6646 | 0.4935 | 0.5101 | 0.7822 | 0.9186 |
| 149 | 0.3398 | 0.9956 | 0.5422 | 0.6672 | 0.4939 | 0.5041 | 0.7930 | 0.9143 |
| 150 | 0.3583 | 0.9940 | 0.6107 | 0.6696 | 0.4937 | 0.4980 | 0.8341 | 0.9098 |
| 151 | 0.3446 | 0.9921 | 0.6110 | 0.6718 | 0.4941 | 0.4918 | 0.8606 | 0.9051 |
| 152 | 0.4182 | 0.9900 | 0.6213 | 0.6738 | 0.4979 | 0.4854 | 0.8595 | 0.9002 |
| 153 | 0.3831 | 0.9876 | 0.5309 | 0.6756 | 0.4954 | 0.4790 | 0.8838 | 0.8950 |
| 154 | 0.3829 | 0.9850 | 0.5255 | 0.6772 | 0.4794 | 0.4724 | 0.8830 | 0.8897 |
| 155 | 0.3839 | 0.9822 | 0.4814 | 0.6786 | 0.4590 | 0.4657 | 0.9467 | 0.8841 |
| 156 | 0.3825 | 0.9791 | 0.4813 | 0.6797 | 0.4004 | 0.4589 | 1.0000 | 0.8784 |
| 157 | 0.3724 | 0.9757 | 0.4706 | 0.6806 | 0.4336 | 0.4520 | 0.9999 | 0.8725 |
| 158 | 0.3502 | 0.9721 | 0.4703 | 0.6812 | 0.4675 | 0.4450 | 0.9385 | 0.8664 |
| 159 | 0.3567 | 0.9683 | 0.4622 | 0.6817 | 0.4295 | 0.4379 | 0.8754 | 0.8602 |
| 160 | 0.2386 | 0.9643 | 0.4602 | 0.6819 | 0.4276 | 0.4307 | 0.8587 | 0.8538 |
| 161 | 0.2394 | 0.9600 | 0.4587 | 0.6818 | 0.3603 | 0.4234 | 0.8712 | 0.8472 |
| 162 | 0.2407 | 0.9555 | 0.4568 | 0.6815 | 0.3199 | 0.4161 | 0.9150 | 0.8406 |
| 163 | 0.3385 | 0.9508 | 0.4586 | 0.6810 | 0.3603 | 0.4087 | 0.8720 | 0.8338 |
| 164 | 0.3426 | 0.9458 | 0.4586 | 0.6801 | 0.3256 | 0.4012 | 0.8084 | 0.8268 |
| 165 | 0.3616 | 0.9407 | 0.4405 | 0.6791 | 0.3314 | 0.3936 | 0.8350 | 0.8198 |
| 166 | 0.3603 | 0.9354 | 0.4403 | 0.6778 | 0.3319 | 0.3860 | 0.8428 | 0.8126 |
| 167 | 0.3617 | 0.9300 | 0.4399 | 0.6764 | 0.2999 | 0.3783 | 0.8453 | 0.8053 |
| 168 | 0.3486 | 0.9243 | 0.3668 | 0.6746 | 0.2929 | 0.3706 | 0.8592 | 0.7979 |
| 169 | 0.3415 | 0.9185 | 0.3705 | 0.6727 | 0.3340 | 0.3628 | 0.8799 | 0.7904 |
| 170 | 0.3417 | 0.9125 | 0.3137 | 0.6705 | 0.2860 | 0.3550 | 0.8721 | 0.7828 |
| 171 | 0.3427 | 0.9064 | 0.2372 | 0.6681 | 0.3263 | 0.3472 | 0.8441 | 0.7751 |
| 172 | 0.3202 | 0.9001 | 0.2365 | 0.6655 | 0.3259 | 0.3395 | 0.7512 | 0.7672 |
| 173 | 0.3895 | 0.8936 | 0.2364 | 0.6628 | 0.3266 | 0.3318 | 0.7864 | 0.7593 |
| 174 | 0.3855 | 0.8870 | 0.1610 | 0.6599 | 0.3231 | 0.3241 | 0.7591 | 0.7512 |
| 175 | 0.3219 | 0.8803 | 0.1578 | 0.6569 | 0.3008 | 0.3164 | 0.7897 | 0.7430 |
| 176 | 0.3224 | 0.8734 | 0.1462 | 0.6537 | 0.3127 | 0.3088 | 0.7902 | 0.7348 |
| 177 | 0.3031 | 0.8664 | 0.1464 | 0.6504 | 0.2976 | 0.3012 | 0.7866 | 0.7264 |
| 178 | 0.3207 | 0.8592 | 0.1465 | 0.6469 | 0.2995 | 0.2937 | 0.7674 | 0.7179 |
| 179 | 0.3470 | 0.8520 | 0.1492 | 0.6434 | 0.2997 | 0.2863 | 0.7806 | 0.7093 |
| 180 | 0.3731 | 0.8446 | 0.2128 | 0.6398 | 0.2239 | 0.2789 | 0.7194 | 0.7006 |
| 181 | 0.3715 | 0.8371 | 0.2127 | 0.6361 | 0.2234 | 0.2716 | 0.7837 | 0.6918 |
| 182 | 0.3748 | 0.8295 | 0.1335 | 0.6324 | 0.1897 | 0.2644 | 0.7526 | 0.6829 |
| 183 | 0.3440 | 0.8217 | 0.1373 | 0.6286 | 0.1900 | 0.2573 | 0.7485 | 0.6739 |
| 184 | 0.3065 | 0.8139 | 0.2193 | 0.6247 | 0.2138 | 0.2502 | 0.7476 | 0.6647 |
| 185 | 0.3796 | 0.8060 | 0.3013 | 0.6208 | 0.2189 | 0.2433 | 0.6922 | 0.6555 |
| 186 | 0.4179 | 0.7980 | 0.2993 | 0.6168 | 0.2915 | 0.2364 | 0.7042 | 0.6461 |
| 187 | 0.4110 | 0.7898 | 0.3056 | 0.6127 | 0.2994 | 0.2296 | 0.6368 | 0.6366 |
| 188 | 0.4095 | 0.7815 | 0.2966 | 0.6086 | 0.3185 | 0.2230 | 0.6382 | 0.6270 |
| 189 | 0.4929 | 0.7732 | 0.3683 | 0.6044 | 0.2903 | 0.2164 | 0.6380 | 0.6172 |
| 190 | 0.5246 | 0.7647 | 0.3698 | 0.6002 | 0.2905 | 0.2099 | 0.5739 | 0.6073 |
| 191 | 0.5243 | 0.7560 | 0.3964 | 0.5959 | 0.2964 | 0.2035 | 0.5915 | 0.5973 |
| 192 | 0.4663 | 0.7473 | 0.3028 | 0.5916 | 0.2968 | 0.1972 | 0.5275 | 0.5871 |
| 193 | 0.4660 | 0.7384 | 0.3029 | 0.5872 | 0.2966 | 0.1911 | 0.5260 | 0.5768 |
| 194 | 0.5275 | 0.7294 | 0.3103 | 0.5828 | 0.2399 | 0.1850 | 0.4839 | 0.5664 |
| 195 | 0.5807 | 0.7202 | 0.3103 | 0.5782 | 0.2818 | 0.1790 | 0.5092 | 0.5559 |
| 196 | 0.5305 | 0.7110 | 0.3104 | 0.5736 | 0.2516 | 0.1731 | 0.5095 | 0.5452 |
| 197 | 0.5226 | 0.7016 | 0.3135 | 0.5690 | 0.2861 | 0.1674 | 0.4576 | 0.5344 |
| 198 | 0.5396 | 0.6921 | 0.3122 | 0.5642 | 0.2804 | 0.1617 | 0.4386 | 0.5235 |
| 199 | 0.5755 | 0.6825 | 0.3691 | 0.5594 | 0.3233 | 0.1561 | 0.4944 | 0.5124 |
| 200 | 0.6367 | 0.6728 | 0.4042 | 0.5545 | 0.3508 | 0.1506 | 0.4247 | 0.5013 |
| 201 | 0.6455 | 0.6630 | 0.3767 | 0.5494 | 0.3692 | 0.1452 | 0.4246 | 0.4901 |
| 202 | 0.6458 | 0.6531 | 0.3773 | 0.5443 | 0.3329 | 0.1398 | 0.4082 | 0.4788 |
| 203 | 0.5149 | 0.6430 | 0.2986 | 0.5391 | 0.3330 | 0.1346 | 0.3843 | 0.4674 |
| 204 | 0.5658 | 0.6329 | 0.2988 | 0.5338 | 0.3784 | 0.1295 | 0.4443 | 0.4560 |
| 205 | 0.5657 | 0.6226 | 0.2989 | 0.5283 | 0.3786 | 0.1244 | 0.4433 | 0.4445 |
| 206 | 0.5691 | 0.6123 | 0.3162 | 0.5228 | 0.3656 | 0.1195 | 0.4497 | 0.4330 |
| 207 | 0.5132 | 0.6019 | 0.3714 | 0.5171 | 0.3479 | 0.1146 | 0.4304 | 0.4215 |
| 208 | 0.4318 | 0.5913 | 0.4151 | 0.5113 | 0.3479 | 0.1098 | 0.4284 | 0.4100 |
| 209 | 0.4004 | 0.5807 | 0.4232 | 0.5054 | 0.3512 | 0.1051 | 0.4788 | 0.3984 |
| 210 | 0.4170 | 0.5700 | 0.3662 | 0.4993 | 0.4304 | 0.1005 | 0.4813 | 0.3869 |
| 211 | 0.4849 | 0.5593 | 0.4092 | 0.4931 | 0.4549 | 0.0960 | 0.4817 | 0.3754 |
| 212 | 0.4854 | 0.5485 | 0.3708 | 0.4868 | 0.4487 | 0.0916 | 0.5175 | 0.3639 |
| 213 | 0.5358 | 0.5376 | 0.4367 | 0.4804 | 0.4475 | 0.0874 | 0.4930 | 0.3525 |
| 214 | 0.5174 | 0.5268 | 0.4091 | 0.4738 | 0.4461 | 0.0832 | 0.4953 | 0.3412 |
| 215 | 0.5452 | 0.5158 | 0.4684 | 0.4671 | 0.4457 | 0.0791 | 0.4956 | 0.3299 |
| 216 | 0.5043 | 0.5049 | 0.5527 | 0.4604 | 0.4416 | 0.0752 | 0.4760 | 0.3187 |
| 217 | 0.5940 | 0.4940 | 0.5032 | 0.4535 | 0.4442 | 0.0714 | 0.5520 | 0.3077 |
| 218 | 0.6007 | 0.4830 | 0.4356 | 0.4465 | 0.4522 | 0.0677 | 0.5831 | 0.2967 |
| 219 | 0.5895 | 0.4721 | 0.4021 | 0.4394 | 0.4521 | 0.0642 | 0.5192 | 0.2859 |
| 220 | 0.6283 | 0.4612 | 0.4017 | 0.4323 | 0.4522 | 0.0608 | 0.4487 | 0.2752 |
| 221 | 0.6329 | 0.4503 | 0.4164 | 0.4251 | 0.4193 | 0.0575 | 0.4518 | 0.2646 |
| 222 | 0.5962 | 0.4395 | 0.3482 | 0.4180 | 0.3913 | 0.0544 | 0.4516 | 0.2543 |
| 223 | 0.5784 | 0.4287 | 0.3563 | 0.4107 | 0.4312 | 0.0515 | 0.4516 | 0.2440 |
| 224 | 0.6544 | 0.4179 | 0.3962 | 0.4035 | 0.4331 | 0.0487 | 0.4346 | 0.2340 |
| 225 | 0.6541 | 0.4072 | 0.3954 | 0.3963 | 0.4721 | 0.0461 | 0.4522 | 0.2241 |
| 226 | 0.6514 | 0.3965 | 0.4707 | 0.3891 | 0.4937 | 0.0437 | 0.4390 | 0.2143 |
| 227 | 0.6225 | 0.3859 | 0.4709 | 0.3819 | 0.5050 | 0.0415 | 0.4389 | 0.2048 |
| 228 | 0.5678 | 0.3753 | 0.5365 | 0.3747 | 0.4888 | 0.0394 | 0.4875 | 0.1954 |
| 229 | 0.5700 | 0.3648 | 0.5293 | 0.3676 | 0.4889 | 0.0376 | 0.4873 | 0.1862 |
| 230 | 0.6129 | 0.3544 | 0.5303 | 0.3605 | 0.5214 | 0.0359 | 0.5453 | 0.1772 |
| 231 | 0.6543 | 0.3441 | 0.5251 | 0.3535 | 0.5216 | 0.0345 | 0.5453 | 0.1683 |
| 232 | 0.6605 | 0.3338 | 0.6012 | 0.3466 | 0.5232 | 0.0334 | 0.5315 | 0.1597 |
| 233 | 0.6116 | 0.3237 | 0.6009 | 0.3397 | 0.4830 | 0.0325 | 0.5774 | 0.1512 |
| 234 | 0.5925 | 0.3136 | 0.6039 | 0.3329 | 0.4779 | 0.0318 | 0.5651 | 0.1429 |
| 235 | 0.5288 | 0.3037 | 0.5941 | 0.3262 | 0.4891 | 0.0314 | 0.5665 | 0.1348 |
| 236 | 0.5137 | 0.2938 | 0.6157 | 0.3196 | 0.4895 | 0.0312 | 0.6018 | 0.1268 |
| 237 | 0.4989 | 0.2841 | 0.6772 | 0.3131 | 0.4935 | 0.0314 | 0.5410 | 0.1191 |
| 238 | 0.5972 | 0.2745 | 0.6758 | 0.3067 | 0.4971 | 0.0319 | 0.5044 | 0.1115 |
| 239 | 0.6303 | 0.2651 | 0.7080 | 0.3004 | 0.5086 | 0.0326 | 0.4785 | 0.1042 |
| 240 | 0.6303 | 0.2557 | 0.7996 | 0.2942 | 0.4969 | 0.0337 | 0.4948 | 0.0970 |
| 241 | 0.5788 | 0.2466 | 0.7893 | 0.2880 | 0.5312 | 0.0351 | 0.4983 | 0.0900 |
| 242 | 0.4967 | 0.2375 | 0.7894 | 0.2820 | 0.5312 | 0.0369 | 0.5039 | 0.0833 |
| 243 | 0.5003 | 0.2286 | 0.7913 | 0.2760 | 0.5342 | 0.0391 | 0.4771 | 0.0767 |
| 244 | 0.4752 | 0.2199 | 0.7257 | 0.2702 | 0.5347 | 0.0416 | 0.4768 | 0.0704 |
| 245 | 0.4768 | 0.2113 | 0.7495 | 0.2644 | 0.5351 | 0.0445 | 0.4678 | 0.0643 |
| 246 | 0.4786 | 0.2029 | 0.7495 | 0.2587 | 0.5351 | 0.0478 | 0.4639 | 0.0584 |
| 247 | 0.5797 | 0.1947 | 0.7356 | 0.2531 | 0.5353 | 0.0516 | 0.4909 | 0.0527 |
| 248 | 0.5906 | 0.1866 | 0.7366 | 0.2475 | 0.5114 | 0.0557 | 0.5555 | 0.0473 |
| 249 | 0.5872 | 0.1786 | 0.6544 | 0.2421 | 0.5069 | 0.0603 | 0.6071 | 0.0422 |
| 250 | 0.5552 | 0.1709 | 0.7143 | 0.2367 | 0.4690 | 0.0653 | 0.6406 | 0.0373 |
| 251 | 0.5845 | 0.1633 | 0.7213 | 0.2313 | 0.4614 | 0.0707 | 0.6580 | 0.0327 |
| 252 | 0.5863 | 0.1559 | 0.6938 | 0.2261 | 0.4613 | 0.0764 | 0.7153 | 0.0284 |
| 253 | 0.5659 | 0.1487 | 0.6283 | 0.2209 | 0.4593 | 0.0826 | 0.7425 | 0.0244 |
| 254 | 0.5343 | 0.1417 | 0.6295 | 0.2158 | 0.4487 | 0.0891 | 0.7433 | 0.0207 |
| 255 | 0.5342 | 0.1348 | 0.6025 | 0.2107 | 0.4469 | 0.0961 | 0.7371 | 0.0173 |
| 256 | 0.5898 | 0.1282 | 0.6450 | 0.2057 | 0.5160 | 0.1034 | 0.7551 | 0.0141 |
| 257 | 0.6476 | 0.1217 | 0.7249 | 0.2007 | 0.5161 | 0.1111 | 0.8038 | 0.0113 |
| 258 | 0.6358 | 0.1155 | 0.7221 | 0.1958 | 0.5566 | 0.1192 | 0.7670 | 0.0088 |
| 259 | 0.6206 | 0.1094 | 0.8005 | 0.1910 | 0.5581 | 0.1275 | 0.7070 | 0.0066 |
| 260 | 0.6710 | 0.1035 | 0.8175 | 0.1862 | 0.5488 | 0.1361 | 0.7070 | 0.0047 |
| 261 | 0.6315 | 0.0978 | 0.8178 | 0.1814 | 0.5120 | 0.1450 | 0.7284 | 0.0032 |
| 262 | 0.6427 | 0.0922 | 0.8285 | 0.1768 | 0.5041 | 0.1541 | 0.7230 | 0.0019 |
| 263 | 0.5468 | 0.0869 | 0.9017 | 0.1721 | 0.4607 | 0.1635 | 0.6696 | 0.0009 |
| 264 | 0.4456 | 0.0817 | 0.9785 | 0.1675 | 0.4607 | 0.1732 | 0.6780 | 0.0003 |
| 265 | 0.4426 | 0.0767 | 0.9926 | 0.1630 | 0.4607 | 0.1831 | 0.7239 | 0.0000 |
| 266 | 0.4411 | 0.0719 | 0.9927 | 0.1585 | 0.4935 | 0.1932 | 0.7520 | 0.0000 |
| 267 | 0.5371 | 0.0672 | 0.9913 | 0.1541 | 0.5639 | 0.2035 | 0.7674 | 0.0003 |
| 268 | 0.5381 | 0.0627 | 1.0000 | 0.1498 | 0.5255 | 0.2139 | 0.7060 | 0.0010 |
| 269 | 0.5381 | 0.0584 | 0.9998 | 0.1455 | 0.5197 | 0.2244 | 0.7072 | 0.0020 |
| 270 | 0.5666 | 0.0543 | 0.9741 | 0.1414 | 0.5690 | 0.2351 | 0.7731 | 0.0033 |
| 271 | 0.6414 | 0.0504 | 0.9972 | 0.1372 | 0.5800 | 0.2458 | 0.7719 | 0.0049 |
| 272 | 0.6657 | 0.0466 | 0.9428 | 0.1332 | 0.5683 | 0.2567 | 0.7926 | 0.0069 |
| 273 | 0.6693 | 0.0430 | 0.9333 | 0.1293 | 0.6199 | 0.2677 | 0.7925 | 0.0092 |
| 274 | 0.6697 | 0.0396 | 0.9932 | 0.1254 | 0.5407 | 0.2787 | 0.7920 | 0.0118 |
| 275 | 0.6201 | 0.0364 | 0.9782 | 0.1217 | 0.5361 | 0.2898 | 0.7917 | 0.0147 |
| 276 | 0.6226 | 0.0334 | 0.9490 | 0.1180 | 0.5621 | 0.3010 | 0.8011 | 0.0179 |
| 277 | 0.5504 | 0.0305 | 0.8894 | 0.1145 | 0.6448 | 0.3122 | 0.8256 | 0.0215 |
| 278 | 0.5503 | 0.0278 | 0.8561 | 0.1110 | 0.6461 | 0.3235 | 0.8054 | 0.0253 |
| 279 | 0.5210 | 0.0252 | 0.7945 | 0.1077 | 0.6417 | 0.3348 | 0.8054 | 0.0294 |
| 280 | 0.6316 | 0.0228 | 0.7907 | 0.1045 | 0.6966 | 0.3461 | 0.8250 | 0.0338 |
| 281 | 0.5758 | 0.0205 | 0.8625 | 0.1015 | 0.6734 | 0.3575 | 0.7697 | 0.0385 |
| 282 | 0.5494 | 0.0184 | 0.8375 | 0.0986 | 0.6738 | 0.3689 | 0.7322 | 0.0435 |
| 283 | 0.5608 | 0.0165 | 0.8376 | 0.0958 | 0.7227 | 0.3804 | 0.7583 | 0.0487 |
| 284 | 0.5233 | 0.0147 | 0.8379 | 0.0932 | 0.7228 | 0.3919 | 0.8112 | 0.0541 |
| 285 | 0.5286 | 0.0130 | 0.8268 | 0.0907 | 0.7229 | 0.4034 | 0.8525 | 0.0598 |
| 286 | 0.5628 | 0.0115 | 0.8946 | 0.0884 | 0.7229 | 0.4149 | 0.9057 | 0.0658 |
| 287 | 0.5770 | 0.0101 | 0.8905 | 0.0863 | 0.7251 | 0.4265 | 0.9057 | 0.0719 |
| 288 | 0.5182 | 0.0088 | 0.7964 | 0.0843 | 0.6902 | 0.4381 | 0.9623 | 0.0783 |
| 289 | 0.5184 | 0.0076 | 0.7990 | 0.0825 | 0.6515 | 0.4497 | 0.9592 | 0.0850 |
| 290 | 0.4396 | 0.0066 | 0.7258 | 0.0809 | 0.6501 | 0.4613 | 0.9465 | 0.0918 |
| 291 | 0.3528 | 0.0056 | 0.7250 | 0.0795 | 0.6126 | 0.4729 | 0.9437 | 0.0988 |
| 292 | 0.5083 | 0.0048 | 0.6522 | 0.0783 | 0.5814 | 0.4846 | 0.9104 | 0.1061 |
| 293 | 0.5078 | 0.0040 | 0.6598 | 0.0772 | 0.5792 | 0.4963 | 0.9648 | 0.1135 |
| 294 | 0.4662 | 0.0034 | 0.6595 | 0.0763 | 0.5783 | 0.5080 | 0.8777 | 0.1212 |
| 295 | 0.4527 | 0.0027 | 0.6462 | 0.0756 | 0.5848 | 0.5197 | 0.8598 | 0.1290 |
| 296 | 0.4537 | 0.0022 | 0.5700 | 0.0751 | 0.5839 | 0.5314 | 0.8662 | 0.1371 |
| 297 | 0.4244 | 0.0017 | 0.5624 | 0.0748 | 0.6411 | 0.5432 | 0.8070 | 0.1453 |
| 298 | 0.4435 | 0.0013 | 0.6437 | 0.0746 | 0.6390 | 0.5550 | 0.8429 | 0.1537 |
| 299 | 0.4817 | 0.0010 | 0.6642 | 0.0745 | 0.6250 | 0.5668 | 0.8658 | 0.1624 |
| 300 | 0.5740 | 0.0007 | 0.6431 | 0.0746 | 0.6542 | 0.5786 | 0.8530 | 0.1712 |
| 301 | 0.5814 | 0.0004 | 0.5817 | 0.0749 | 0.6395 | 0.5904 | 0.8527 | 0.1802 |
| 302 | 0.5063 | 0.0002 | 0.5819 | 0.0752 | 0.6453 | 0.6023 | 0.8917 | 0.1893 |
| 303 | 0.5572 | 0.0001 | 0.5528 | 0.0757 | 0.6889 | 0.6141 | 0.8868 | 0.1987 |
| 304 | 0.5567 | 0.0000 | 0.5209 | 0.0762 | 0.6860 | 0.6260 | 0.8701 | 0.2083 |
| 305 | 0.5890 | 0.0000 | 0.5264 | 0.0768 | 0.6859 | 0.6379 | 0.9346 | 0.2180 |
| 306 | 0.6714 | 0.0000 | 0.5257 | 0.0775 | 0.6859 | 0.6498 | 0.9795 | 0.2279 |
| 307 | 0.6403 | 0.0001 | 0.6056 | 0.0783 | 0.6606 | 0.6618 | 0.9116 | 0.2380 |
| 308 | 0.6347 | 0.0002 | 0.6627 | 0.0791 | 0.6606 | 0.6737 | 0.9120 | 0.2483 |
| 309 | 0.5738 | 0.0004 | 0.6390 | 0.0799 | 0.6936 | 0.6855 | 0.8730 | 0.2588 |
| 310 | 0.5746 | 0.0006 | 0.6386 | 0.0808 | 0.6933 | 0.6974 | 0.8536 | 0.2695 |
| 311 | 0.5272 | 0.0008 | 0.6507 | 0.0818 | 0.7073 | 0.7092 | 0.7872 | 0.2803 |
| 312 | 0.5168 | 0.0010 | 0.5848 | 0.0827 | 0.7173 | 0.7210 | 0.7821 | 0.2913 |
| 313 | 0.5511 | 0.0013 | 0.6086 | 0.0837 | 0.7172 | 0.7327 | 0.7967 | 0.3025 |
| 314 | 0.5513 | 0.0016 | 0.5115 | 0.0847 | 0.7646 | 0.7442 | 0.8020 | 0.3138 |
| 315 | 0.5284 | 0.0019 | 0.4962 | 0.0857 | 0.8233 | 0.7557 | 0.8615 | 0.3254 |
| 316 | 0.6149 | 0.0023 | 0.6030 | 0.0867 | 0.8541 | 0.7671 | 0.8000 | 0.3370 |
| 317 | 0.6032 | 0.0026 | 0.6038 | 0.0878 | 0.8563 | 0.7783 | 0.7729 | 0.3489 |
| 318 | 0.6103 | 0.0030 | 0.5917 | 0.0888 | 0.8669 | 0.7894 | 0.7812 | 0.3608 |
| 319 | 0.5993 | 0.0034 | 0.6029 | 0.0899 | 0.8974 | 0.8003 | 0.7712 | 0.3730 |
| 320 | 0.5969 | 0.0038 | 0.6761 | 0.0911 | 0.8677 | 0.8110 | 0.7455 | 0.3852 |
| 321 | 0.5396 | 0.0042 | 0.5962 | 0.0922 | 0.9360 | 0.8215 | 0.6944 | 0.3976 |
| 322 | 0.4476 | 0.0047 | 0.6857 | 0.0934 | 0.9286 | 0.8317 | 0.7090 | 0.4100 |
| 323 | 0.4141 | 0.0052 | 0.6072 | 0.0946 | 0.9130 | 0.8416 | 0.7059 | 0.4226 |
| 324 | 0.4038 | 0.0058 | 0.5491 | 0.0959 | 0.9207 | 0.8513 | 0.7435 | 0.4353 |
| 325 | 0.4369 | 0.0064 | 0.5444 | 0.0972 | 0.9574 | 0.8606 | 0.7293 | 0.4480 |
| 326 | 0.4698 | 0.0070 | 0.5431 | 0.0986 | 1.0000 | 0.8696 | 0.7289 | 0.4608 |
| 327 | 0.5458 | 0.0077 | 0.4834 | 0.1000 | 0.9672 | 0.8784 | 0.7137 | 0.4736 |
| 328 | 0.5859 | 0.0085 | 0.4749 | 0.1016 | 0.9306 | 0.8867 | 0.6864 | 0.4865 |
| 329 | 0.6324 | 0.0093 | 0.4603 | 0.1032 | 0.9605 | 0.8947 | 0.6271 | 0.4993 |
| 330 | 0.6369 | 0.0101 | 0.4579 | 0.1048 | 0.9276 | 0.9024 | 0.6051 | 0.5122 |
| 331 | 0.6399 | 0.0110 | 0.4818 | 0.1066 | 0.8888 | 0.9097 | 0.6136 | 0.5250 |
| 332 | 0.6104 | 0.0120 | 0.4732 | 0.1084 | 0.8865 | 0.9167 | 0.6153 | 0.5377 |
| 333 | 0.6104 | 0.0130 | 0.5110 | 0.1103 | 0.8922 | 0.9233 | 0.6152 | 0.5504 |
| 334 | 0.6436 | 0.0141 | 0.5195 | 0.1123 | 0.8348 | 0.9295 | 0.5341 | 0.5631 |
| 335 | 0.4788 | 0.0153 | 0.5712 | 0.1143 | 0.8744 | 0.9353 | 0.5353 | 0.5756 |
| 336 | 0.4141 | 0.0165 | 0.6681 | 0.1165 | 0.8846 | 0.9408 | 0.5708 | 0.5880 |
| 337 | 0.4129 | 0.0178 | 0.7511 | 0.1188 | 0.8698 | 0.9460 | 0.5204 | 0.6004 |
| 338 | 0.4123 | 0.0191 | 0.6785 | 0.1211 | 0.8699 | 0.9508 | 0.5001 | 0.6126 |
| 339 | 0.3731 | 0.0205 | 0.7212 | 0.1236 | 0.8743 | 0.9553 | 0.4997 | 0.6247 |
| 340 | 0.4240 | 0.0220 | 0.7171 | 0.1262 | 0.8042 | 0.9596 | 0.4879 | 0.6367 |
| 341 | 0.4422 | 0.0235 | 0.7914 | 0.1289 | 0.7481 | 0.9635 | 0.5510 | 0.6486 |
| 342 | 0.4423 | 0.0251 | 0.7914 | 0.1318 | 0.7027 | 0.9672 | 0.5280 | 0.6603 |
| 343 | 0.4401 | 0.0267 | 0.7938 | 0.1348 | 0.7259 | 0.9706 | 0.5737 | 0.6719 |
| 344 | 0.3686 | 0.0284 | 0.7145 | 0.1379 | 0.6600 | 0.9737 | 0.5900 | 0.6834 |
| 345 | 0.3560 | 0.0302 | 0.7148 | 0.1411 | 0.7530 | 0.9766 | 0.5616 | 0.6947 |
| 346 | 0.3066 | 0.0320 | 0.7210 | 0.1446 | 0.7512 | 0.9793 | 0.5689 | 0.7058 |
| 347 | 0.3066 | 0.0338 | 0.7199 | 0.1482 | 0.6994 | 0.9818 | 0.5426 | 0.7168 |
| 348 | 0.3511 | 0.0357 | 0.7201 | 0.1519 | 0.6991 | 0.9841 | 0.5069 | 0.7276 |
| 349 | 0.3529 | 0.0377 | 0.7142 | 0.1559 | 0.6974 | 0.9862 | 0.4411 | 0.7382 |
| 350 | 0.3531 | 0.0397 | 0.7149 | 0.1600 | 0.6971 | 0.9882 | 0.3876 | 0.7487 |
| 351 | 0.3569 | 0.0418 | 0.7128 | 0.1643 | 0.6671 | 0.9900 | 0.4452 | 0.7590 |
| 352 | 0.4035 | 0.0439 | 0.7924 | 0.1688 | 0.7020 | 0.9916 | 0.3591 | 0.7691 |
| 353 | 0.4008 | 0.0461 | 0.7920 | 0.1734 | 0.6992 | 0.9931 | 0.4060 | 0.7791 |
| 354 | 0.4809 | 0.0484 | 0.7813 | 0.1783 | 0.6780 | 0.9945 | 0.4103 | 0.7889 |
| 355 | 0.4987 | 0.0506 | 0.7821 | 0.1833 | 0.6796 | 0.9957 | 0.4337 | 0.7985 |
| 356 | 0.3946 | 0.0530 | 0.7893 | 0.1885 | 0.7534 | 0.9968 | 0.4352 | 0.8079 |
| 357 | 0.3957 | 0.0554 | 0.7870 | 0.1939 | 0.7678 | 0.9977 | 0.4460 | 0.8171 |
| 358 | 0.3518 | 0.0578 | 0.8552 | 0.1995 | 0.7853 | 0.9985 | 0.4750 | 0.8262 |
| 359 | 0.3155 | 0.0603 | 0.8729 | 0.2053 | 0.7788 | 0.9992 | 0.5044 | 0.8351 |
| 360 | 0.3155 | 0.0628 | 0.8708 | 0.2113 | 0.7797 | 0.9996 | 0.5472 | 0.8438 |
| 361 | 0.3036 | 0.0654 | 0.9645 | 0.2174 | 0.7221 | 0.9999 | 0.5450 | 0.8523 |
| 362 | 0.2733 | 0.0680 | 0.8838 | 0.2237 | 0.7242 | 1.0000 | 0.4959 | 0.8607 |
| 363 | 0.2988 | 0.0707 | 0.8721 | 0.2302 | 0.7674 | 0.9999 | 0.5091 | 0.8688 |
| 364 | 0.2225 | 0.0734 | 0.9494 | 0.2369 | 0.7689 | 0.9996 | 0.4925 | 0.8768 |
| 365 | 0.2368 | 0.0762 | 0.9077 | 0.2438 | 0.7839 | 0.9991 | 0.4928 | 0.8846 |
| 366 | 0.2516 | 0.0790 | 0.9089 | 0.2508 | 0.7932 | 0.9983 | 0.4538 | 0.8922 |
| 367 | 0.1794 | 0.0818 | 0.9089 | 0.2579 | 0.7493 | 0.9973 | 0.4540 | 0.8996 |
| 368 | 0.2265 | 0.0847 | 0.8610 | 0.2653 | 0.7523 | 0.9961 | 0.4540 | 0.9068 |
| 369 | 0.2337 | 0.0876 | 0.8792 | 0.2727 | 0.7687 | 0.9946 | 0.3888 | 0.9137 |
| 370 | 0.2339 | 0.0905 | 0.8798 | 0.2804 | 0.7508 | 0.9927 | 0.3376 | 0.9205 |
| 371 | 0.2649 | 0.0935 | 0.7996 | 0.2882 | 0.7761 | 0.9906 | 0.3755 | 0.9271 |
| 372 | 0.3073 | 0.0965 | 0.7471 | 0.2961 | 0.8145 | 0.9881 | 0.4023 | 0.9334 |
| 373 | 0.4125 | 0.0994 | 0.7463 | 0.3043 | 0.7575 | 0.9853 | 0.4517 | 0.9395 |
| 374 | 0.3366 | 0.1024 | 0.7466 | 0.3125 | 0.7578 | 0.9821 | 0.4752 | 0.9453 |
| 375 | 0.4218 | 0.1054 | 0.7945 | 0.3209 | 0.7440 | 0.9785 | 0.5035 | 0.9509 |
| 376 | 0.3949 | 0.1084 | 0.7946 | 0.3295 | 0.7338 | 0.9746 | 0.4446 | 0.9563 |
| 377 | 0.3640 | 0.1114 | 0.7462 | 0.3381 | 0.6949 | 0.9702 | 0.3520 | 0.9613 |
| 378 | 0.3638 | 0.1144 | 0.7828 | 0.3470 | 0.6269 | 0.9655 | 0.2980 | 0.9661 |
| 379 | 0.3487 | 0.1174 | 0.8546 | 0.3560 | 0.5602 | 0.9604 | 0.2374 | 0.9706 |
| 380 | 0.2884 | 0.1204 | 0.7651 | 0.3651 | 0.5290 | 0.9549 | 0.2415 | 0.9748 |
| 381 | 0.3332 | 0.1233 | 0.7650 | 0.3743 | 0.5290 | 0.9490 | 0.2366 | 0.9787 |
| 382 | 0.3528 | 0.1263 | 0.7750 | 0.3837 | 0.5658 | 0.9427 | 0.2345 | 0.9822 |
| 383 | 0.3627 | 0.1292 | 0.7692 | 0.3932 | 0.5576 | 0.9359 | 0.2502 | 0.9855 |
| 384 | 0.4325 | 0.1322 | 0.6964 | 0.4029 | 0.5159 | 0.9288 | 0.2831 | 0.9884 |
| 385 | 0.4326 | 0.1351 | 0.6959 | 0.4126 | 0.4671 | 0.9213 | 0.2875 | 0.9911 |
| 386 | 0.5077 | 0.1380 | 0.6742 | 0.4225 | 0.4424 | 0.9134 | 0.2875 | 0.9934 |
| 387 | 0.5019 | 0.1409 | 0.6742 | 0.4326 | 0.4578 | 0.9052 | 0.3486 | 0.9953 |
| 388 | 0.4709 | 0.1438 | 0.7154 | 0.4428 | 0.4565 | 0.8967 | 0.3111 | 0.9969 |
| 389 | 0.4695 | 0.1467 | 0.7565 | 0.4531 | 0.4473 | 0.8879 | 0.3023 | 0.9982 |
| 390 | 0.4249 | 0.1495 | 0.7558 | 0.4635 | 0.4639 | 0.8788 | 0.3034 | 0.9991 |
| 391 | 0.3757 | 0.1524 | 0.7331 | 0.4740 | 0.5041 | 0.8693 | 0.3437 | 0.9997 |
| 392 | 0.3727 | 0.1552 | 0.6650 | 0.4846 | 0.5667 | 0.8596 | 0.3895 | 1.0000 |
| 393 | 0.3274 | 0.1580 | 0.6902 | 0.4954 | 0.5367 | 0.8497 | 0.4028 | 0.9999 |
| 394 | 0.3962 | 0.1608 | 0.6925 | 0.5062 | 0.5427 | 0.8395 | 0.4005 | 0.9994 |
| 395 | 0.4088 | 0.1636 | 0.6685 | 0.5172 | 0.5109 | 0.8291 | 0.3989 | 0.9986 |
| 396 | 0.4316 | 0.1664 | 0.6541 | 0.5282 | 0.4753 | 0.8186 | 0.3761 | 0.9975 |
| 397 | 0.4314 | 0.1692 | 0.6158 | 0.5393 | 0.4504 | 0.8079 | 0.4275 | 0.9960 |
| 398 | 0.3683 | 0.1719 | 0.6161 | 0.5505 | 0.4781 | 0.7971 | 0.4436 | 0.9942 |
| 399 | 0.4426 | 0.1746 | 0.5583 | 0.5616 | 0.4682 | 0.7861 | 0.4415 | 0.9920 |
| 400 | 0.4799 | 0.1773 | 0.4722 | 0.5729 | 0.5245 | 0.7751 | 0.4311 | 0.9895 |
| 401 | 0.4832 | 0.1800 | 0.4731 | 0.5841 | 0.5114 | 0.7640 | 0.4588 | 0.9867 |
| 402 | 0.4840 | 0.1826 | 0.4736 | 0.5954 | 0.5084 | 0.7528 | 0.5083 | 0.9835 |
| 403 | 0.4622 | 0.1852 | 0.3984 | 0.6066 | 0.5414 | 0.7416 | 0.5088 | 0.9801 |
| 404 | 0.3956 | 0.1878 | 0.3475 | 0.6179 | 0.6131 | 0.7303 | 0.5748 | 0.9763 |
| 405 | 0.3997 | 0.1903 | 0.2727 | 0.6290 | 0.5858 | 0.7189 | 0.4984 | 0.9722 |
| 406 | 0.3997 | 0.1928 | 0.2673 | 0.6401 | 0.6313 | 0.7075 | 0.4984 | 0.9679 |
| 407 | 0.3890 | 0.1952 | 0.2673 | 0.6511 | 0.6810 | 0.6961 | 0.4790 | 0.9633 |
| 408 | 0.3905 | 0.1976 | 0.2676 | 0.6620 | 0.6759 | 0.6846 | 0.5158 | 0.9584 |
| 409 | 0.4037 | 0.1999 | 0.2676 | 0.6728 | 0.6166 | 0.6731 | 0.5524 | 0.9532 |
| 410 | 0.4731 | 0.2021 | 0.2870 | 0.6836 | 0.6335 | 0.6616 | 0.5967 | 0.9478 |
| 411 | 0.4397 | 0.2043 | 0.2880 | 0.6942 | 0.6365 | 0.6500 | 0.6121 | 0.9421 |
| 412 | 0.4173 | 0.2065 | 0.2880 | 0.7047 | 0.6265 | 0.6385 | 0.6367 | 0.9362 |
| 413 | 0.4189 | 0.2085 | 0.2925 | 0.7151 | 0.6278 | 0.6269 | 0.6382 | 0.9300 |
| 414 | 0.4183 | 0.2105 | 0.2926 | 0.7254 | 0.6368 | 0.6153 | 0.6058 | 0.9236 |
| 415 | 0.4992 | 0.2125 | 0.3199 | 0.7356 | 0.6548 | 0.6037 | 0.5585 | 0.9170 |
| 416 | 0.4927 | 0.2144 | 0.2953 | 0.7457 | 0.6864 | 0.5921 | 0.5886 | 0.9102 |
| 417 | 0.5016 | 0.2162 | 0.3044 | 0.7556 | 0.7169 | 0.5805 | 0.4997 | 0.9032 |
| 418 | 0.5005 | 0.2179 | 0.2733 | 0.7654 | 0.7551 | 0.5689 | 0.4945 | 0.8960 |
| 419 | 0.4560 | 0.2196 | 0.2125 | 0.7751 | 0.7546 | 0.5573 | 0.4566 | 0.8886 |
| 420 | 0.4438 | 0.2212 | 0.2121 | 0.7846 | 0.6807 | 0.5457 | 0.4917 | 0.8809 |
| 421 | 0.4416 | 0.2228 | 0.2253 | 0.7940 | 0.6946 | 0.5341 | 0.4305 | 0.8731 |
| 422 | 0.4202 | 0.2243 | 0.1888 | 0.8032 | 0.6779 | 0.5224 | 0.4565 | 0.8651 |
| 423 | 0.3738 | 0.2257 | 0.2667 | 0.8122 | 0.6774 | 0.5107 | 0.4649 | 0.8569 |
| 424 | 0.3698 | 0.2271 | 0.2726 | 0.8211 | 0.7006 | 0.4991 | 0.4156 | 0.8485 |
| 425 | 0.4310 | 0.2284 | 0.1868 | 0.8297 | 0.7059 | 0.4874 | 0.4569 | 0.8399 |
| 426 | 0.4612 | 0.2296 | 0.1869 | 0.8382 | 0.7060 | 0.4757 | 0.4702 | 0.8311 |
| 427 | 0.4413 | 0.2308 | 0.1988 | 0.8465 | 0.6729 | 0.4641 | 0.4531 | 0.8221 |
| 428 | 0.4689 | 0.2318 | 0.1215 | 0.8546 | 0.6792 | 0.4524 | 0.4771 | 0.8130 |
| 429 | 0.4358 | 0.2329 | 0.1631 | 0.8625 | 0.7260 | 0.4407 | 0.4997 | 0.8037 |
| 430 | 0.4095 | 0.2338 | 0.2444 | 0.8702 | 0.6933 | 0.4290 | 0.5610 | 0.7941 |
| 431 | 0.4100 | 0.2347 | 0.2445 | 0.8776 | 0.6659 | 0.4173 | 0.5416 | 0.7845 |
| 432 | 0.3513 | 0.2355 | 0.2446 | 0.8849 | 0.6918 | 0.4056 | 0.5106 | 0.7746 |
| 433 | 0.3540 | 0.2362 | 0.3046 | 0.8920 | 0.6943 | 0.3939 | 0.5733 | 0.7646 |
| 434 | 0.3828 | 0.2368 | 0.3081 | 0.8988 | 0.6813 | 0.3822 | 0.5650 | 0.7544 |
| 435 | 0.4134 | 0.2374 | 0.3080 | 0.9054 | 0.6808 | 0.3706 | 0.6281 | 0.7441 |
| 436 | 0.3902 | 0.2379 | 0.3072 | 0.9119 | 0.6186 | 0.3590 | 0.6011 | 0.7337 |
| 437 | 0.3556 | 0.2383 | 0.3079 | 0.9181 | 0.6401 | 0.3473 | 0.4943 | 0.7232 |
| 438 | 0.4594 | 0.2387 | 0.3080 | 0.9240 | 0.6398 | 0.3358 | 0.4888 | 0.7125 |
| 439 | 0.4114 | 0.2389 | 0.3096 | 0.9298 | 0.6397 | 0.3243 | 0.4660 | 0.7018 |
| 440 | 0.5124 | 0.2392 | 0.3745 | 0.9354 | 0.6528 | 0.3129 | 0.4663 | 0.6909 |
| 441 | 0.5123 | 0.2393 | 0.3988 | 0.9407 | 0.6917 | 0.3015 | 0.4918 | 0.6800 |
| 442 | 0.5471 | 0.2394 | 0.4029 | 0.9458 | 0.6316 | 0.2903 | 0.5561 | 0.6690 |
| 443 | 0.5618 | 0.2395 | 0.3396 | 0.9508 | 0.6354 | 0.2792 | 0.5557 | 0.6580 |
| 444 | 0.5324 | 0.2394 | 0.4048 | 0.9555 | 0.6299 | 0.2682 | 0.5556 | 0.6468 |
| 445 | 0.4901 | 0.2393 | 0.4753 | 0.9600 | 0.6276 | 0.2573 | 0.5606 | 0.6356 |
| 446 | 0.3906 | 0.2392 | 0.5017 | 0.9642 | 0.5514 | 0.2467 | 0.5023 | 0.6244 |
| 447 | 0.3529 | 0.2390 | 0.4950 | 0.9683 | 0.5309 | 0.2362 | 0.5027 | 0.6131 |
| 448 | 0.2816 | 0.2387 | 0.4891 | 0.9721 | 0.5340 | 0.2259 | 0.4955 | 0.6018 |
| 449 | 0.2816 | 0.2383 | 0.4895 | 0.9757 | 0.5349 | 0.2158 | 0.4939 | 0.5904 |
| 450 | 0.2849 | 0.2379 | 0.4246 | 0.9791 | 0.5264 | 0.2060 | 0.5057 | 0.5791 |
| 451 | 0.2773 | 0.2375 | 0.4245 | 0.9822 | 0.4891 | 0.1965 | 0.5070 | 0.5677 |
| 452 | 0.3199 | 0.2369 | 0.4245 | 0.9851 | 0.4589 | 0.1872 | 0.5555 | 0.5564 |
| 453 | 0.4235 | 0.2364 | 0.3882 | 0.9877 | 0.4748 | 0.1783 | 0.5646 | 0.5451 |
| 454 | 0.5095 | 0.2357 | 0.4571 | 0.9901 | 0.4501 | 0.1697 | 0.5629 | 0.5338 |
| 455 | 0.5240 | 0.2350 | 0.4627 | 0.9923 | 0.4265 | 0.1613 | 0.5669 | 0.5227 |
| 456 | 0.5524 | 0.2343 | 0.4626 | 0.9941 | 0.3989 | 0.1533 | 0.5484 | 0.5115 |
| 457 | 0.5838 | 0.2334 | 0.4381 | 0.9958 | 0.3963 | 0.1457 | 0.5849 | 0.5005 |
| 458 | 0.5121 | 0.2325 | 0.4378 | 0.9971 | 0.3892 | 0.1383 | 0.5872 | 0.4896 |
| 459 | 0.5189 | 0.2316 | 0.4393 | 0.9982 | 0.3893 | 0.1313 | 0.5945 | 0.4789 |
| 460 | 0.5262 | 0.2306 | 0.4535 | 0.9991 | 0.3959 | 0.1247 | 0.6172 | 0.4683 |
| 461 | 0.5500 | 0.2295 | 0.4537 | 0.9996 | 0.4144 | 0.1184 | 0.5685 | 0.4579 |
| 462 | 0.5421 | 0.2284 | 0.4535 | 0.9999 | 0.4264 | 0.1124 | 0.5690 | 0.4476 |
| 463 | 0.5433 | 0.2272 | 0.4541 | 1.0000 | 0.3999 | 0.1068 | 0.5817 | 0.4375 |
| 464 | 0.5834 | 0.2259 | 0.4063 | 0.9998 | 0.3450 | 0.1016 | 0.5378 | 0.4276 |
| 465 | 0.6364 | 0.2246 | 0.3180 | 0.9993 | 0.3583 | 0.0966 | 0.5102 | 0.4179 |
| 466 | 0.6642 | 0.2232 | 0.3164 | 0.9985 | 0.2943 | 0.0919 | 0.4607 | 0.4084 |
| 467 | 0.7165 | 0.2218 | 0.3397 | 0.9974 | 0.3414 | 0.0874 | 0.4733 | 0.3991 |
| 468 | 0.7371 | 0.2203 | 0.3985 | 0.9961 | 0.3419 | 0.0833 | 0.4088 | 0.3900 |
| 469 | 0.7359 | 0.2187 | 0.3982 | 0.9944 | 0.2733 | 0.0794 | 0.4319 | 0.3811 |
| 470 | 0.7297 | 0.2171 | 0.4023 | 0.9925 | 0.2724 | 0.0757 | 0.4320 | 0.3724 |
| 471 | 0.7354 | 0.2154 | 0.3994 | 0.9903 | 0.2046 | 0.0723 | 0.4057 | 0.3639 |
| 472 | 0.7353 | 0.2136 | 0.4600 | 0.9877 | 0.2208 | 0.0691 | 0.3526 | 0.3557 |
| 473 | 0.7360 | 0.2118 | 0.5452 | 0.9850 | 0.2363 | 0.0661 | 0.3455 | 0.3476 |
| 474 | 0.7249 | 0.2100 | 0.5445 | 0.9819 | 0.2215 | 0.0632 | 0.3011 | 0.3398 |
| 475 | 0.8068 | 0.2081 | 0.5441 | 0.9785 | 0.2212 | 0.0605 | 0.2864 | 0.3323 |
| 476 | 0.7849 | 0.2061 | 0.5399 | 0.9748 | 0.2538 | 0.0580 | 0.2619 | 0.3249 |
| 477 | 0.7741 | 0.2041 | 0.5415 | 0.9708 | 0.2541 | 0.0555 | 0.3137 | 0.3178 |
| 478 | 0.7676 | 0.2020 | 0.5415 | 0.9666 | 0.2639 | 0.0532 | 0.3639 | 0.3109 |
| 479 | 0.6868 | 0.1999 | 0.5193 | 0.9620 | 0.2773 | 0.0510 | 0.3536 | 0.3042 |
| 480 | 0.7043 | 0.1977 | 0.5208 | 0.9573 | 0.2683 | 0.0488 | 0.3549 | 0.2977 |
| 481 | 0.7737 | 0.1955 | 0.5116 | 0.9522 | 0.2036 | 0.0467 | 0.3822 | 0.2914 |
| 482 | 0.7493 | 0.1932 | 0.5478 | 0.9469 | 0.2217 | 0.0448 | 0.3829 | 0.2853 |
| 483 | 0.7939 | 0.1909 | 0.6843 | 0.9413 | 0.2153 | 0.0428 | 0.3899 | 0.2794 |
| 484 | 0.8531 | 0.1885 | 0.6847 | 0.9355 | 0.2511 | 0.0409 | 0.3381 | 0.2737 |
| 485 | 0.8246 | 0.1861 | 0.6932 | 0.9295 | 0.2251 | 0.0391 | 0.3325 | 0.2682 |
| 486 | 0.8899 | 0.1836 | 0.7413 | 0.9233 | 0.2251 | 0.0373 | 0.3103 | 0.2629 |
| 487 | 0.9453 | 0.1811 | 0.6643 | 0.9169 | 0.2255 | 0.0356 | 0.2904 | 0.2577 |
| 488 | 0.9516 | 0.1785 | 0.7195 | 0.9103 | 0.1996 | 0.0339 | 0.2905 | 0.2527 |
| 489 | 0.9406 | 0.1759 | 0.7195 | 0.9035 | 0.1708 | 0.0323 | 0.2492 | 0.2479 |
| 490 | 0.8970 | 0.1733 | 0.7193 | 0.8965 | 0.1545 | 0.0307 | 0.2492 | 0.2433 |
| 491 | 0.9263 | 0.1706 | 0.7082 | 0.8893 | 0.1935 | 0.0291 | 0.2314 | 0.2389 |
| 492 | 0.8975 | 0.1679 | 0.7087 | 0.8820 | 0.1566 | 0.0276 | 0.2314 | 0.2347 |
| 493 | 0.9503 | 0.1651 | 0.7065 | 0.8745 | 0.1435 | 0.0261 | 0.2089 | 0.2306 |
| 494 | 0.9455 | 0.1623 | 0.6250 | 0.8669 | 0.1608 | 0.0246 | 0.1576 | 0.2267 |
| 495 | 0.9593 | 0.1595 | 0.6245 | 0.8591 | 0.2134 | 0.0231 | 0.1768 | 0.2230 |
| 496 | 1.0000 | 0.1566 | 0.7027 | 0.8511 | 0.2090 | 0.0217 | 0.2334 | 0.2196 |
| 497 | 0.9831 | 0.1537 | 0.6471 | 0.8430 | 0.1845 | 0.0203 | 0.1716 | 0.2163 |
| 498 | 0.9543 | 0.1508 | 0.6464 | 0.8347 | 0.2500 | 0.0189 | 0.1820 | 0.2132 |
| 499 | 0.8886 | 0.1479 | 0.6470 | 0.8262 | 0.2212 | 0.0176 | 0.0603 | 0.2104 |
| 500 | 0.8802 | 0.1449 | 0.6458 | 0.8177 | 0.2761 | 0.0163 | — | — |
| 501 | 0.8734 | 0.1420 | 0.6457 | 0.8090 | 0.2783 | 0.0150 | — | — |
| 502 | 0.8687 | 0.1390 | 0.7187 | 0.8001 | 0.2293 | 0.0137 | — | — |
| 503 | 0.7773 | 0.1361 | 0.7334 | 0.7912 | 0.2293 | 0.0125 | — | — |
| 504 | 0.7093 | 0.1331 | 0.7025 | 0.7821 | 0.2035 | 0.0113 | — | — |
| 505 | 0.6999 | 0.1302 | 0.7476 | 0.7729 | 0.2034 | 0.0102 | — | — |
| 506 | 0.6265 | 0.1272 | 0.7460 | 0.7635 | 0.2451 | 0.0091 | — | — |
| 507 | 0.6373 | 0.1243 | 0.7464 | 0.7540 | 0.2492 | 0.0081 | — | — |
| 508 | 0.7348 | 0.1214 | 0.7003 | 0.7444 | 0.2251 | 0.0070 | — | — |
| 509 | 0.7166 | 0.1185 | 0.6236 | 0.7347 | 0.2258 | 0.0061 | — | — |
| 510 | 0.7744 | 0.1156 | 0.5962 | 0.7249 | 0.2626 | 0.0052 | — | — |
| 511 | 0.8133 | 0.1127 | 0.5930 | 0.7149 | 0.2564 | 0.0043 | — | — |
| 512 | 0.8484 | 0.1099 | 0.5990 | 0.7048 | 0.2343 | 0.0036 | — | — |
| 513 | 0.8365 | 0.1070 | 0.5983 | 0.6946 | 0.2139 | 0.0028 | — | — |
| 514 | 0.8363 | 0.1042 | 0.5985 | 0.6842 | 0.2058 | 0.0022 | — | — |
| 515 | 0.8023 | 0.1014 | 0.5986 | 0.6737 | 0.2240 | 0.0016 | — | — |
| 516 | 0.8377 | 0.0987 | 0.6246 | 0.6631 | 0.2358 | 0.0011 | — | — |
| 517 | 0.6617 | 0.0959 | 0.7026 | 0.6524 | 0.2229 | 0.0007 | — | — |
| 518 | 0.5743 | 0.0932 | 0.6251 | 0.6416 | 0.1783 | 0.0004 | — | — |
| 519 | 0.5890 | 0.0905 | 0.6307 | 0.6307 | 0.1845 | 0.0001 | — | — |
| 520 | 0.5640 | 0.0878 | 0.6483 | 0.6196 | 0.1862 | 0.0000 | — | — |
| 521 | 0.5304 | 0.0852 | 0.6466 | 0.6085 | 0.1779 | 0.0000 | — | — |
| 522 | 0.4552 | 0.0825 | 0.6462 | 0.5972 | 0.1495 | 0.0001 | — | — |
| 523 | 0.4346 | 0.0799 | 0.6462 | 0.5858 | 0.1495 | 0.0002 | — | — |
| 524 | 0.5199 | 0.0773 | 0.6452 | 0.5744 | 0.1704 | 0.0005 | — | — |
| 525 | 0.4707 | 0.0747 | 0.6457 | 0.5630 | 0.1479 | 0.0009 | — | — |
| 526 | 0.5090 | 0.0721 | 0.6455 | 0.5514 | 0.1133 | 0.0014 | — | — |
| 527 | 0.4558 | 0.0695 | 0.6307 | 0.5399 | 0.1484 | 0.0019 | — | — |
| 528 | 0.4214 | 0.0670 | 0.6785 | 0.5283 | 0.1445 | 0.0026 | — | — |
| 529 | 0.3662 | 0.0645 | 0.6840 | 0.5167 | 0.0799 | 0.0034 | — | — |
| 530 | 0.3437 | 0.0620 | 0.6855 | 0.5051 | 0.1370 | 0.0043 | — | — |
| 531 | 0.3415 | 0.0596 | 0.6654 | 0.4935 | 0.1034 | 0.0053 | — | — |
| 532 | 0.2898 | 0.0572 | 0.6616 | 0.4820 | 0.1067 | 0.0063 | — | — |
| 533 | 0.2527 | 0.0549 | 0.6548 | 0.4706 | 0.1075 | 0.0075 | — | — |
| 534 | 0.2570 | 0.0526 | 0.6561 | 0.4593 | 0.1084 | 0.0088 | — | — |
| 535 | 0.2658 | 0.0503 | 0.6590 | 0.4480 | 0.0945 | 0.0102 | — | — |
| 536 | 0.2662 | 0.0482 | 0.5943 | 0.4368 | 0.0942 | 0.0117 | — | — |
| 537 | 0.2662 | 0.0460 | 0.5085 | 0.4258 | 0.0682 | 0.0132 | — | — |
| 538 | 0.2754 | 0.0440 | 0.5163 | 0.4148 | 0.0725 | 0.0149 | — | — |
| 539 | 0.2617 | 0.0420 | 0.5166 | 0.4039 | 0.1247 | 0.0167 | — | — |
| 540 | 0.2713 | 0.0400 | 0.5204 | 0.3931 | 0.0973 | 0.0187 | — | — |
| 541 | 0.2638 | 0.0381 | 0.5195 | 0.3825 | 0.0969 | 0.0207 | — | — |
| 542 | 0.2707 | 0.0363 | 0.5195 | 0.3720 | 0.0667 | 0.0228 | — | — |
| 543 | 0.2790 | 0.0345 | 0.5171 | 0.3616 | 0.0628 | 0.0251 | — | — |
| 544 | 0.2354 | 0.0328 | 0.5462 | 0.3513 | 0.0263 | 0.0275 | — | — |
| 545 | 0.1620 | 0.0311 | 0.5070 | 0.3411 | 0.0635 | 0.0300 | — | — |
| 546 | 0.1412 | 0.0295 | 0.5126 | 0.3311 | 0.0288 | 0.0326 | — | — |
| 547 | 0.1404 | 0.0280 | 0.4350 | 0.3213 | 0.0353 | 0.0353 | — | — |
| 548 | 0.0648 | 0.0265 | 0.4213 | 0.3116 | 0.0000 | 0.0382 | — | — |
| 549 | 0.0983 | 0.0251 | 0.4752 | 0.3021 | 0.0600 | 0.0412 | — | — |
| 550 | 0.0978 | 0.0237 | 0.4777 | 0.2927 | 0.0731 | 0.0443 | — | — |
| 551 | 0.0859 | 0.0224 | 0.4768 | 0.2836 | 0.0931 | 0.0476 | — | — |
| 552 | 0.0838 | 0.0212 | 0.4191 | 0.2745 | 0.1338 | 0.0510 | — | — |
| 553 | 0.0737 | 0.0200 | 0.4860 | 0.2657 | 0.2258 | 0.0545 | — | — |
| 554 | 0.1174 | 0.0189 | 0.4142 | 0.2571 | 0.2419 | 0.0582 | — | — |
| 555 | 0.0904 | 0.0178 | 0.4143 | 0.2487 | 0.2047 | 0.0619 | — | — |
| 556 | 0.0196 | 0.0168 | 0.4121 | 0.2404 | 0.2044 | 0.0659 | — | — |
| 557 | 0.0000 | 0.0159 | 0.4140 | 0.2324 | 0.1897 | 0.0699 | — | — |
| 558 | 0.0023 | 0.0150 | 0.3800 | 0.2245 | 0.1801 | 0.0741 | — | — |
| 559 | 0.0668 | 0.0141 | 0.3933 | 0.2169 | 0.1541 | 0.0784 | — | — |
| 560 | 0.0673 | 0.0133 | 0.3450 | 0.2095 | 0.1314 | 0.0829 | — | — |
| 561 | 0.0821 | 0.0126 | 0.3094 | 0.2023 | 0.1370 | 0.0875 | — | — |
| 562 | 0.0822 | 0.0119 | 0.3758 | 0.1953 | 0.1021 | 0.0922 | — | — |
| 563 | 0.1156 | 0.0113 | 0.3490 | 0.1885 | 0.1810 | 0.0971 | — | — |
| 564 | 0.1105 | 0.0107 | 0.3594 | 0.1820 | 0.1498 | 0.1021 | — | — |
| 565 | 0.1055 | 0.0102 | 0.3585 | 0.1756 | 0.1495 | 0.1072 | — | — |
| 566 | 0.0579 | 0.0097 | 0.2849 | 0.1695 | 0.2031 | 0.1124 | — | — |
| 567 | 0.1582 | 0.0093 | 0.2235 | 0.1635 | 0.2384 | 0.1178 | — | — |
| 568 | 0.1516 | 0.0089 | 0.1630 | 0.1578 | 0.2467 | 0.1233 | — | — |
| 569 | 0.1871 | 0.0086 | 0.0519 | 0.1523 | 0.2647 | 0.1290 | — | — |
| 570 | 0.2258 | 0.0083 | 0.0568 | 0.1469 | 0.3124 | 0.1347 | — | — |
| 571 | 0.1699 | 0.0081 | 0.1308 | 0.1417 | 0.3464 | 0.1406 | — | — |
| 572 | 0.0752 | 0.0079 | 0.1308 | 0.1367 | 0.3589 | 0.1467 | — | — |
| 573 | 0.1573 | 0.0077 | 0.1369 | 0.1319 | 0.3802 | 0.1528 | — | — |
| 574 | 0.2609 | 0.0076 | 0.1264 | 0.1273 | 0.3714 | 0.1590 | — | — |
| 575 | 0.2616 | 0.0075 | 0.1311 | 0.1228 | 0.3776 | 0.1654 | — | — |
| 576 | 0.2349 | 0.0075 | 0.2130 | 0.1185 | 0.3989 | 0.1719 | — | — |
| 577 | 0.2493 | 0.0075 | 0.2135 | 0.1144 | 0.3989 | 0.1785 | — | — |
| 578 | 0.2647 | 0.0075 | 0.2103 | 0.1105 | 0.4451 | 0.1852 | — | — |
| 579 | 0.3096 | 0.0075 | 0.2050 | 0.1067 | 0.4278 | 0.1920 | — | — |
| 580 | 0.2734 | 0.0075 | 0.1790 | 0.1030 | 0.4305 | 0.1989 | — | — |
| 581 | 0.2779 | 0.0076 | 0.1831 | 0.0996 | 0.4366 | 0.2059 | — | — |
| 582 | 0.2750 | 0.0076 | 0.1914 | 0.0962 | 0.4472 | 0.2130 | — | — |
| 583 | 0.3395 | 0.0077 | 0.1894 | 0.0930 | 0.4469 | 0.2202 | — | — |
| 584 | 0.3361 | 0.0078 | 0.2510 | 0.0900 | 0.4467 | 0.2275 | — | — |
| 585 | 0.3387 | 0.0078 | 0.2553 | 0.0871 | 0.4860 | 0.2348 | — | — |
| 586 | 0.4142 | 0.0079 | 0.2548 | 0.0843 | 0.5149 | 0.2422 | — | — |
| 587 | 0.4677 | 0.0080 | 0.2984 | 0.0817 | 0.5526 | 0.2497 | — | — |
| 588 | 0.4807 | 0.0081 | 0.2851 | 0.0792 | 0.5572 | 0.2572 | — | — |
| 589 | 0.5063 | 0.0081 | 0.3538 | 0.0769 | 0.5977 | 0.2649 | — | — |
| 590 | 0.5665 | 0.0082 | 0.3504 | 0.0747 | 0.6219 | 0.2725 | — | — |
| 591 | 0.6026 | 0.0083 | 0.4005 | 0.0726 | 0.6026 | 0.2802 | — | — |
| 592 | 0.6019 | 0.0083 | 0.4004 | 0.0707 | 0.6273 | 0.2880 | — | — |
| 593 | 0.6019 | 0.0084 | 0.4009 | 0.0689 | 0.6551 | 0.2957 | — | — |
| 594 | 0.5981 | 0.0085 | 0.3971 | 0.0672 | 0.6649 | 0.3035 | — | — |
| 595 | 0.5488 | 0.0085 | 0.3958 | 0.0657 | 0.6186 | 0.3113 | — | — |
| 596 | 0.6855 | 0.0086 | 0.3956 | 0.0643 | 0.5886 | 0.3191 | — | — |
| 597 | 0.6946 | 0.0087 | 0.3967 | 0.0630 | 0.7036 | 0.3270 | — | — |
| 598 | 0.7435 | 0.0087 | 0.4599 | 0.0618 | 0.7004 | 0.3347 | — | — |
| 599 | 0.7194 | 0.0088 | 0.4592 | 0.0608 | 0.7137 | 0.3425 | — | — |
| 600 | 0.7188 | 0.0088 | 0.4481 | 0.0599 | 0.7157 | 0.3503 | — | — |
| 601 | 0.7100 | 0.0089 | 0.4444 | 0.0591 | 0.7117 | 0.3580 | — | — |
| 602 | 0.7352 | 0.0090 | 0.4367 | 0.0584 | 0.7162 | 0.3657 | — | — |
| 603 | 0.7005 | 0.0090 | 0.4361 | 0.0578 | 0.6640 | 0.3733 | — | — |
| 604 | 0.7689 | 0.0091 | 0.5091 | 0.0573 | 0.6677 | 0.3809 | — | — |
| 605 | 0.7568 | 0.0091 | 0.5089 | 0.0568 | 0.6676 | 0.3885 | — | — |
| 606 | 0.7435 | 0.0092 | 0.4707 | 0.0565 | 0.6792 | 0.3960 | — | — |
| 607 | 0.7338 | 0.0092 | 0.5365 | 0.0562 | 0.6795 | 0.4035 | — | — |
| 608 | 0.7789 | 0.0092 | 0.5061 | 0.0560 | 0.6925 | 0.4109 | — | — |
| 609 | 0.7759 | 0.0093 | 0.5455 | 0.0559 | 0.6793 | 0.4182 | — | — |
| 610 | 0.8224 | 0.0093 | 0.5454 | 0.0558 | 0.7084 | 0.4255 | — | — |
| 611 | 0.8196 | 0.0094 | 0.6165 | 0.0557 | 0.7377 | 0.4327 | — | — |
| 612 | 0.8496 | 0.0094 | 0.6078 | 0.0556 | 0.7377 | 0.4398 | — | — |
| 613 | 0.8741 | 0.0095 | 0.5346 | 0.0555 | 0.6961 | 0.4469 | — | — |
| 614 | 0.8351 | 0.0095 | 0.4511 | 0.0554 | 0.7156 | 0.4538 | — | — |
| 615 | 0.8470 | 0.0095 | 0.3966 | 0.0553 | 0.6956 | 0.4607 | — | — |
| 616 | 0.8470 | 0.0096 | 0.3966 | 0.0552 | 0.6410 | 0.4675 | — | — |
| 617 | 0.7811 | 0.0096 | 0.3296 | 0.0551 | 0.5729 | 0.4743 | — | — |
| 618 | 0.7803 | 0.0096 | 0.4801 | 0.0550 | 0.5900 | 0.4809 | — | — |
| 619 | 0.8006 | 0.0097 | 0.5240 | 0.0548 | 0.5920 | 0.4874 | — | — |
| 620 | 0.8984 | 0.0097 | 0.5200 | 0.0546 | 0.6082 | 0.4938 | — | — |
| 621 | 0.8984 | 0.0097 | 0.5953 | 0.0545 | 0.5880 | 0.5002 | — | — |
| 622 | 0.8528 | 0.0098 | 0.6276 | 0.0542 | 0.5785 | 0.5064 | — | — |
| 623 | 0.7880 | 0.0098 | 0.6776 | 0.0540 | 0.6183 | 0.5125 | — | — |
| 624 | 0.8090 | 0.0098 | 0.6466 | 0.0538 | 0.6131 | 0.5185 | — | — |
| 625 | 0.8103 | 0.0098 | 0.6553 | 0.0535 | 0.6128 | 0.5243 | — | — |
| 626 | 0.7467 | 0.0098 | 0.5781 | 0.0532 | 0.5790 | 0.5301 | — | — |
| 627 | 0.7936 | 0.0098 | 0.6260 | 0.0529 | 0.5289 | 0.5357 | — | — |
| 628 | 0.7554 | 0.0098 | 0.6172 | 0.0525 | 0.5263 | 0.5412 | — | — |
| 629 | 0.8035 | 0.0098 | 0.6146 | 0.0522 | 0.5222 | 0.5465 | — | — |
| 630 | 0.8207 | 0.0098 | 0.6904 | 0.0517 | 0.5180 | 0.5517 | — | — |
| 631 | 0.7746 | 0.0098 | 0.6903 | 0.0513 | 0.4724 | 0.5568 | — | — |
| 632 | 0.7629 | 0.0098 | 0.7167 | 0.0508 | 0.4631 | 0.5618 | — | — |
| 633 | 0.7099 | 0.0098 | 0.7545 | 0.0503 | 0.4445 | 0.5666 | — | — |
| 634 | 0.6906 | 0.0098 | 0.7507 | 0.0498 | 0.4051 | 0.5713 | — | — |
| 635 | 0.8288 | 0.0098 | 0.6829 | 0.0492 | 0.4261 | 0.5758 | — | — |
| 636 | 0.7862 | 0.0098 | 0.6174 | 0.0486 | 0.4710 | 0.5802 | — | — |
| 637 | 0.7259 | 0.0097 | 0.6174 | 0.0480 | 0.4654 | 0.5844 | — | — |
| 638 | 0.6346 | 0.0097 | 0.6383 | 0.0474 | 0.5150 | 0.5885 | — | — |
| 639 | 0.6344 | 0.0097 | 0.6373 | 0.0468 | 0.5100 | 0.5925 | — | — |
| 640 | 0.6343 | 0.0096 | 0.5534 | 0.0462 | 0.5084 | 0.5963 | — | — |
| 641 | 0.6195 | 0.0096 | 0.5536 | 0.0456 | 0.5333 | 0.6000 | — | — |
| 642 | 0.6035 | 0.0095 | 0.5569 | 0.0449 | 0.5303 | 0.6035 | — | — |
| 643 | 0.6066 | 0.0095 | 0.5621 | 0.0442 | 0.5472 | 0.6068 | — | — |
| 644 | 0.6945 | 0.0094 | 0.5563 | 0.0436 | 0.5964 | 0.6099 | — | — |
| 645 | 0.7638 | 0.0094 | 0.4872 | 0.0429 | 0.5751 | 0.6129 | — | — |
| 646 | 0.7581 | 0.0093 | 0.4884 | 0.0422 | 0.5826 | 0.6156 | — | — |
| 647 | 0.6792 | 0.0093 | 0.4880 | 0.0415 | 0.6260 | 0.6183 | — | — |
| 648 | 0.6444 | 0.0092 | 0.4550 | 0.0408 | 0.5726 | 0.6207 | — | — |
| 649 | 0.6406 | 0.0091 | 0.4551 | 0.0401 | 0.5676 | 0.6229 | — | — |
| 650 | 0.6204 | 0.0091 | 0.4951 | 0.0394 | 0.5682 | 0.6250 | — | — |
| 651 | 0.5672 | 0.0090 | 0.4343 | 0.0387 | 0.5294 | 0.6269 | — | — |
| 652 | 0.4684 | 0.0089 | 0.4489 | 0.0380 | 0.5034 | 0.6285 | — | — |
| 653 | 0.4962 | 0.0088 | 0.4394 | 0.0373 | 0.4919 | 0.6300 | — | — |
| 654 | 0.3329 | 0.0088 | 0.4525 | 0.0366 | 0.4711 | 0.6313 | — | — |
| 655 | 0.4306 | 0.0087 | 0.3999 | 0.0359 | 0.4924 | 0.6324 | — | — |
| 656 | 0.4315 | 0.0086 | 0.3984 | 0.0352 | 0.5065 | 0.6333 | — | — |
| 657 | 0.3702 | 0.0085 | 0.3984 | 0.0345 | 0.4873 | 0.6340 | — | — |
| 658 | 0.3685 | 0.0084 | 0.4021 | 0.0337 | 0.4872 | 0.6345 | — | — |
| 659 | 0.4195 | 0.0083 | 0.4545 | 0.0330 | 0.4854 | 0.6348 | — | — |
| 660 | 0.3298 | 0.0082 | 0.4546 | 0.0323 | 0.4782 | 0.6349 | — | — |
| 661 | 0.4011 | 0.0082 | 0.4468 | 0.0316 | 0.4412 | 0.6348 | — | — |
| 662 | 0.3719 | 0.0081 | 0.3808 | 0.0309 | 0.4530 | 0.6346 | — | — |
| 663 | 0.4474 | 0.0080 | 0.3812 | 0.0302 | 0.4532 | 0.6342 | — | — |
| 664 | 0.4377 | 0.0079 | 0.3567 | 0.0295 | 0.4499 | 0.6336 | — | — |
| 665 | 0.4763 | 0.0078 | 0.3583 | 0.0288 | 0.4310 | 0.6328 | — | — |
| 666 | 0.4505 | 0.0077 | 0.3590 | 0.0281 | 0.4269 | 0.6319 | — | — |
| 667 | 0.4855 | 0.0077 | 0.3731 | 0.0274 | 0.4482 | 0.6308 | — | — |
| 668 | 0.3476 | 0.0076 | 0.3776 | 0.0266 | 0.4495 | 0.6296 | — | — |
| 669 | 0.3766 | 0.0075 | 0.3591 | 0.0259 | 0.4501 | 0.6282 | — | — |
| 670 | 0.3333 | 0.0074 | 0.3968 | 0.0252 | 0.4501 | 0.6266 | — | — |
| 671 | 0.3344 | 0.0073 | 0.2985 | 0.0245 | 0.4875 | 0.6249 | — | — |
| 672 | 0.3682 | 0.0073 | 0.2558 | 0.0238 | 0.4884 | 0.6230 | — | — |
| 673 | 0.4108 | 0.0072 | 0.3304 | 0.0231 | 0.5016 | 0.6210 | — | — |
| 674 | 0.4581 | 0.0071 | 0.3286 | 0.0223 | 0.4734 | 0.6189 | — | — |
| 675 | 0.4648 | 0.0070 | 0.2625 | 0.0216 | 0.4439 | 0.6166 | — | — |
| 676 | 0.5537 | 0.0070 | 0.3259 | 0.0209 | 0.4629 | 0.6142 | — | — |
| 677 | 0.5263 | 0.0069 | 0.3237 | 0.0202 | 0.4444 | 0.6116 | — | — |
| 678 | 0.5701 | 0.0068 | 0.3251 | 0.0195 | 0.4118 | 0.6090 | — | — |
| 679 | 0.5701 | 0.0067 | 0.3806 | 0.0188 | 0.3817 | 0.6062 | — | — |
| 680 | 0.5700 | 0.0067 | 0.3804 | 0.0180 | 0.3982 | 0.6034 | — | — |
| 681 | 0.6609 | 0.0066 | 0.3791 | 0.0173 | 0.4530 | 0.6004 | — | — |
| 682 | 0.6880 | 0.0065 | 0.3008 | 0.0166 | 0.4362 | 0.5974 | — | — |
| 683 | 0.6852 | 0.0064 | 0.2570 | 0.0159 | 0.4362 | 0.5943 | — | — |
| 684 | 0.7522 | 0.0064 | 0.2696 | 0.0152 | 0.4224 | 0.5911 | — | — |
| 685 | 0.7466 | 0.0063 | 0.1947 | 0.0145 | 0.4250 | 0.5878 | — | — |
| 686 | 0.7953 | 0.0062 | 0.1949 | 0.0138 | 0.4443 | 0.5844 | — | — |
| 687 | 0.7793 | 0.0061 | 0.1308 | 0.0131 | 0.4056 | 0.5810 | — | — |
| 688 | 0.7486 | 0.0060 | 0.1319 | 0.0124 | 0.4120 | 0.5775 | — | — |
| 689 | 0.7405 | 0.0060 | 0.1383 | 0.0117 | 0.4123 | 0.5739 | — | — |
| 690 | 0.8819 | 0.0059 | 0.1660 | 0.0110 | 0.4347 | 0.5702 | — | — |
| 691 | 0.7748 | 0.0058 | 0.1447 | 0.0103 | 0.4352 | 0.5665 | — | — |
| 692 | 0.8093 | 0.0057 | 0.1427 | 0.0097 | 0.4379 | 0.5627 | — | — |
| 693 | 0.7487 | 0.0057 | 0.1463 | 0.0090 | 0.4426 | 0.5589 | — | — |
| 694 | 0.7134 | 0.0056 | 0.0753 | 0.0084 | 0.4426 | 0.5550 | — | — |
| 695 | 0.7125 | 0.0055 | 0.1324 | 0.0077 | 0.4529 | 0.5511 | — | — |
| 696 | 0.7375 | 0.0055 | 0.1310 | 0.0071 | 0.4666 | 0.5472 | — | — |
| 697 | 0.8385 | 0.0054 | 0.1578 | 0.0065 | 0.4674 | 0.5431 | — | — |
| 698 | 0.8845 | 0.0054 | 0.1582 | 0.0059 | 0.4858 | 0.5391 | — | — |
| 699 | 0.7800 | 0.0053 | 0.2281 | 0.0054 | 0.4309 | 0.5350 | — | — |
| 700 | 0.8226 | 0.0053 | 0.2938 | 0.0049 | 0.4035 | 0.5309 | — | — |
| 701 | 0.8224 | 0.0052 | 0.2898 | 0.0044 | 0.3895 | 0.5268 | — | — |
| 702 | 0.8648 | 0.0052 | 0.2766 | 0.0039 | 0.3681 | 0.5227 | — | — |
| 703 | 0.8646 | 0.0052 | 0.2760 | 0.0034 | 0.3731 | 0.5185 | — | — |
| 704 | 0.8837 | 0.0052 | 0.2779 | 0.0030 | 0.3622 | 0.5143 | — | — |
| 705 | 0.8381 | 0.0052 | 0.3377 | 0.0025 | 0.3374 | 0.5101 | — | — |
| 706 | 0.8389 | 0.0052 | 0.3367 | 0.0021 | 0.3026 | 0.5059 | — | — |
| 707 | 0.8389 | 0.0053 | 0.3481 | 0.0018 | 0.2857 | 0.5017 | — | — |
| 708 | 0.8423 | 0.0053 | 0.3759 | 0.0015 | 0.2551 | 0.4975 | — | — |
| 709 | 0.8423 | 0.0054 | 0.3630 | 0.0012 | 0.2765 | 0.4933 | — | — |
| 710 | 0.8527 | 0.0055 | 0.3626 | 0.0009 | 0.2765 | 0.4892 | — | — |
| 711 | 0.8521 | 0.0057 | 0.3629 | 0.0007 | 0.2711 | 0.4850 | — | — |
| 712 | 0.8907 | 0.0059 | 0.3895 | 0.0005 | 0.3246 | 0.4808 | — | — |
| 713 | 0.8888 | 0.0061 | 0.3884 | 0.0004 | 0.3012 | 0.4766 | — | — |
| 714 | 0.9157 | 0.0063 | 0.3499 | 0.0002 | 0.3016 | 0.4725 | — | — |
| 715 | 0.9558 | 0.0066 | 0.3667 | 0.0002 | 0.3347 | 0.4684 | — | — |
| 716 | 0.9563 | 0.0069 | 0.3667 | 0.0001 | 0.3277 | 0.4643 | — | — |
| 717 | 0.9222 | 0.0073 | 0.3073 | 0.0000 | 0.2571 | 0.4602 | — | — |
| 718 | 0.9951 | 0.0078 | 0.2981 | 0.0000 | — | — | — | — |
| 719 | 0.8319 | 0.0083 | 0.2920 | 0.0000 | — | — | — | — |
| 720 | 0.8647 | 0.0089 | 0.2931 | 0.0000 | — | — | — | — |
| 721 | 0.8944 | 0.0095 | 0.2931 | 0.0001 | — | — | — | — |
| 722 | 0.8946 | 0.0103 | 0.2933 | 0.0001 | — | — | — | — |
| 723 | 0.8951 | 0.0110 | 0.3202 | 0.0002 | — | — | — | — |
| 724 | 0.8951 | 0.0119 | 0.3202 | 0.0003 | — | — | — | — |
| 725 | 0.8518 | 0.0128 | 0.3343 | 0.0004 | — | — | — | — |
| 726 | 0.8509 | 0.0139 | 0.4162 | 0.0005 | — | — | — | — |
| 727 | 0.7994 | 0.0150 | 0.5014 | 0.0007 | — | — | — | — |
| 728 | 0.8092 | 0.0162 | 0.5410 | 0.0009 | — | — | — | — |
| 729 | 0.7788 | 0.0174 | 0.5406 | 0.0010 | — | — | — | — |
| 730 | 0.7811 | 0.0188 | 0.5398 | 0.0012 | — | — | — | — |
| 731 | 0.7358 | 0.0203 | 0.5717 | 0.0014 | — | — | — | — |
| 732 | 0.7960 | 0.0219 | 0.4947 | 0.0017 | — | — | — | — |
| 733 | 0.7974 | 0.0236 | 0.5142 | 0.0019 | — | — | — | — |
| 734 | 0.8542 | 0.0254 | 0.4448 | 0.0022 | — | — | — | — |
| 735 | 0.8544 | 0.0272 | 0.4769 | 0.0025 | — | — | — | — |
| 736 | 0.8203 | 0.0292 | 0.5209 | 0.0028 | — | — | — | — |
| 737 | 0.8711 | 0.0312 | 0.3855 | 0.0031 | — | — | — | — |
| 738 | 0.8238 | 0.0334 | 0.3797 | 0.0034 | — | — | — | — |
| 739 | 0.8208 | 0.0356 | 0.3759 | 0.0037 | — | — | — | — |
| 740 | 0.7334 | 0.0379 | 0.4131 | 0.0041 | — | — | — | — |
| 741 | 0.7336 | 0.0403 | 0.4956 | 0.0045 | — | — | — | — |
| 742 | 0.7290 | 0.0427 | 0.4961 | 0.0049 | — | — | — | — |
| 743 | 0.7290 | 0.0452 | 0.4961 | 0.0053 | — | — | — | — |
| 744 | 0.7291 | 0.0478 | 0.4963 | 0.0057 | — | — | — | — |
| 745 | 0.7143 | 0.0504 | 0.4978 | 0.0062 | — | — | — | — |
| 746 | 0.6887 | 0.0531 | 0.4974 | 0.0067 | — | — | — | — |
| 747 | 0.6886 | 0.0558 | 0.4974 | 0.0072 | — | — | — | — |
| 748 | 0.5959 | 0.0586 | 0.4911 | 0.0077 | — | — | — | — |
| 749 | 0.6014 | 0.0615 | 0.4912 | 0.0082 | — | — | — | — |
| 750 | 0.6016 | 0.0644 | 0.4929 | 0.0088 | — | — | — | — |
| 751 | 0.6040 | 0.0673 | 0.4942 | 0.0094 | — | — | — | — |
| 752 | 0.5847 | 0.0703 | 0.5736 | 0.0100 | — | — | — | — |
| 753 | 0.5925 | 0.0733 | 0.5718 | 0.0107 | — | — | — | — |
| 754 | 0.5147 | 0.0764 | 0.5522 | 0.0113 | — | — | — | — |
| 755 | 0.5766 | 0.0795 | 0.5524 | 0.0121 | — | — | — | — |
| 756 | 0.5803 | 0.0827 | 0.5545 | 0.0128 | — | — | — | — |
| 757 | 0.5957 | 0.0859 | 0.5545 | 0.0136 | — | — | — | — |
| 758 | 0.6382 | 0.0891 | 0.4781 | 0.0144 | — | — | — | — |
| 759 | 0.6391 | 0.0924 | — | — | — | — | — | — |
| 760 | 0.5395 | 0.0957 | — | — | — | — | — | — |

**Supplementary Table 8. Important adaptive codon sites with experimental validation in RdRp genes.**

| Genes and Sites | | | | Title | Authors | Journal | Publication_year |
| --- | --- | --- | --- | --- | --- | --- | --- |
| *PB2* | *PB1* | *PA* | *NP* |  |  |  |  |
| — | — | 100, 120, 321, 330, 639 | — | A Eurasian avian-like H1N1 swine influenza reassortant virus became pathogenic and highly transmissible due to mutations in its PA gene | Meng F, Yang H, Qu Z et al. | Proc Natl Acad Sci U S A | 2022 |
| — | 577 | — | — | A PB1-K577E Mutation in H9N2 Influenza Virus Increases Polymerase Activity and Pathogenicity in Mice | Kamiki H, Matsugo H, Kobayashi T et al. | Viruses | 2018 |
| — | 66 | — | — | A single mutation in the PB1-F2 of H5N1 (HK/97) and 1918 influenza A viruses contributes to increased virulence | Conenello GM, Zamarin D, Perrone LA et al. | PLoS Pathog | 2007 |
| 590,591 | — | — | — | Adaptive strategies of the influenza virus polymerase for replication in humans | Mehle A, Doudna JA | Proc Natl Acad Sci U S A | 2009 |
| — | — | — | 286, 437 | Amino Acid Mutations A286V and T437M in the Nucleoprotein Attenuate H7N9 Viruses in Mice | Ma S, Zhang B, Shi J et al. | J Virol | 2020 |
| 55, 590, 591, 627, 701, 702 | 66, 375 | 552 | — | Analysis by single-gene reassortment demonstrates that the 1918 influenza virus is functionally compatible with a low-pathogenicity avian influenza virus in mice | Qi L, Davis AS, Jagger BW et al. | J Virol | 2012 |
| — | 621 | — | 351 | Ancestral sequence reconstruction pinpoints adaptations that enable avian influenza virus transmission in pigs | Su W, Harfoot R, Su Y et al. | Nat Microbiol | 2021 |
| — | 375 | — | — | Characterization of the 1918 influenza virus polymerase genes | Taubenberger JK, Reid AH, Lourens RM et al. | Nature | 2005 |
| 89, 526, 588 | — | — | — | Dynamic Variation and Reversion in the Signature Amino Acids of H7N9 Virus During Human Infection | Zou X, Guo Q, Zhang W et al. | J Infect Dis | 2018 |
| 627,701 | — | — | — | Dynamic PB2-E627K substitution of influenza H7N9 virus indicates the in vivo genetic tuning and rapid host adaptation | Liu WJ, Li J, Zou R et al. | Proc Natl Acad Sci U S A | 2020 |
| 73 | — | 55, 617, 618, 683 | — | Evolution of Swine Influenza Virus H3N2 in Vaccinated and Nonvaccinated Pigs after Previous Natural H1N1 Infection | Lopez-Valinas A, Baioni L, Cordoba L et al. | Viruses | 2022 |
| 535 | — | — | — | Highly pathogenic avian influenza A(H5N1) virus infection in foxes with PB2-M535I identified as a novel mammalian adaptation, Northern Ireland, July 2023 | Lagan P, Mckenna R, Baleed S et al. | Euro Surveill | 2023 |
| — | — | — | 91, 98, 227, 229, 470 | Histone Deacetylase 1 Plays an Acetylation-Independent Role in Influenza A Virus Replication | Chen L, Wang C, Luo J et al. | Front Immunol | 2017 |
| 271, 661, 683, 684 | — | — | — | Identification of Influenza A Virus PB2 Residues Involved in Enhanced Polymerase Activity and Virus Growth in Mammalian Cells at Low Temperatures | Hayashi T, Wills S, Bussey KA et al. | J Virol | 2015 |
| 156, 271, 292, 627 | — | — | — | Identification of Key Amino Acids in the PB2 and M1 Proteins of H7N9 Influenza Virus That Affect Its Transmission in Guinea Pigs | Kong H, Ma S, Wang J et al. | J Virol | 2019 |
| 192, 627, 701, 702 | 105 | — | — | Identification of mammalian-adapting mutations in the polymerase complex of an avian H5N1 influenza virus | Taft AS, Ozawa M, Fitch A et al. | Nat Commun | 2015 |
| — | — | 38 | — | Identification of the I38T PA Substitution as a Resistance Marker for Next-Generation Influenza Virus Endonuclease Inhibitors | Jones JC, Kumar G, Barman S et al. | mBio | 2018 |
| — | 99 | — | — | Identification, characterization, and natural selection of mutations driving airborne transmission of A/H5N1 virus | Linster M, van Boheemen S, de Graaf M et al. | Cell | 2014 |
| — | — | — | 31, 90 | Influenza A virus nucleoprotein is acetylated by histone acetyltransferases PCAF and GCN5 | Hatakeyama D, Shoji M, Yamayoshi S et al. | J Biol Chem | 2018 |
| 627 | — | — | — | Influenza virus adaptation PB2-627K modulates nucleocapsid inhibition by the pathogen sensor RIG-I | Weber M, Sediri H, Felgenhauer U et al. | Cell Host Microbe | 2015 |
| — | — | — | 346 | Interactions between Influenza A Virus Nucleoprotein and Gene Segment Untranslated Regions Facilitate Selective Modulation of Viral Gene Expression | Diefenbacher M, Tan T, Bauer D et al. | J Virol | 2022 |
| — | — | — | 357 | Mammalian-adaptive mutation NP-Q357K in Eurasian H1N1 Swine Influenza viruses determines the virulence phenotype in mice | Zhu W, Feng Z, Chen Y et al. | Emerg Microbes Infect | 2019 |
| — | — | — | 165, 416 | Monomeric nucleoprotein of influenza A virus | Chenavas S, Estrozi LF, Slama-Schwok A et al. | PLoS Pathog | 2013 |
| — | — | 97 | — | Multiple amino acid substitutions involved in the adaption of three avian-origin H7N9 influenza viruses in mice | Qin J, Peng O, Shen X et al. | Virol J | 2019 |
| 286, 534, 591 | — | — | — | Multiple Natural Substitutions in Avian Influenza A Virus PB2 Facilitate Efficient Replication in Human Cells | Manz B, de Graaf M, Mogling R et al. | J Virol | 2016 |
| — | 216 | — | — | Naturally occurring mutations in PB1 affect influenza A virus replication fidelity, virulence, and adaptability | Lin RW, Chen GW, Sung HH et al. | J Biomed Sci | 2019 |
| — | — | 18 | — | PA-E18G substitution in influenza A virus confers resistance to ZX-7101, a cap-dependent endonuclease inhibitor | Luo D, Ye Q, Li RT et al. | Virol Sin | 2023 |
| — | 222, 524 | — | — | PB1 S524G mutation of wild bird-origin H3N8 influenza A virus enhances virulence and fitness for transmission in mammals | Zhang X, Li Y, Jin S et al. | Emerg Microbes Infect | 2021 |
| 271 | — | — | — | PB2 residue 271 plays a key role in enhanced polymerase activity of influenza A viruses in mammalian host cells | Bussey KA, Bousse TL, Desmet EA et al. | J Virol | 2010 |
| 591 | — | — | — | PB2-Q591K Mutation Determines the Pathogenicity of Avian H9N2 Influenza Viruses for Mammalian Species | Wang C, Lee HH, Yang ZF et al. | PLoS One | 2016 |
| — | — | — | 407,413 | Phosphorylation controls the nuclear-cytoplasmic shuttling of influenza A virus nucleoprotein | Zheng W, Li J, Wang S et al. | J Virol | 2015 |
| — | — | — | 482 | PLK3 facilitates replication of swine influenza virus by phosphorylating viral NP protein | Ren C, Chen T, Zhang S et al. | Emerg Microbes Infect | 2023 |
| — | — | 552 | — | Reassortment and mutation of the avian influenza virus polymerase PA subunit overcome species barriers | Mehle A, Dugan VG, Taubenberger JK et al. | J Virol | 2012 |
| — | — | — | 165, 402, 403, 457 | Regulation of influenza A virus nucleoprotein oligomerization by phosphorylation | Turrell L, Hutchinson EC, Vreede FT et al. | J Virol | 2015 |
| — | — | — | 305, 313, 357 | Role of host-specific amino acids in the pathogenicity of avian H5N1 influenza viruses in mice | Kim JH, Hatta M, Watanabe S et al. | J Gen Virol | 2010 |
| — | — | — | 77, 113, 229 | Role of influenza A virus NP acetylation on viral growth and replication | Giese S, Ciminski K, Bolte H et al. | Nat Commun | 2017 |
| 701 | — | — | — | Substitution of D701N in the PB2 protein could enhance the viral replication and pathogenicity of Eurasian avian-like H1N1 swine influenza viruses | Liu S, Zhu W, Feng Z et al. | Emerg Microbes Infect | 2018 |
| — | — | 224, 383 | — | Synergistic Effect of S224P and N383D Substitutions in the PA of H5N1 Avian Influenza Virus Contributes to Mammalian Adaptation | Song J, Xu J, Shi J et al. | Sci Rep | 2015 |
| — | — | — | 184 | The mechanism by which influenza A virus nucleoprotein forms oligomers and binds RNA | Ye Q, Krug RM, Tao YJ | Nature | 2006 |
| — | 82 | — | — | Tyr82 Amino Acid Mutation in PB1 Polymerase Induces an Influenza Virus Mutator Phenotype | Naito T, Shirai K, Mori K et al. | J Virol | 2019 |
| — | — | — | 184 | Ubiquitination and deubiquitination of NP protein regulates influenza A virus RNA replication | Liao TL, Wu CY, Su WC et al. | EMBO J | 2010 |
| — | 612 | — | — | Viral RNA-binding ability conferred by SUMOylation at PB1 K612 of influenza A virus is essential for viral pathogenesis and transmission | Li J, Liang L, Jiang L et al. | PLoS Pathog | 2021 |

Note: The Table presents important codon locations of the RdRp genes that have been mentioned in the relevant literature. The numbers in the Table represent the positional information of the important codon, while "—" denotes that there is no mention of the significant codon for this segment in the literature.

**Supplementary Table 9. The predicted results of simulated reassortment adaptation for H5N1, H7N9, and H9N2.**

| Simulated type | Subtype | | |
| --- | --- | --- | --- |
|  | H5N1 | H7N9 | H9N2 |
| Total | 6199 | 2367 | 1622 |
| ###1 | 5126 | 2296 | 1489 |
| ##1# | 4318 | 1821 | 834 |
| #1## | 5357 | 53 | 477 |
| 1### | 21 | 6 | 69 |
| ##11 | 4 | 3 | 12 |
| #1#1 | 414 | 2 | 59 |
| #11# | 322 | 2 | 53 |
| 1##1 | 3 | 3 | 0 |
| 1#1# | 1 | 3 | 1 |
| 11## | 2 | 2 | 7 |
| #111 | 1 | 2 | 1 |
| 1#11 | 1 | 2 | 0 |
| 11#1 | 1 | 2 | 0 |
| 111# | 1 | 2 | 1 |

Note: "#" represents the segment sequence from H3N2,

and "1" represents the replaced sequence from

the corresponding avian IAVs.

**Supplementary Table 10. The names of IAV strains in the phylogenetic tree.**

| Name in the phylogenetic tree | Original name |
| --- | --- |
| 001_USA_American_Crow | A/American Crow/North Dakota/22-010665-001/2022 |
| 002_USA_Backyard_bird | A/Backyard bird/Alaska/22-013107-001/2022 |
| 003_USA_Backyard_bird | A/Backyard bird/Michigan/22-008890-004/2022 |
| 004_Uruguay_Backyard_chicken | A/Backyard chicken/Uruguay/124-M1/2023 |
| 005_USA_Bald_Eagle | A/Bald Eagle/North Carolina/22-007917-003/2022 |
| 006_Canada_Bald_Eagle | A/Bald Eagle/ON/FAV-0178-02/2022 |
| 007_USA_Bald_Eagle | A/bald eagle/Washington/24-007953-001/2024 |
| 008_Canada_Bald_Eagle | A/bald_eagle/BC/AIVPHL-15/2022 |
| 009_USA_black_duck | A/black duck/Tennessee/17OS0306/2017 |
| 010_USA_black_vulture | A/black vulture/Louisiana/W23-166/2023 |
| 011_USA_blackbird | A/blackbird/Texas/24-008354-001/2024 |
| 012_USA_blackbird | A/blackbird/Texas/24-008357-001/2024 |
| 013_USA_bovine | A/Bovine/texas/24-029328-01/2024 |
| 014_USA_Canada_Goose | A/Canada goose/Delaware Bay/601/2016 |
| 015_USA_Canada_Goose | A/canada goose/Indiana/24-008719-001/2024 |
| 016_USA_Canada_Goose | A/Canada Goose/Michigan/22-008506-008/2022 |
| 017_USA_Canada_Goose | A/canada goose/Missouri/24-006385-003/2024 |
| 018_Canada_Canada_Goose | A/Canada Goose/ON/FAV-0187-01/2022 |
| 019_Bangladesh_chicken | A/chicken/Bangladesh/10C463/2022 |
| 020_Bangladesh_chicken | A/chicken/Bangladesh/22482/2014 |
| 021_Bangladesh_chicken | A/chicken/Bangladesh/30065/2016 |
| 022_Burkina_Faso_chicken | A/chicken/Burkina_Faso/21VIR11911-5/2021 |
| 023_Cameroon_chicken | A/chicken/Cameroon/16VIR3791-22/2016 |
| 024_Egypt_chicken | A/chicken/Egypt/S3806D/2011 |
| 025_China_chicken | A/chicken/Hubei/wl/1997 |
| 026_Egypt_chicken | A/chicken/Ismailia/144CA/2014 |
| 027_China_chicken | A/chicken/Jilin/9/2004 |
| 028_China_chicken | A/chicken/Jilin/hd/2002 |
| 029_USA_chicken | A/chicken/Kansas/24-010482-001/2024 |
| 030_USA_chicken | A/chicken/Michigan/22-011521-004-original/2022 |
| 031_USA_chicken | A/chicken/Michigan/24-010308-012/2024 |
| 032_USA_chicken | A/chicken/Montana/22-013942-002-original/2022 |
| 033_USA_chicken | A/chicken/New Jersey/15-002659-2/2015 |
| 034_USA_chicken | A/chicken/New York/22-010321-002-original/2022 |
| 035_USA_chicken | A/chicken/Ohio/24-005334-001/2024 |
| 036_USA_chicken | A/chicken/Oklahoma/22-013220-002-original/2022 |
| 037_USA_chicken | A/chicken/Pennsylvania/22-011920-001/2022 |
| 038_Indonesia_chicken | A/chicken/South Kalimantan/UT6029/2006 |
| 039_USA_chicken | A/chicken/Texas/24-007264-001/2024 |
| 040_USA_chicken | A/chicken/Texas/24-009654-004/2024 |
| 041_Viet_Nam_chicken | A/chicken/Viet Nam/HU12-1328/2019 |
| 042_Viet_Nam_chicken | A/chicken/Vietnam/NCVD-008/2008 |
| 043_Viet_Nam_chicken | A/chicken/Vietnam/NCVD-KA448/2013 |
| 044_Korea_commom_teal | A/common teal/Korea/WA537/2022 |
| 045_Belgium_crested_eagle | A/crested eagle/Belgium/01/2004 |
| 046_USA_Crow | A/crow/Illinois/24-004479-001/2024 |
| 047_Kazakhstan_Cygnus_cygnus | A/Cygnus cygnus/Karakol lake/01/2024 |
| 048_USA_Dairy_cattle | A/Dairy cattle/Kansas/5/2024 |
| 049_USA_duck | A/domestic duck/Michigan/22-008890-001-original/2022 |
| 050_Bangladesh_duck | A/duck/Bangladesh/22811/2014 |
| 051_Bangladesh_duck | A/duck/Bangladesh/35826/2018 |
| 052_China_duck | A/duck/China/1033/2017 |
| 053_Egypt_duck | A/duck/Egypt/Q2645C/2010 |
| 054_France_duck | A/duck/France/05066b/2005 |
| 055_China_duck | A/duck/Guangxi/xa/2001 |
| 056_China_duck | A/duck/Hunan/3340/2006 |
| 057_USA_duck | A/Duck/Indiana/22-010624-001/2022 |
| 058_USA_duck | A/duck/Minnesota/1525/1981 |
| 059_China_duck | A/duck/Shandong/009/2008 |
| 060_Viet_Nam_duck | A/duck/Vietnam/205/2005 |
| 061_Viet_Nam_duck | A/duck/Vietnam/HU5-1575/2016 |
| 062_Viet_Nam_duck | A/duck/Vietnam/NCVD-15A60/2015 |
| 063_Viet_Nam_duck | A/duck/Vietnam/NCVD-KA310/2012 |
| 064_USA_feline | A/feline/Texas/24-029329-01/2024 |
| 065_USA_feline | A/feline/Texas/24-029329-02/2024 |
| 066_China_goose | A/goose/Guangdong/3/1997 |
| 067_Viet_Nam_goose | A/goose/Vietnam/3/05 |
| 068_USA_hooded_merganser | A/hooded merganser/South Carolina/W24-028/2024 |
| 069_Chile_Larosterna_inca | A/Inca tern/Antofagasta/238083/2023 |
| 070_Netherlands_mallard | A/mallard duck/Netherlands/41/2015 |
| 071_China_mallard | A/Mallard/Huadong/Y/2003 |
| 072_USA_mallard | A/mallard/Illinois/12OS5080/2012 |
| 073_USA_mallard | A/mallard/Minnesota/AI11-4429/2011 |
| 074_USA_mallard | A/mallard/Ohio/12OS2218/2012 |
| 075_Canada_mallard | A/mallard/Ontario/15741/2005 |
| 076_Canada_mallard | A/mallard/Ontario/26078/2007 |
| 077_USA_mallard | A/mallard/Wisconsin/10OS2672/2010 |
| 078_USA_mallard | A/mallard/Wisconsin/2576/2009 |
| 079_USA_mallard | A/mallard/Wisconsin/568/1982 |
| 080_Viet_Nam_muscovy_duck | A/muscovy duck/Vietnam/LBM113/2012 |
| 081_Kazakhstan_mute_swan | A/mute swan/Mangystau/1-S24R-2/2024 |
| 082_USA_mute_swan | A/mute swan/MI/451072-2/2006 |
| 083_Chile_Pelican | A/Pelican/Antofagasta/228244-2/2022 |
| 084_USA_Pigeon | A/pigeon/Texas/24-008765-001/2024 |
| 085_China_plateau_pika | A/plateau pika/Qinghai/04/2007 |
| 086_USA_ruddy_turnstone | A/ruddy turnstone/DE/509531/2007 |
| 087_Iceland_ruddy_turnstone | A/ruddy turnstone/Iceland/2899/2013 |
| 088_USA_Shorebird | A/shorebird/Delaware/472/2007 |
| 089_USA_Skunk | A/skunk/New Mexico/24-006483-001/2024 |
| 090_China_swine | A/swine/Shandong/2/03 |
| 091_USA_Homo_sapiens | A/Texas/37/2024 |
| 092_China_Tree_sparrow | A/Tree sparrow/Henan/4/2004 |
| 093_Egypt_turkey | A/turkey/Egypt/S6405A/2012 |
| 094_Italy_turkey | A/turkey/Italy/21VIR11803-1/2021 |
| 095_USA_turkey | A/turkey/Michigan/22-008890-007-original/2022 |
| 096_USA_turkey | A/turkey/Minnesota/23-030402-003-original/2023 |
| 097_USA_turkey | A/Turkey/South Dakota/22-006792-001/2022 |
| 098_China_wild_duck | A/wild duck/Jilin/ZF/2011 |
| 099_USA_wild_duck | A/wild duck/Ohio/623/2004 |
| 100_USA_wood_duck | A/wood duck/North Carolina/W24-026/2024 |

**Supplementary Table 11. Simulation repacking prediction results of the simulated generated sequence at the 627EK site.**

| ID | true label | prediction | probability |
| --- | --- | --- | --- |
| A/mallard/Maryland/11OS3603/2011_EtoK_AAG | 1 | 0 | 0.99998 |
| A/New York/WC-LVD-14-052/2014 | 1 | 1 | 0.99865 |
| A/New York/A-WC-LVD-16-013/2016_EtoK_AAG | 1 | 1 | 0.99942 |
| A/Thailand/SN10452/2010_KtoE_GAA | 0 | 1 | 0.99980 |
| A/Siena/14/1995_KtoE_GAG | 0 | 1 | 0.97501 |
| A/Berlin/86/2022_KtoE_GAA | 0 | 1 | 0.99987 |
| A/Texas/13088/2022_KtoE_GAA | 0 | 1 | 0.99985 |
| A/Texas/7775/2018_EtoK_AAG | 1 | 1 | 0.99966 |
| A/Berlin/223/2022 | 1 | 1 | 0.99985 |
| A/Maryland/62_10D/2013_KtoE_GAG | 0 | 1 | 0.99974 |
| A/England/221381025/2022 | 1 | 1 | 0.99986 |
| A/chicken/Jiangxi/19912/2013_EtoK_AAG | 1 | 0 | 0.99951 |
| A/Thailand/SN10560/2011_EtoK_AAA | 1 | 1 | 0.99957 |
| A/chicken/Jiangxi/9530/2014_EtoK_AAA | 1 | 0 | 0.97385 |
| A/Texas/90/2017_KtoE_GAA | 0 | 1 | 0.99983 |
| A/American_green-winged_teal/Maryland/06MD700/2006_EtoK_AAG | 1 | 0 | 0.99987 |
| A/Nicaragua/7302_09/2013_EtoK_AAA | 1 | 1 | 0.99971 |
| A/Boston/634/2009_EtoK_AAA | 1 | 1 | 0.99963 |
| A/New York/WC-LVD-12-043/2012_EtoK_AAG | 1 | 1 | 0.99979 |
| A/Berlin/223/2022_KtoE_GAA | 0 | 1 | 0.99984 |
| A/Chicago/YGA_04165/2013_KtoE_GAG | 0 | 1 | 0.99966 |
| A/duck/Pennsylvania/22-014337-007-original/2022_EtoK_AAA | 1 | 0 | 0.99936 |
| A/England/222000262/2022_EtoK_AAA | 1 | 1 | 0.99929 |
| A/Dakar/52/2021_EtoK_AAA | 1 | 1 | 0.99966 |
| A/New York/RVTNL7557587529757/2022 | 1 | 1 | 0.99984 |
| A/Michigan/UOM10045253733/2022_KtoE_GAA | 0 | 1 | 0.99988 |
| A/Maine/05/2015_KtoE_GAA | 0 | 1 | 0.99984 |
| A/Nicaragua/7965_13/2016_KtoE_GAA | 0 | 1 | 0.99981 |
| A/Human/New York City/PV60760/2022_KtoE_GAA | 0 | 1 | 0.99985 |
| A/mallard duck/Netherlands/12/2012_EtoK_AAG | 1 | 0 | 0.99797 |
| A/Dakar/52/2021_EtoK_AAG | 1 | 1 | 0.99967 |
| A/Netherlands/11656/2022 | 1 | 1 | 0.99982 |
| A/duck/Pennsylvania/22-014337-007-original/2022_EtoK_AAG | 1 | 0 | 0.99917 |
| A/Michigan/85/2018_KtoE_GAA | 0 | 1 | 0.99987 |
| A/New York/A-WC-LVD-16-013/2016 | 1 | 1 | 0.99922 |
| A/New York/781/1993_KtoE_GAA | 0 | 1 | 0.99990 |
| A/Alaska/18/2018_KtoE_GAG | 0 | 1 | 0.99988 |
| A/bufflehead/Alaska/594/2014_EtoK_AAG | 1 | 0 | 0.99991 |
| A/Singapore/DMS41/2009_EtoK_AAA | 1 | 1 | 0.99938 |
| A/Boston/606/2009 | 1 | 1 | 0.99962 |
| A/Iowa/23/2014_EtoK_AAA | 1 | 1 | 0.99931 |
| A/Washington/74/2018_KtoE_GAG | 0 | 1 | 0.99987 |
| A/domestic_duck/Czech_Republic/1398/2023_EtoK_AAG | 1 | 0 | 0.99994 |
| A/Singapore/GP730/2011_EtoK_AAG | 1 | 1 | 0.99974 |
| A/Wisconsin/629-D02448/2009_EtoK_AAA | 1 | 1 | 0.99955 |
| A/England/225160986/2022_KtoE_GAA | 0 | 1 | 0.99985 |
| A/HaNoi/HN1004/2003_KtoE_GAG | 0 | 1 | 0.99983 |
| A/Moscow/127-88V/2021 | 1 | 1 | 0.99987 |
| A/South Korea/8829/2019_KtoE_GAA | 0 | 1 | 0.99979 |
| A/Netherlands/11476/2022 | 1 | 1 | 0.99893 |
| A/Alabama/06/2022 | 1 | 1 | 0.99986 |
| A/chicken/Jiangxi/10875/2014_EtoK_AAA | 1 | 0 | 0.99578 |
| A/Sao Paulo/IAL/C8561/2021_KtoE_GAG | 0 | 1 | 0.99985 |
| A/Wisconsin/629-D02448/2009_EtoK_AAG | 1 | 1 | 0.99957 |
| A/Kentucky/UR06-0128/2007_KtoE_GAA | 0 | 1 | 0.99967 |
| A/Michigan/124/2019_EtoK_AAA | 1 | 1 | 0.99924 |
| A/Michigan/UOM10048602233/2022_KtoE_GAA | 0 | 1 | 0.99988 |
| A/domestic_duck/Czech_Republic/1398/2023 | 0 | 0 | 0.99995 |
| A/Galicia/22009514/2022_KtoE_GAG | 0 | 1 | 0.99980 |
| A/Iowa/14/2014_KtoE_GAG | 0 | 1 | 0.99977 |
| A/Dakar/52/2021 | 1 | 1 | 0.99961 |
| A/turkey/South Dakota/22-010135-002-original/2022_EtoK_AAG | 1 | 0 | 0.99998 |
| A/pheasant/Hong_Kong/NT458/2006 | 0 | 0 | 0.99992 |
| A/mallard/Maryland/11OS3701/2011_EtoK_AAG | 1 | 0 | 0.99998 |
| A/England/221320672/2022_KtoE_GAA | 0 | 1 | 0.99988 |
| A/mallard/Ohio/11OS2091/2011_EtoK_AAA | 1 | 0 | 0.99994 |
| A/Sydney/25/2021_KtoE_GAG | 0 | 1 | 0.99988 |
| A/Michigan/UOM10045473306/2022_KtoE_GAG | 0 | 1 | 0.99987 |
| A/mallard/Maryland/11OS3603/2011 | 0 | 0 | 0.99998 |
| A/Tumbes/FPT02312/2022(H3N2)_KtoE_GAG | 0 | 1 | 0.99985 |
| A/Washington/55/2015_KtoE_GAA | 0 | 1 | 0.99984 |
| A/environment/Bangladesh/15114/2012_EtoK_AAG | 1 | 0 | 0.99992 |
| A/Houston/2H/2009_EtoK_AAA | 1 | 1 | 0.99965 |
| A/Singapore/DMS41/2009 | 1 | 1 | 0.99918 |
| A/Wisconsin/629-S0229/2009_EtoK_AAA | 1 | 1 | 0.99965 |
| A/Michigan/UOM10048462611/2022 | 1 | 1 | 0.99987 |
| A/Ethiopia/2611/2022 | 1 | 1 | 0.99956 |
| A/California/36/2016_KtoE_GAA | 0 | 1 | 0.99984 |
| A/Wisconsin/629-D02448/2009 | 1 | 1 | 0.99949 |
| A/Kentucky/UR06-0128/2007 | 1 | 1 | 0.99977 |
| A/Delaware/24/2022_KtoE_GAG | 0 | 1 | 0.99986 |
| A/Singapore/DMS41/2009_EtoK_AAG | 1 | 1 | 0.99942 |
| A/Alaska/18/2018 | 1 | 1 | 0.99988 |
| A/New_York/52/2017_KtoE_GAG | 0 | 1 | 0.99987 |
| A/turkey/Minnesota/22-010652-001-original/2022_EtoK_AAA | 1 | 0 | 0.99997 |
| A/England/225180046/2022_KtoE_GAG | 0 | 1 | 0.99987 |
| A/mallard duck/Netherlands/43/2011 | 0 | 0 | 0.99997 |
| A/Human/New York City/PV42586/2021_KtoE_GAG | 0 | 1 | 0.99986 |
| A/mallard duck/Netherlands/43/2011_EtoK_AAA | 1 | 0 | 0.99995 |
| A/Rhode_Island/19/2018 | 1 | 1 | 0.99901 |
| A/Michigan/UOM10042700269/2021 | 1 | 1 | 0.99986 |
| A/Connecticut/31/2018 | 1 | 1 | 0.99987 |
| A/Siena/14/1995_KtoE_GAA | 0 | 1 | 0.99680 |
| A/Thailand/CU-B97/2009_KtoE_GAG | 0 | 1 | 0.99978 |
| A/guineafowl/Hong_Kong/WF10_CIP046_RGAO032/1999_KtoE_GAA | 0 | 1 | 0.62691 |
| A/Waikato/120/2003_KtoE_GAG | 0 | 1 | 0.99794 |
| A/Washington/55/2015_KtoE_GAG | 0 | 1 | 0.99984 |
| A/Human/New York/PV42583/2021_KtoE_GAG | 0 | 1 | 0.99986 |
| A/Tasmania/3/2014_KtoE_GAA | 0 | 1 | 0.99975 |
| A/CastillaLaMancha/4127/2022 | 1 | 1 | 0.99982 |
| A/Singapore/30L/2007_KtoE_GAA | 0 | 1 | 0.99883 |
| A/Netherlands/12188/2022 | 1 | 1 | 0.99985 |
| A/New York/RVTNL7557587529757/2022_KtoE_GAA | 0 | 1 | 0.99983 |
| A/England/225140339/2022_KtoE_GAA | 0 | 1 | 0.99983 |
| A/guinea_fowl/New_Jersey/30701/1991_EtoK_AAA | 1 | 0 | 0.99996 |
| A/Florida/8127/2019_KtoE_GAA | 0 | 1 | 0.99977 |
| A/Texas/345/2019_EtoK_AAA | 1 | 1 | 0.99941 |
| A/Oklahoma/13468/2022 | 1 | 1 | 0.99987 |
| A/Boston/634/2009_EtoK_AAG | 1 | 1 | 0.99966 |
| A/New Mexico/44/2016 | 1 | 1 | 0.99977 |
| A/domestic_duck/Czech_Republic/1398/2023_EtoK_AAA | 1 | 0 | 0.99995 |
| A/Rio de Janeiro/26713/2022_EtoK_AAG | 1 | 1 | 0.99904 |
| A/pigeon/Wenzhou/397/2013 | 0 | 0 | 0.99978 |
| A/American_green-winged_teal/Maryland/06MD700/2006 | 0 | 0 | 0.99993 |
| A/Iowa/23/2014_EtoK_AAG | 1 | 1 | 0.99939 |
| A/Netherlands/12188/2022_KtoE_GAA | 0 | 1 | 0.99984 |
| A/Colombia/2672/2022_KtoE_GAG | 0 | 1 | 0.99985 |
| A/guineafowl/Hong_Kong/WF10_CIP046_RGAO032/1999 | 0 | 1 | 0.92865 |
| A/Chicago/YGA_04165/2013_KtoE_GAA | 0 | 1 | 0.99965 |
| A/mallard/Maryland/09OS1110/2009_EtoK_AAG | 1 | 0 | 0.99999 |
| A/Chanchamayo/FPJ01738/2022(H3N2)_KtoE_GAG | 0 | 1 | 0.99985 |
| A/New_York/20/2016 | 1 | 1 | 0.99977 |
| A/chicken/Ghana/15VIR5480-12/2015 | 0 | 0 | 0.99980 |
| A/CastillaLaMancha/4127/2022_KtoE_GAA | 0 | 1 | 0.99971 |
| A/France/ARA-HCL022211255801/2022_KtoE_GAG | 0 | 1 | 0.99985 |
| A/black vulture/Georgia/W22-723A/2022 | 0 | 0 | 0.99967 |
| A/Connecticut/31/2018_KtoE_GAG | 0 | 1 | 0.99987 |
| A/chicken/Mali/T4_180_22VIR6104-7/2022 | 0 | 0 | 0.99994 |
| A/Czech_Republic/187/2013_EtoK_AAA | 1 | 1 | 0.99973 |
| A/Texas/345/2019_EtoK_AAG | 1 | 1 | 0.99944 |
| A/blue-winged_teal/Texas/Sg-00188/2007_EtoK_AAG | 1 | 0 | 1.00000 |
| A/New_Hampshire/14/2016 | 1 | 1 | 0.99945 |
| A/Rio de Janeiro/26713/2022_EtoK_AAA | 1 | 1 | 0.99894 |
| A/Colombia/2672/2022 | 1 | 1 | 0.99986 |
| A/mallard duck/ALB/280/1978 | 0 | 0 | 0.99994 |
| A/England/128/2022_EtoK_AAG | 1 | 1 | 0.99930 |
| A/South Dakota/9110/2019_KtoE_GAG | 0 | 1 | 0.99983 |
| A/mallard/Interior_Alaska/9BM3076R0/2009 | 0 | 0 | 0.99999 |
| A/USA/55901/2011_EtoK_AAA | 1 | 1 | 0.99948 |
| A/Rheinland-Pfalz/USAFSAM-13912/2022_KtoE_GAG | 0 | 1 | 0.99988 |
| A/wild_duck/SH17-34/2008_EtoK_AAG | 1 | 0 | 0.99973 |
| A/Georgia/37/2018_KtoE_GAA | 0 | 1 | 0.99978 |
| A/Minnesota/15/2020_EtoK_AAG | 1 | 1 | 0.99951 |
| A/Singapore/GP730/2011 | 1 | 1 | 0.99969 |
| A/Sydney/25/2021_KtoE_GAA | 0 | 1 | 0.99988 |
| A/chicken/Jiangxi/19912/2013 | 0 | 0 | 0.99990 |
| A/chicken/Ghana/15VIR5480-12/2015_EtoK_AAG | 1 | 0 | 0.99534 |
| A/turkey/South Dakota/22-010135-002-original/2022_EtoK_AAA | 1 | 0 | 0.99997 |
| A/ruddy_turnstone/New_Jersey/Sg-00515/2008_EtoK_AAG | 1 | 0 | 0.99983 |
| A/Bangladesh/8016/2022_KtoE_GAG | 0 | 1 | 0.99979 |
| A/Oregon/06/2015 | 1 | 1 | 0.99983 |
| A/Melilla/3343/2022_KtoE_GAA | 0 | 1 | 0.99986 |
| A/Galicia/1455/2022 | 1 | 1 | 0.99986 |
| A/Luxembourg/LNS1177448/2022_EtoK_AAA | 1 | 1 | 0.99913 |
| A/Rheinland-Pfalz/USAFSAM-13901/2022_KtoE_GAG | 0 | 1 | 0.99982 |
| A/Netherlands/10400/2022 | 1 | 1 | 0.99984 |
| A/duck/Zhejiang/77138/2014_EtoK_AAG | 1 | 0 | 0.99273 |
| A/Michigan/85/2018 | 1 | 1 | 0.99987 |
| A/Thailand/CU-B97/2009_KtoE_GAA | 0 | 1 | 0.99981 |
| A/Singapore/GP730/2011_EtoK_AAA | 1 | 1 | 0.99972 |
| A/Alaska/63/2018_EtoK_AAA | 1 | 1 | 0.99954 |
| A/Michigan/UOM10049046862/2022 | 1 | 1 | 0.99988 |
| A/Maine/05/2015_KtoE_GAG | 0 | 1 | 0.99983 |
| A/Florida/105/2018 | 1 | 1 | 0.99969 |
| A/Iowa/14/2014_KtoE_GAA | 0 | 1 | 0.99979 |
| A/guineafowl/Hong_Kong/WF10_CIP046_RGAO032/1999_KtoE_GAG | 0 | 0 | 0.84888 |
| A/Iowa/14/2014 | 1 | 1 | 0.99979 |
| A/chicken/China/F1255/2015_EtoK_AAG | 1 | 0 | 0.99802 |
| A/California/16/2016_EtoK_AAG | 1 | 1 | 0.99906 |
| A/chicken/China/F1255/2015_EtoK_AAA | 1 | 0 | 0.99932 |
| A/Thailand/SN10452/2010_KtoE_GAG | 0 | 1 | 0.99980 |
| A/Thailand/SN10560/2011_EtoK_AAG | 1 | 1 | 0.99960 |
| A/pigeon/Wenzhou/397/2013_EtoK_AAA | 1 | 0 | 0.99884 |
| A/Delaware/09/2021_KtoE_GAG | 0 | 1 | 0.99986 |
| A/India/Nag-NIVQC126/Dec2022 | 1 | 1 | 0.99960 |
| A/Texas/7775/2018 | 1 | 1 | 0.99963 |
| A/Michigan/UOM10048462611/2022_KtoE_GAG | 0 | 1 | 0.99987 |
| A/chicken/Viet Nam/4DO3/2017_EtoK_AAG | 1 | 0 | 0.98406 |
| A/pheasant/Hong_Kong/NT458/2006_EtoK_AAA | 1 | 0 | 0.99987 |
| A/wild_waterfowl/Hong_Kong/MPL696/2011 | 0 | 0 | 0.99964 |
| A/guinea_fowl/New_Jersey/30701/1991 | 0 | 0 | 0.99997 |
| A/Thailand/SN10560/2011 | 1 | 1 | 0.99951 |
| A/Tennessee/RVTNL5465319652883/2022_KtoE_GAA | 0 | 1 | 0.99987 |
| A/Chanchamayo/FPJ01738/2022(H3N2)_KtoE_GAA | 0 | 1 | 0.99986 |
| A/California/61/2017 | 1 | 1 | 0.99930 |
| A/West Virginia/24/2015_KtoE_GAG | 0 | 1 | 0.99983 |
| A/guinea_fowl/New_Jersey/30701/1991_EtoK_AAG | 1 | 0 | 0.99996 |
| A/chicken/Shandong/lc0903/2013_EtoK_AAG | 1 | 0 | 0.62701 |
| A/chicken/Jiangxi/10875/2014 | 0 | 0 | 0.99972 |
| A/Malaysia/10081/1996_KtoE_GAG | 0 | 1 | 0.99989 |
| A/Perth/36/2015_KtoE_GAA | 0 | 1 | 0.99987 |
| A/ruddy_turnstone/New_Jersey/AI07-699/2007_EtoK_AAG | 1 | 0 | 0.99985 |
| A/England/221760284/2022 | 1 | 1 | 0.99985 |
| A/mallard duck/ALB/280/1978_EtoK_AAA | 1 | 0 | 0.99993 |
| A/chicken/Jiangxi/9530/2014_EtoK_AAG | 1 | 0 | 0.95492 |
| A/Sydney/200/2012 | 1 | 1 | 0.99981 |
| A/France/ARA-HCL022211255801/2022 | 1 | 1 | 0.99986 |
| A/Berlin/103/2022_KtoE_GAA | 0 | 1 | 0.99986 |
| A/Alaska/63/2018 | 1 | 1 | 0.99948 |
| A/New York/RVTNL7557587529757/2022_KtoE_GAG | 0 | 1 | 0.99981 |
| A/Oregon/06/2015_KtoE_GAG | 0 | 1 | 0.99983 |
| A/Rheinland-Pfalz/USAFSAM-13494/2022_KtoE_GAA | 0 | 1 | 0.99986 |
| A/England/224180743/2022_KtoE_GAG | 0 | 1 | 0.99986 |
| A/New_Hampshire/14/2016_EtoK_AAG | 1 | 1 | 0.99957 |
| A/North Dakota/25/2022_KtoE_GAA | 0 | 1 | 0.99979 |
| A/England/225160986/2022 | 1 | 1 | 0.99986 |
| A/Niger/10021/2021_KtoE_GAA | 0 | 1 | 0.99986 |
| A/Texas/13088/2022 | 1 | 1 | 0.99985 |
| A/South Africa/R04994/2022_EtoK_AAG | 1 | 1 | 0.99933 |
| A/chicken/Shanghai/2/2004_EtoK_AAG | 1 | 0 | 0.98472 |
| A/Galicia/22009514/2022 | 1 | 1 | 0.99984 |
| A/Kyrgyzstan/WRAIR1255P/2008_KtoE_GAA | 0 | 1 | 0.99979 |
| A/mallard/Ohio/11OS2091/2011_EtoK_AAG | 1 | 0 | 0.99993 |
| A/Michigan/HFHSRR005105931/2022 | 1 | 1 | 0.99988 |
| A/ruddy_turnstone/New_Jersey/Sg-00515/2008 | 0 | 0 | 0.99985 |
| A/duck/Mongolia/62/2013_EtoK_AAA | 1 | 0 | 0.99969 |
| A/environment/New Jersey/AI00-2413/2000_EtoK_AAG | 1 | 0 | 0.99989 |
| A/England/181/2010_EtoK_AAA | 1 | 1 | 0.99977 |
| A/Perth/36/2015 | 1 | 1 | 0.99987 |
| A/Netherlands/11656/2022_KtoE_GAA | 0 | 1 | 0.99971 |
| A/Minnesota/01/2017 | 1 | 1 | 0.99928 |
| A/turkey/Minnesota/22-010652-001-original/2022_EtoK_AAG | 1 | 0 | 0.99998 |
| A/Waikato/120/2003_KtoE_GAA | 0 | 1 | 0.99884 |
| A/Netherlands/10400/2022_KtoE_GAA | 0 | 1 | 0.99983 |
| A/England/225180046/2022_KtoE_GAA | 0 | 1 | 0.99987 |
| A/Texas/50/2017 | 1 | 1 | 0.99980 |
| A/New York/WC-LVD-14-052/2014_EtoK_AAA | 1 | 1 | 0.99901 |
| A/Sydney/25/2021 | 1 | 1 | 0.99988 |
| A/Wisconsin/629-S0229/2009_EtoK_AAG | 1 | 1 | 0.99966 |
| A/silkie chicken/Dongguan/1264/2014 | 0 | 0 | 0.99854 |
| A/wild_duck/SH17-34/2008_EtoK_AAA | 1 | 0 | 0.99979 |
| A/wild_duck/SH17-34/2008 | 0 | 0 | 0.99974 |
| A/California/16/2016 | 1 | 1 | 0.99855 |
| A/Sydney/200/2012_KtoE_GAA | 0 | 1 | 0.99981 |
| A/Bangladesh/8016/2022 | 1 | 1 | 0.99985 |
| A/Czech_Republic/187/2013 | 1 | 1 | 0.99971 |
| A/pigeon/Wenzhou/397/2013_EtoK_AAG | 1 | 0 | 0.99484 |
| A/mallard/Alaska/715/2005_EtoK_AAG | 1 | 0 | 0.99985 |
| A/chicken/China/F1255/2015 | 0 | 0 | 0.99983 |
| A/England/225120379/2022_KtoE_GAG | 0 | 1 | 0.99980 |
| A/Nevada/24/2017 | 1 | 1 | 0.99985 |
| A/Alabama/30/2018 | 1 | 1 | 0.99987 |
| A/Maryland/62_10D/2013 | 1 | 1 | 0.99976 |
| A/chicken/Jiangxi/10875/2014_EtoK_AAG | 1 | 0 | 0.99308 |
| A/goose/Shantou/106/2002 | 0 | 0 | 0.99991 |
| A/England/223440241/2022_EtoK_AAA | 1 | 1 | 0.99911 |
| A/New_York/52/2017 | 1 | 1 | 0.99987 |
| A/Oklahoma/13468/2022_KtoE_GAA | 0 | 1 | 0.99987 |
| A/New York/WC-LVD-14-052/2014_EtoK_AAG | 1 | 1 | 0.99918 |
| A/England/221381025/2022_KtoE_GAG | 0 | 1 | 0.99986 |
| A/duck/Mongolia/62/2013 | 0 | 0 | 0.99986 |
| A/chicken/Ghana/15VIR5480-12/2015_EtoK_AAA | 1 | 0 | 0.99822 |
| A/Michigan/124/2019 | 1 | 1 | 0.99902 |
| A/Rhode_Island/19/2018_EtoK_AAA | 1 | 1 | 0.99910 |
| A/Kyrgyzstan/WRAIR1255P/2008 | 1 | 1 | 0.99981 |
| A/CastillaLaMancha/4127/2022_KtoE_GAG | 0 | 1 | 0.99964 |
| A/England/225140339/2022_KtoE_GAG | 0 | 1 | 0.99982 |
| A/Netherlands/12188/2022_KtoE_GAG | 0 | 1 | 0.99983 |
| A/Ethiopia/2611/2022_EtoK_AAG | 1 | 1 | 0.99963 |
| A/pheasant/Hong_Kong/NT458/2006_EtoK_AAG | 1 | 0 | 0.99987 |
| A/New York/WC-LVD-12-043/2012 | 1 | 1 | 0.99975 |
| A/England/181/2010 | 1 | 1 | 0.99976 |
| A/red knot/Delaware Bay/598/2017_EtoK_AAG | 1 | 0 | 0.99975 |
| A/mallard/Maryland/09OS1110/2009 | 0 | 0 | 1.00000 |
| A/Nicaragua/7965_13/2016 | 1 | 1 | 0.99981 |
| A/Berlin/103/2022 | 1 | 1 | 0.99987 |
| A/chicken/Shanghai/2/2004_EtoK_AAA | 1 | 0 | 0.98939 |
| A/Minnesota/09/2023 | 1 | 1 | 0.99983 |
| A/New York/WC-LVD-11-003/2011_KtoE_GAA | 0 | 1 | 0.99985 |
| A/New York/WC-LVD-11-003/2011 | 1 | 1 | 0.99985 |
| A/Japanese Quail/Vietnam/4/2009_EtoK_AAA | 1 | 0 | 0.94806 |
| A/Brisbane/79/2015_KtoE_GAA | 0 | 1 | 0.99982 |
| A/Delaware/36/2014 | 1 | 1 | 0.99972 |
| A/South Dakota/9110/2019 | 1 | 1 | 0.99983 |
| A/chicken/England/071631/2021_EtoK_AAA | 1 | 0 | 0.99992 |
| A/environment/New Jersey/AI00-2413/2000_EtoK_AAA | 1 | 0 | 0.99990 |
| A/Bangkok/INS479/2010 | 1 | 1 | 0.99970 |
| A/Maryland/10/2019_KtoE_GAG | 0 | 1 | 0.99980 |
| A/New York/A-WC-LVD-16-013/2016_EtoK_AAA | 1 | 1 | 0.99935 |
| A/Tennessee/RVTNL5465319652883/2022_KtoE_GAG | 0 | 1 | 0.99986 |
| A/Delaware/09/2021 | 1 | 1 | 0.99986 |
| A/mallard/Idaho/AH0008589/2015 | 0 | 0 | 0.99993 |
| A/Michigan/UOM10049046862/2022_KtoE_GAG | 0 | 1 | 0.99986 |
| A/Galicia/1455/2022_KtoE_GAG | 0 | 1 | 0.99986 |
| A/New_York/20/2016_KtoE_GAA | 0 | 1 | 0.99977 |
| A/turkey/Minnesota/22-011793-002-original/2022_EtoK_AAG | 1 | 0 | 0.99998 |
| A/Moscow/127-88V/2021_KtoE_GAG | 0 | 1 | 0.99987 |
| A/chicken/Shandong/11/2011_EtoK_AAG | 1 | 0 | 0.99843 |
| A/mallard/Sweden/58369/2006_EtoK_AAG | 1 | 0 | 0.99985 |
| A/England/221760284/2022_KtoE_GAG | 0 | 1 | 0.99985 |
| A/chicken/Mali/T4_180_22VIR6104-7/2022_EtoK_AAA | 1 | 0 | 0.99993 |
| A/Minnesota/01/2017_EtoK_AAG | 1 | 1 | 0.99946 |
| A/South Africa/R04994/2022 | 1 | 1 | 0.99911 |
| A/Houston/2H/2009_EtoK_AAG | 1 | 1 | 0.99966 |
| A/Netherlands/10400/2022_KtoE_GAG | 0 | 1 | 0.99983 |
| A/England/225180046/2022 | 1 | 1 | 0.99987 |
| A/snow goose/Kentucky/15-012967-2/2015_EtoK_AAG | 1 | 0 | 0.99994 |
| A/Colorado/01/2022_KtoE_GAA | 0 | 1 | 0.99985 |
| A/Michigan/124/2019_EtoK_AAG | 1 | 1 | 0.99928 |
| A/England/224180743/2022_KtoE_GAA | 0 | 1 | 0.99986 |
| A/environment/New Jersey/AI00-2413/2000 | 0 | 0 | 0.99989 |
| A/USA/55901/2011_EtoK_AAG | 1 | 1 | 0.99952 |
| A/Goias /IAL/C10304/2022 | 1 | 1 | 0.99903 |
| A/ruddy_turnstone/New_Jersey/AI07-699/2007 | 0 | 0 | 0.99987 |
| A/Nicaragua/7965_13/2016_KtoE_GAG | 0 | 1 | 0.99980 |
| A/Tennessee/RVTN111902002/2022_KtoE_GAA | 0 | 1 | 0.99987 |
| A/Washington/74/2018_KtoE_GAA | 0 | 1 | 0.99987 |
| A/chicken/Shandong/11/2011 | 0 | 0 | 0.99986 |
| A/mallard/Alaska/715/2005_EtoK_AAA | 1 | 0 | 0.99986 |
| A/Netherlands/11476/2022_EtoK_AAA | 1 | 1 | 0.99908 |
| A/England/223440241/2022_EtoK_AAG | 1 | 1 | 0.99918 |
| A/France/ARA-HCL022211255801/2022_KtoE_GAA | 0 | 1 | 0.99986 |
| A/Tasmania/3/2014 | 1 | 1 | 0.99976 |
| A/Colombia/2672/2022_KtoE_GAA | 0 | 1 | 0.99986 |
| A/Wisconsin/629-S0229/2009 | 1 | 1 | 0.99961 |
| A/HaNoi/HN1004/2003_KtoE_GAA | 0 | 1 | 0.99984 |
| A/Melilla/3343/2022 | 1 | 1 | 0.99986 |
| A/USA/55901/2011 | 1 | 1 | 0.99944 |
| A/Nicaragua/6933_28/2017 | 1 | 1 | 0.99987 |
| A/India/Nag-NIVQC126/Dec2022_EtoK_AAA | 1 | 1 | 0.99963 |
| A/England/225160986/2022_KtoE_GAG | 0 | 1 | 0.99984 |
| A/Nicaragua/7302_09/2013 | 1 | 1 | 0.99967 |
| A/Rheinland-Pfalz/USAFSAM-13901/2022_KtoE_GAA | 0 | 1 | 0.99983 |
| A/California/16/2016_EtoK_AAA | 1 | 1 | 0.99892 |
| A/New Mexico/44/2016_KtoE_GAA | 0 | 1 | 0.99977 |
| A/South Korea/8829/2019 | 1 | 1 | 0.99979 |
| A/chicken/Shandong/lc0903/2013 | 0 | 0 | 0.99918 |
| A/Luxembourg/LNS1177448/2022_EtoK_AAG | 1 | 1 | 0.99919 |
| A/Nicaragua/7302_09/2013_EtoK_AAG | 1 | 1 | 0.99973 |
| A/Houston/2H/2009 | 1 | 1 | 0.99962 |
| A/mallard/Alberta/232/1994_EtoK_AAG | 1 | 0 | 0.99984 |
| A/wild_waterfowl/Hong_Kong/MPL696/2011_EtoK_AAG | 1 | 0 | 0.99226 |
| A/Galicia/1455/2022_KtoE_GAA | 0 | 1 | 0.99986 |
| A/chicken/Shandong/11/2011_EtoK_AAA | 1 | 0 | 0.99941 |
| A/Rheinland-Pfalz/USAFSAM-13912/2022_KtoE_GAA | 0 | 1 | 0.99988 |
| A/Alabama/30/2018_KtoE_GAA | 0 | 1 | 0.99987 |
| A/Michigan/UOM10042700269/2021_KtoE_GAG | 0 | 1 | 0.99986 |
| A/Delaware/36/2014_KtoE_GAA | 0 | 1 | 0.99972 |
| A/chicken/England/071631/2021_EtoK_AAG | 1 | 0 | 0.99992 |
| A/Texas/USAFSAM-13474/2022_KtoE_GAA | 0 | 1 | 0.99986 |
| A/Texas/USAFSAM-13474/2022 | 1 | 1 | 0.99986 |
| A/chicken/England/071631/2021 | 0 | 0 | 0.99992 |
| A/California/36/2016 | 1 | 1 | 0.99984 |
| A/Netherlands/11476/2022_EtoK_AAG | 1 | 1 | 0.99913 |
| A/England/222000262/2022 | 1 | 1 | 0.99917 |
| A/mallard/Interior_Alaska/9BM3076R0/2009_EtoK_AAG | 1 | 0 | 0.99997 |
| A/Delaware/09/2021_KtoE_GAA | 0 | 1 | 0.99986 |
| A/England/128/2022 | 1 | 1 | 0.99904 |
| A/New York/WC-LVD-11-003/2011_KtoE_GAG | 0 | 1 | 0.99984 |
| A/Minnesota/15/2020 | 1 | 1 | 0.99943 |
| A/environment/Bangladesh/15114/2012_EtoK_AAA | 1 | 0 | 0.99992 |
| A/mallard/Ohio/11OS2091/2011 | 0 | 0 | 0.99995 |
| A/black vulture/Georgia/W22-723A/2022_EtoK_AAG | 1 | 0 | 0.99953 |
| A/Rheinland-Pfalz/USAFSAM-13912/2022 | 1 | 1 | 0.99988 |
| A/Bangladesh/8016/2022_KtoE_GAA | 0 | 1 | 0.99983 |
| A/New York/462/2005_KtoE_GAG | 0 | 1 | 0.99959 |
| A/Berlin/103/2022_KtoE_GAG | 0 | 1 | 0.99985 |
| A/Tasmania/3/2014_KtoE_GAG | 0 | 1 | 0.99974 |
| A/mallard/Maryland/09OS1110/2009_EtoK_AAA | 1 | 0 | 0.99999 |
| A/South Africa/R04994/2022_EtoK_AAA | 1 | 1 | 0.99928 |
| A/Nicaragua/6933_28/2017_KtoE_GAG | 0 | 1 | 0.99986 |
| A/mallard/Alberta/64/2000 | 0 | 0 | 1.00000 |
| A/Texas/345/2019 | 1 | 1 | 0.99933 |
| A/mallard duck/Netherlands/19/2009_EtoK_AAA | 1 | 0 | 0.99957 |
| A/New_Hampshire/14/2016_EtoK_AAA | 1 | 1 | 0.99953 |
| A/red knot/Delaware Bay/598/2017_EtoK_AAA | 1 | 0 | 0.99975 |
| A/blue-winged_teal/Texas/Sg-00188/2007 | 0 | 0 | 1.00000 |
| A/chicken/Mali/T4_180_22VIR6104-7/2022_EtoK_AAG | 1 | 0 | 0.99992 |
| A/mallard/Alberta/64/2000_EtoK_AAA | 1 | 0 | 1.00000 |
| A/Florida/105/2018_EtoK_AAG | 1 | 1 | 0.99973 |
| A/Rheinland-Pfalz/USAFSAM-13494/2022 | 1 | 1 | 0.99986 |
| A/West Virginia/24/2015_KtoE_GAA | 0 | 1 | 0.99984 |
| A/Dakar/53/2021_EtoK_AAA | 1 | 1 | 0.99967 |
| A/Maine/05/2015 | 1 | 1 | 0.99983 |
| A/Michigan/UOM10048602233/2022_KtoE_GAG | 0 | 1 | 0.99988 |
| A/Boston/606/2009_EtoK_AAG | 1 | 1 | 0.99966 |
| A/Michigan/UOM10045253733/2022_KtoE_GAG | 0 | 1 | 0.99988 |
| A/environment/Guizhou/2/2009_EtoK_AAG | 1 | 0 | 0.99099 |
| A/Luxembourg/LNS1177448/2022 | 1 | 1 | 0.99888 |
| A/chicken/Shandong/lc0903/2013_EtoK_AAA | 1 | 0 | 0.91224 |
| A/Maryland/10/2019 | 1 | 1 | 0.99980 |
| A/chicken/Jiangxi/9530/2014 | 0 | 0 | 0.99942 |
| A/Izhevsk/RII-MH45756S/2021_KtoE_GAA | 0 | 1 | 0.99986 |
| A/North Dakota/25/2022 | 1 | 1 | 0.99984 |
| A/mallard/Idaho/AH0008589/2015_EtoK_AAA | 1 | 0 | 0.99993 |
| A/Michigan/UOM10048602233/2022 | 1 | 1 | 0.99988 |
| A/Saint-Petersburg/RII-8628S/2023 | 1 | 1 | 0.99948 |
| A/mallard/Interior_Alaska/9BM3076R0/2009_EtoK_AAA | 1 | 0 | 0.99996 |
| A/Thailand/SN10452/2010 | 1 | 1 | 0.99981 |
| A/Alabama/30/2018_KtoE_GAG | 0 | 1 | 0.99987 |
| A/Michigan/UOM10048462611/2022_KtoE_GAA | 0 | 1 | 0.99987 |
| A/Brisbane/79/2015 | 1 | 1 | 0.99982 |
| A/South Korea/8829/2019_KtoE_GAG | 0 | 1 | 0.99980 |
| A/Michigan/UOM10045253733/2022 | 1 | 1 | 0.99988 |
| A/Goias /IAL/C10304/2022_EtoK_AAG | 1 | 1 | 0.99927 |
| A/Bangkok/INS485/2010_EtoK_AAA | 1 | 1 | 0.99975 |
| A/Sydney/644/2022 | 1 | 1 | 0.99949 |
| A/environment/Guizhou/2/2009 | 0 | 0 | 0.99425 |
| A/Sydney/200/2012_KtoE_GAG | 0 | 1 | 0.99980 |
| A/Shiga/3/2009 | 1 | 1 | 0.99854 |
| A/Georgia/37/2018_KtoE_GAG | 0 | 1 | 0.99979 |
| A/blue-winged teal/New Brunswick/03756/2009_EtoK_AAA | 1 | 0 | 0.99995 |
| A/Michigan/UOM10042700269/2021_KtoE_GAA | 0 | 1 | 0.99986 |
| A/Michigan/UOM10043145906/2021_KtoE_GAA | 0 | 1 | 0.99986 |
| A/Berlin/86/2022_KtoE_GAG | 0 | 1 | 0.99987 |
| A/England/225100947/2022_KtoE_GAG | 0 | 1 | 0.99985 |
| A/Waikato/120/2003 | 1 | 1 | 0.99946 |
| A/England/225120379/2022 | 1 | 1 | 0.99984 |
| A/Michigan/UOM10043145906/2021 | 1 | 1 | 0.99986 |
| A/snow goose/Kentucky/15-012967-2/2015_EtoK_AAA | 1 | 0 | 0.99994 |
| A/Texas/50/2017_KtoE_GAA | 0 | 1 | 0.99980 |
| A/Thailand/CU-B97/2009 | 1 | 1 | 0.99981 |
| A/Boston/606/2009_EtoK_AAA | 1 | 1 | 0.99964 |
| A/Michigan/UOM10043145906/2021_KtoE_GAG | 0 | 1 | 0.99986 |
| A/Alaska/18/2018_KtoE_GAA | 0 | 1 | 0.99988 |
| A/England/225140339/2022 | 1 | 1 | 0.99985 |
| A/Izhevsk/RII-MH45756S/2021 | 1 | 1 | 0.99986 |
| A/Galicia/22009514/2022_KtoE_GAA | 0 | 1 | 0.99982 |
| A/North Dakota/25/2022_KtoE_GAG | 0 | 1 | 0.99975 |
| A/Minnesota/09/2023_KtoE_GAG | 0 | 1 | 0.99983 |
| A/England/221320672/2022 | 1 | 1 | 0.99988 |
| A/Netherlands/11656/2022_KtoE_GAG | 0 | 1 | 0.99963 |
| A/Texas/USAFSAM-13474/2022_KtoE_GAG | 0 | 1 | 0.99986 |
| A/New_York/20/2016_KtoE_GAG | 0 | 1 | 0.99975 |
| A/goose/Shantou/106/2002_EtoK_AAA | 1 | 0 | 0.99988 |
| A/blue-winged_teal/Texas/Sg-00188/2007_EtoK_AAA | 1 | 0 | 1.00000 |
| A/Dakar/53/2021_EtoK_AAG | 1 | 1 | 0.99968 |
| A/New York/WC-LVD-12-043/2012_EtoK_AAA | 1 | 1 | 0.99978 |
| A/Tennessee/RVTN111902002/2022 | 1 | 1 | 0.99987 |
| A/mallard/Maryland/11OS3701/2011_EtoK_AAA | 1 | 0 | 0.99997 |
| A/Tumbes/FPT02312/2022(H3N2)_KtoE_GAA | 0 | 1 | 0.99986 |
| A/Iowa/23/2014 | 1 | 1 | 0.99914 |
| A/Perth/36/2015_KtoE_GAG | 0 | 1 | 0.99986 |
| A/India/Nag-NIVQC126/Dec2022_EtoK_AAG | 1 | 1 | 0.99965 |
| A/Colorado/01/2022_KtoE_GAG | 0 | 1 | 0.99985 |
| A/Human/New York City/PV42586/2021 | 1 | 1 | 0.99986 |
| A/New York/462/2005_KtoE_GAA | 0 | 1 | 0.99968 |
| A/bufflehead/Alaska/594/2014 | 0 | 0 | 0.99991 |
| A/Kabul/1514A01305430N/2013 | 1 | 1 | 0.99978 |
| A/Bangkok/INS479/2010_EtoK_AAG | 1 | 1 | 0.99973 |
| A/Human/New York/PV42583/2021 | 1 | 1 | 0.99986 |
| A/duck/Zhejiang/77138/2014_EtoK_AAA | 1 | 0 | 0.99587 |
| A/Boston/634/2009 | 1 | 1 | 0.99958 |
| A/ruddy_turnstone/New_Jersey/AI07-699/2007_EtoK_AAA | 1 | 0 | 0.99985 |
| A/England/222000262/2022_EtoK_AAG | 1 | 1 | 0.99932 |
| A/Kentucky/UR06-0128/2007_KtoE_GAG | 0 | 1 | 0.99946 |
| A/Wyoming/03/2019_EtoK_AAA | 1 | 1 | 0.99962 |
| A/HaNoi/HN1004/2003 | 1 | 1 | 0.99984 |
| A/Maryland/10/2019_KtoE_GAA | 0 | 1 | 0.99979 |
| A/Texas/50/2017_KtoE_GAG | 0 | 1 | 0.99979 |
| A/blue-winged teal/New Brunswick/03756/2009_EtoK_AAG | 1 | 0 | 0.99996 |
| A/Alabama/06/2022_KtoE_GAG | 0 | 1 | 0.99986 |
| A/Goias /IAL/C10304/2022_EtoK_AAA | 1 | 1 | 0.99920 |
| A/Izhevsk/RII-MH45756S/2021_KtoE_GAG | 0 | 1 | 0.99986 |
| A/Niger/10021/2021 | 1 | 1 | 0.99986 |
| A/Michigan/UOM10045473306/2022_KtoE_GAA | 0 | 1 | 0.99987 |
| A/New York/781/1993 | 1 | 1 | 0.99990 |
| A/England/221900400/2022_KtoE_GAA | 0 | 1 | 0.99986 |
| A/Oklahoma/13468/2022_KtoE_GAG | 0 | 1 | 0.99987 |
| A/Kabul/1514A01305430N/2013_KtoE_GAG | 0 | 1 | 0.99976 |
| A/Maryland/62_10D/2013_KtoE_GAA | 0 | 1 | 0.99976 |
| A/mallard duck/Netherlands/19/2009 | 0 | 0 | 0.99963 |
| A/Niger/10021/2021_KtoE_GAG | 0 | 1 | 0.99985 |
| A/New Mexico/44/2016_KtoE_GAG | 0 | 1 | 0.99975 |
| A/Berlin/223/2022_KtoE_GAG | 0 | 1 | 0.99983 |
| A/California/36/2016_KtoE_GAG | 0 | 1 | 0.99984 |
| A/mallard/Idaho/AH0008589/2015_EtoK_AAG | 1 | 0 | 0.99993 |
| A/chicken/Viet Nam/4DO3/2017 | 0 | 0 | 0.99877 |
| A/chicken/Shanghai/2/2004 | 0 | 0 | 0.99608 |
| A/mallard/Minnesota/Sg-00701/2008_EtoK_AAG | 1 | 0 | 0.99988 |
| A/Nicaragua/6933_28/2017_KtoE_GAA | 0 | 1 | 0.99987 |
| A/England/221760284/2022_KtoE_GAA | 0 | 1 | 0.99985 |
| A/mallard duck/ALB/280/1978_EtoK_AAG | 1 | 0 | 0.99993 |
| A/Melilla/3343/2022_KtoE_GAG | 0 | 1 | 0.99986 |
| A/Tumbes/FPT02312/2022(H3N2) | 1 | 1 | 0.99987 |
| A/Netherlands/11441/2022_KtoE_GAG | 0 | 1 | 0.99977 |
| A/California/61/2017_EtoK_AAG | 1 | 1 | 0.99944 |
| A/Washington/74/2018 | 1 | 1 | 0.99988 |
| A/mallard/Sweden/58369/2006 | 0 | 0 | 0.99985 |
| A/Sydney/644/2022_EtoK_AAG | 1 | 1 | 0.99956 |
| A/England/221900400/2022_KtoE_GAG | 0 | 1 | 0.99986 |
| A/New York/781/1993_KtoE_GAG | 0 | 1 | 0.99990 |
| A/Washington/55/2015 | 1 | 1 | 0.99984 |
| A/wild_waterfowl/Hong_Kong/MPL696/2011_EtoK_AAA | 1 | 0 | 0.99789 |
| A/West Virginia/24/2015 | 1 | 1 | 0.99984 |
| A/Michigan/UOM10049046862/2022_KtoE_GAA | 0 | 1 | 0.99987 |
| A/Texas/7775/2018_EtoK_AAA | 1 | 1 | 0.99965 |
| A/blue-winged teal/New Brunswick/03756/2009 | 0 | 0 | 0.99997 |
| A/mallard/Alberta/64/2000_EtoK_AAG | 1 | 0 | 1.00000 |
| A/Bangkok/INS485/2010 | 1 | 1 | 0.99974 |
| A/Sao Paulo/IAL/C8561/2021 | 1 | 1 | 0.99987 |
| A/Sao Paulo/IAL/C8561/2021_KtoE_GAA | 0 | 1 | 0.99986 |
| A/environment/Guizhou/2/2009_EtoK_AAA | 1 | 0 | 0.99400 |
| A/Czech_Republic/187/2013_EtoK_AAG | 1 | 1 | 0.99974 |
| A/Nevada/24/2017_KtoE_GAA | 0 | 1 | 0.99985 |
| A/England/221900400/2022 | 1 | 1 | 0.99986 |
| A/Wyoming/03/2019 | 1 | 1 | 0.99956 |
| A/mallard duck/Netherlands/19/2009_EtoK_AAG | 1 | 0 | 0.99951 |
| A/Connecticut/31/2018_KtoE_GAA | 0 | 1 | 0.99987 |
| A/Saint-Petersburg/RII-8628S/2023_EtoK_AAA | 1 | 1 | 0.99956 |
| A/Michigan/UOM10045473306/2022 | 1 | 1 | 0.99987 |
| A/mallard/Minnesota/Sg-00701/2008 | 0 | 0 | 0.99989 |
| A/Moscow/127-88V/2021_KtoE_GAA | 0 | 1 | 0.99987 |
| A/Shiga/3/2009_EtoK_AAA | 1 | 1 | 0.99895 |
| A/Berlin/86/2022 | 1 | 1 | 0.99987 |
| A/Minnesota/01/2017_EtoK_AAA | 1 | 1 | 0.99940 |
| A/England/221381025/2022_KtoE_GAA | 0 | 1 | 0.99986 |
| A/New_York/52/2017_KtoE_GAA | 0 | 1 | 0.99987 |
| A/turkey/South Dakota/22-010135-002-original/2022 | 0 | 0 | 0.99998 |
| A/duck/Pennsylvania/22-014337-007-original/2022 | 0 | 0 | 0.99970 |
| A/Saint-Petersburg/RII-8628S/2023_EtoK_AAG | 1 | 1 | 0.99958 |
| A/Tennessee/RVTNL5465319652883/2022 | 1 | 1 | 0.99987 |
| A/turkey/Minnesota/22-011793-002-original/2022 | 0 | 0 | 0.99998 |
| A/red knot/Delaware Bay/598/2017 | 0 | 0 | 0.99987 |
| A/silkie chicken/Dongguan/1264/2014_EtoK_AAG | 1 | 0 | 0.58364 |
| A/Florida/105/2018_EtoK_AAA | 1 | 1 | 0.99971 |
| A/Nevada/24/2017_KtoE_GAG | 0 | 1 | 0.99985 |
| A/mallard/Alberta/232/1994_EtoK_AAA | 1 | 0 | 0.99989 |
| A/Wyoming/03/2019_EtoK_AAG | 1 | 1 | 0.99964 |
| A/England/225100947/2022 | 1 | 1 | 0.99987 |
| A/Texas/90/2017 | 1 | 1 | 0.99985 |
| A/Human/New York/PV42583/2021_KtoE_GAA | 0 | 1 | 0.99986 |
| A/Shiga/3/2009_EtoK_AAG | 1 | 1 | 0.99905 |
| A/Netherlands/11441/2022_KtoE_GAA | 0 | 1 | 0.99981 |
| A/Sydney/644/2022_EtoK_AAA | 1 | 1 | 0.99954 |
| A/mallard/Maryland/11OS3603/2011_EtoK_AAA | 1 | 0 | 0.99997 |
| A/Rio de Janeiro/26713/2022 | 1 | 1 | 0.99864 |
| A/Michigan/85/2018_KtoE_GAG | 0 | 1 | 0.99987 |
| A/American_green-winged_teal/Maryland/06MD700/2006_EtoK_AAA | 1 | 0 | 0.99988 |
| A/Delaware/36/2014_KtoE_GAG | 0 | 1 | 0.99969 |
| A/Singapore/30L/2007 | 1 | 1 | 0.99895 |
| A/Rheinland-Pfalz/USAFSAM-13494/2022_KtoE_GAG | 0 | 1 | 0.99986 |
| A/California/61/2017_EtoK_AAA | 1 | 1 | 0.99941 |
| A/England/223440241/2022 | 1 | 1 | 0.99887 |
| A/England/221320672/2022_KtoE_GAG | 0 | 1 | 0.99988 |
| A/Chicago/YGA_04165/2013 | 1 | 1 | 0.99977 |
| A/Ethiopia/2611/2022_EtoK_AAA | 1 | 1 | 0.99962 |
| A/Delaware/24/2022 | 1 | 1 | 0.99986 |
| A/mallard/Alberta/232/1994 | 0 | 0 | 0.99993 |
| A/Singapore/30L/2007_KtoE_GAG | 0 | 1 | 0.99897 |
| A/Minnesota/09/2023_KtoE_GAA | 0 | 1 | 0.99983 |
| A/Chanchamayo/FPJ01738/2022(H3N2) | 1 | 1 | 0.99987 |
| A/snow goose/Kentucky/15-012967-2/2015 | 0 | 0 | 0.99997 |
| A/chicken/Viet Nam/4DO3/2017_EtoK_AAA | 1 | 0 | 0.99043 |
| A/Rheinland-Pfalz/USAFSAM-13901/2022 | 1 | 1 | 0.99984 |
| A/duck/Mongolia/62/2013_EtoK_AAG | 1 | 0 | 0.99958 |
| A/Brisbane/79/2015_KtoE_GAG | 0 | 1 | 0.99981 |
| A/Rhode_Island/19/2018_EtoK_AAG | 1 | 1 | 0.99917 |
| A/Minnesota/15/2020_EtoK_AAA | 1 | 1 | 0.99949 |
| A/mallard/Minnesota/Sg-00701/2008_EtoK_AAA | 1 | 0 | 0.99987 |
| A/turkey/Minnesota/22-010652-001-original/2022 | 0 | 0 | 0.99998 |
| A/Malaysia/10081/1996 | 1 | 1 | 0.99990 |
| A/ruddy_turnstone/New_Jersey/Sg-00515/2008_EtoK_AAA | 1 | 0 | 0.99981 |
| A/Texas/13088/2022_KtoE_GAG | 0 | 1 | 0.99985 |
| A/South Dakota/9110/2019_KtoE_GAA | 0 | 1 | 0.99983 |
| A/Kabul/1514A01305430N/2013_KtoE_GAA | 0 | 1 | 0.99978 |
| A/Texas/90/2017_KtoE_GAG | 0 | 1 | 0.99981 |
| A/Michigan/HFHSRR005105931/2022_KtoE_GAG | 0 | 1 | 0.99987 |
| A/mallard/Sweden/58369/2006_EtoK_AAA | 1 | 0 | 0.99986 |
| A/turkey/Minnesota/22-011793-002-original/2022_EtoK_AAA | 1 | 0 | 0.99997 |
| A/Kyrgyzstan/WRAIR1255P/2008_KtoE_GAG | 0 | 1 | 0.99977 |
| A/Human/New York City/PV60760/2022 | 1 | 1 | 0.99986 |
| A/Colorado/01/2022 | 1 | 1 | 0.99985 |
| A/mallard duck/Netherlands/43/2011_EtoK_AAG | 1 | 0 | 0.99996 |
| A/Netherlands/11441/2022 | 1 | 1 | 0.99985 |
| A/silkie chicken/Dongguan/1264/2014_EtoK_AAA | 1 | 0 | 0.88531 |
| A/mallard duck/Netherlands/12/2012_EtoK_AAA | 1 | 0 | 0.99875 |
| A/England/225100947/2022_KtoE_GAA | 0 | 1 | 0.99986 |
| A/Alaska/63/2018_EtoK_AAG | 1 | 1 | 0.99956 |
| A/Oregon/06/2015_KtoE_GAA | 0 | 1 | 0.99983 |
| A/bufflehead/Alaska/594/2014_EtoK_AAA | 1 | 0 | 0.99991 |
| A/Florida/8127/2019 | 1 | 1 | 0.99978 |
| A/New York/462/2005 | 1 | 1 | 0.99974 |
| A/Dakar/53/2021 | 1 | 1 | 0.99962 |
| A/mallard/Alaska/715/2005 | 0 | 0 | 0.99988 |
| A/England/181/2010_EtoK_AAG | 1 | 1 | 0.99978 |
| A/duck/Zhejiang/77138/2014 | 0 | 0 | 0.99588 |
| A/England/128/2022_EtoK_AAA | 1 | 1 | 0.99923 |
| A/England/224180743/2022 | 1 | 1 | 0.99986 |
| A/black vulture/Georgia/W22-723A/2022_EtoK_AAA | 1 | 0 | 0.99953 |
| A/environment/Bangladesh/15114/2012 | 0 | 0 | 0.99990 |
| A/England/225120379/2022_KtoE_GAA | 0 | 1 | 0.99982 |
| A/Tennessee/RVTN111902002/2022_KtoE_GAG | 0 | 1 | 0.99987 |
| A/Human/New York City/PV42586/2021_KtoE_GAA | 0 | 1 | 0.99986 |
| A/Siena/14/1995 | 1 | 1 | 0.99940 |
| A/Japanese Quail/Vietnam/4/2009 | 0 | 0 | 0.99729 |
| A/Bangkok/INS479/2010_EtoK_AAA | 1 | 1 | 0.99972 |
| A/Delaware/24/2022_KtoE_GAA | 0 | 1 | 0.99986 |
| A/Human/New York City/PV60760/2022_KtoE_GAG | 0 | 1 | 0.99985 |
| A/chicken/Jiangxi/19912/2013_EtoK_AAA | 1 | 0 | 0.99967 |
| A/Japanese Quail/Vietnam/4/2009_EtoK_AAG | 1 | 0 | 0.92187 |
| A/Alabama/06/2022_KtoE_GAA | 0 | 1 | 0.99986 |
| A/mallard/Maryland/11OS3701/2011 | 0 | 0 | 0.99999 |
| A/Michigan/HFHSRR005105931/2022_KtoE_GAA | 0 | 1 | 0.99988 |
| A/Bangkok/INS485/2010_EtoK_AAG | 1 | 1 | 0.99976 |
| A/Florida/8127/2019_KtoE_GAG | 0 | 1 | 0.99978 |
| A/Malaysia/10081/1996_KtoE_GAA | 0 | 1 | 0.99990 |
| A/goose/Shantou/106/2002_EtoK_AAG | 1 | 0 | 0.99986 |
| A/Georgia/37/2018 | 1 | 1 | 0.99979 |
| A/mallard duck/Netherlands/12/2012 | 0 | 0 | 0.99948 |
