# Supplementary figures and images for: Deep learning predicts potential reassortments of avian H5N1 with human influenza viruses
Source: Natl Sci Rev. 2025 Sep 17;12(12):nwaf396. doi: 10.1093/nsr/nwaf396 (PMC12707066; doi:10.1093/nsr/nwaf396)

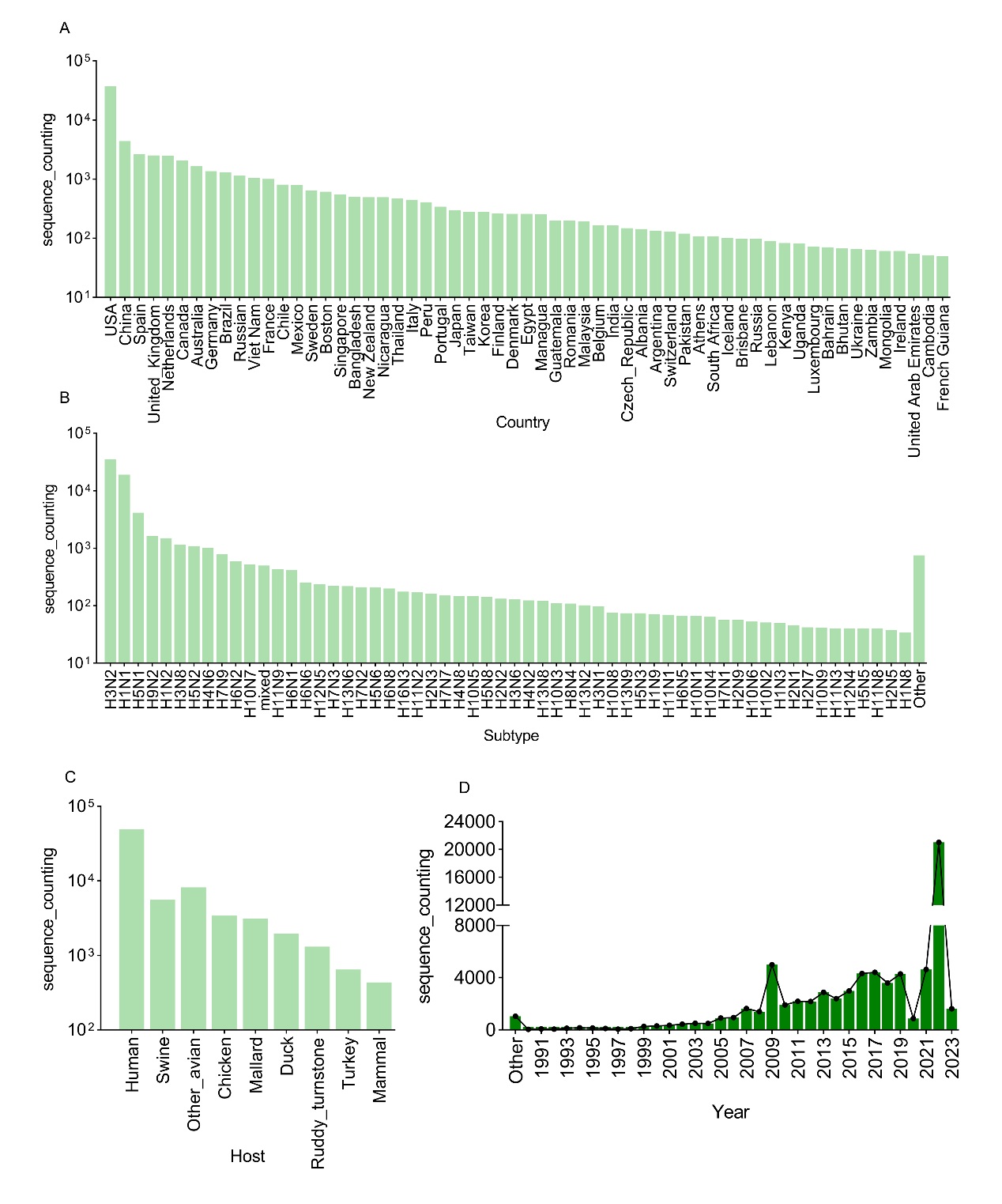

Supplement: nwaf396_Supplemental_Files [file nwaf396_supplemental_files.zip › Supplementary Figure 1.tif]

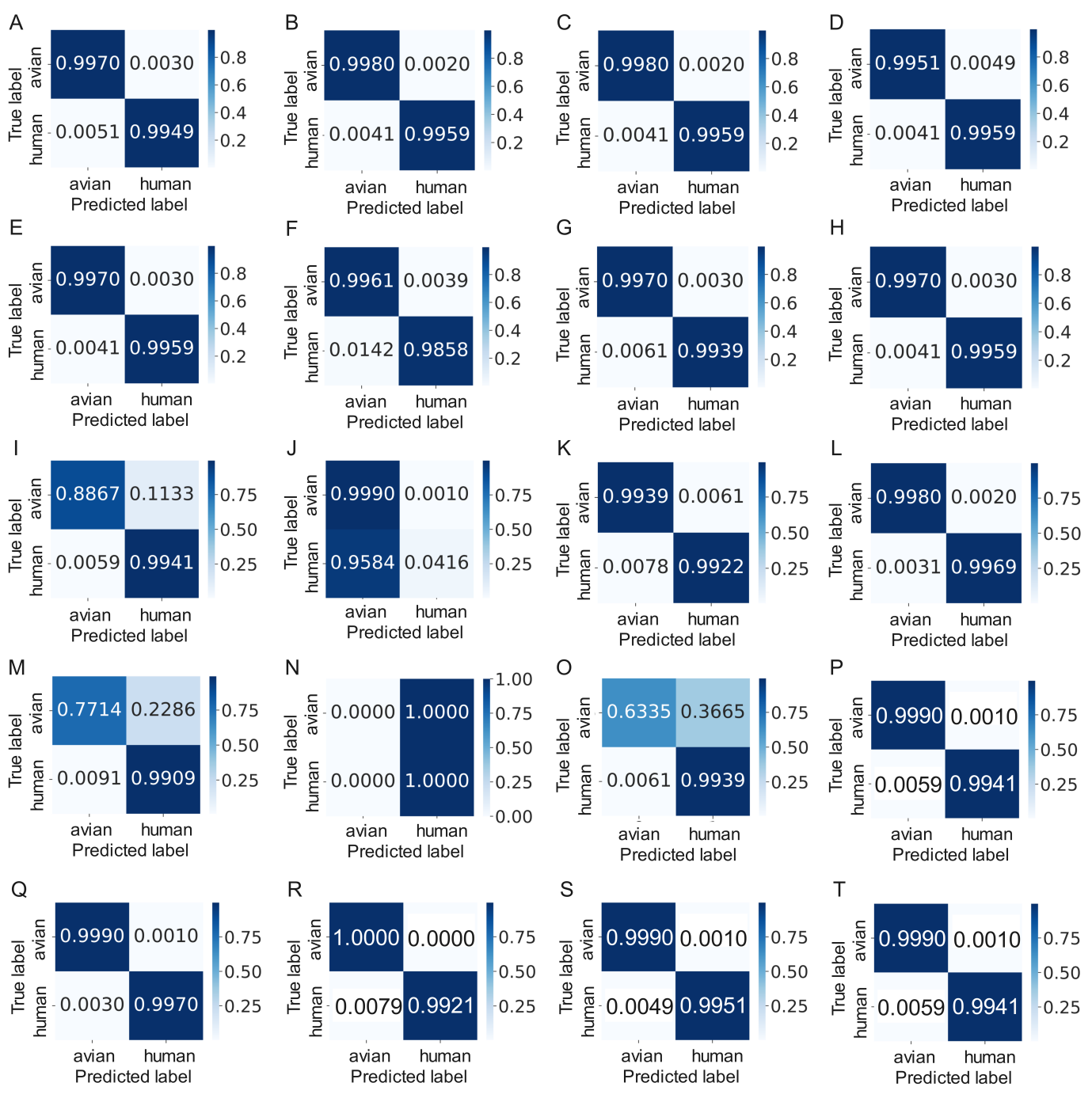

Supplement: nwaf396_Supplemental_Files [file nwaf396_supplemental_files.zip › Supplementary Figure 10.tif]

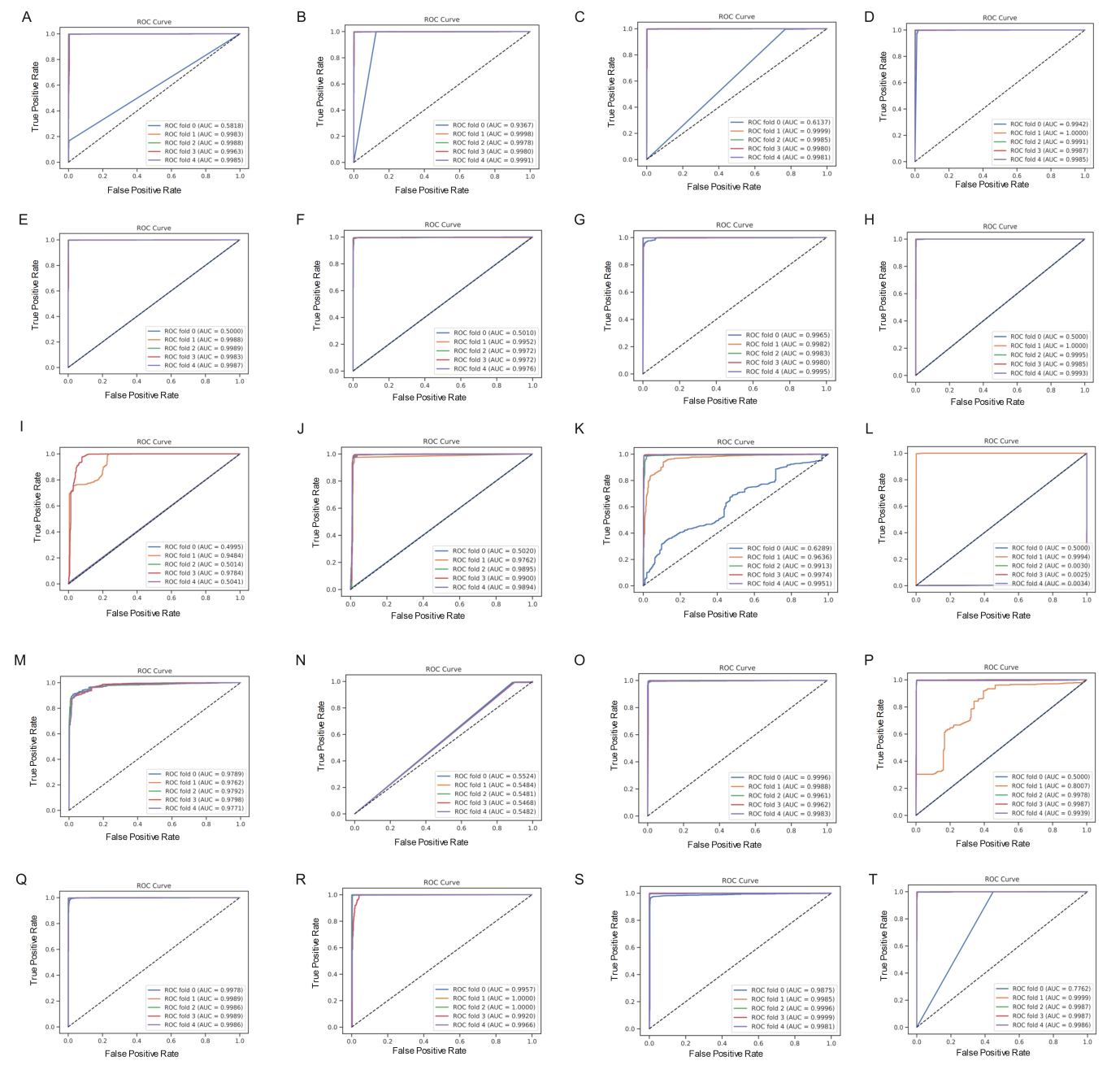

Supplement: nwaf396_Supplemental_Files [file nwaf396_supplemental_files.zip › Supplementary Figure 11.tif]

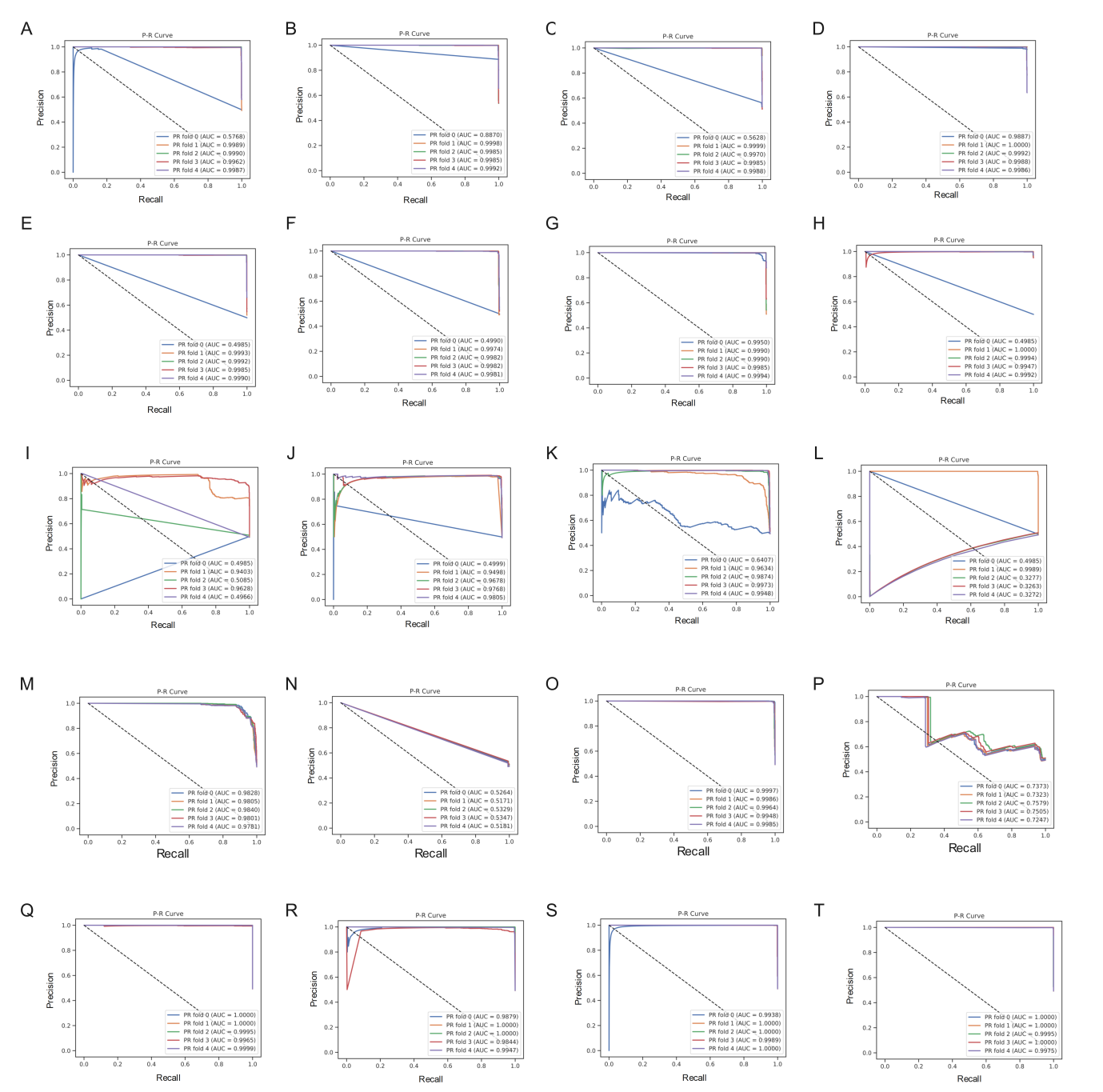

Supplement: nwaf396_Supplemental_Files [file nwaf396_supplemental_files.zip › Supplementary Figure 12.tif]

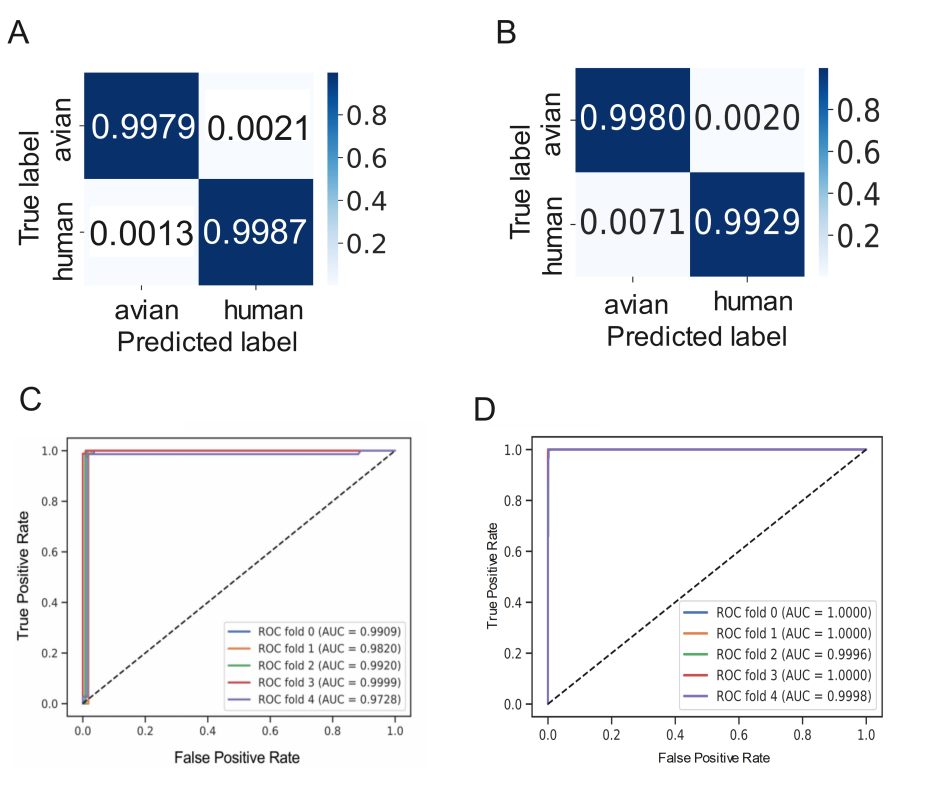

Supplement: nwaf396_Supplemental_Files [file nwaf396_supplemental_files.zip › Supplementary Figure 13.tif]

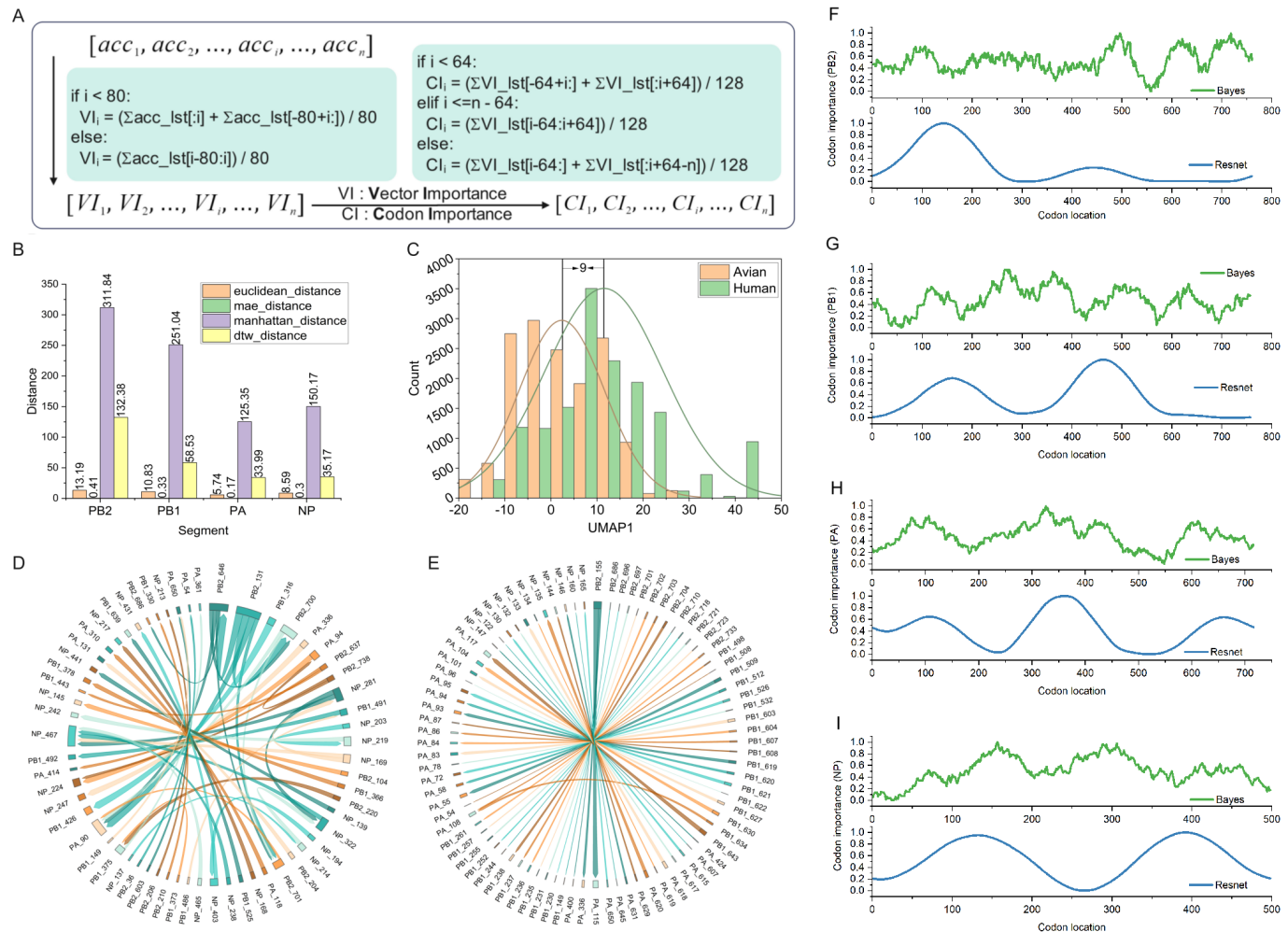

Supplement: nwaf396_Supplemental_Files [file nwaf396_supplemental_files.zip › Supplementary Figure 14.tif]

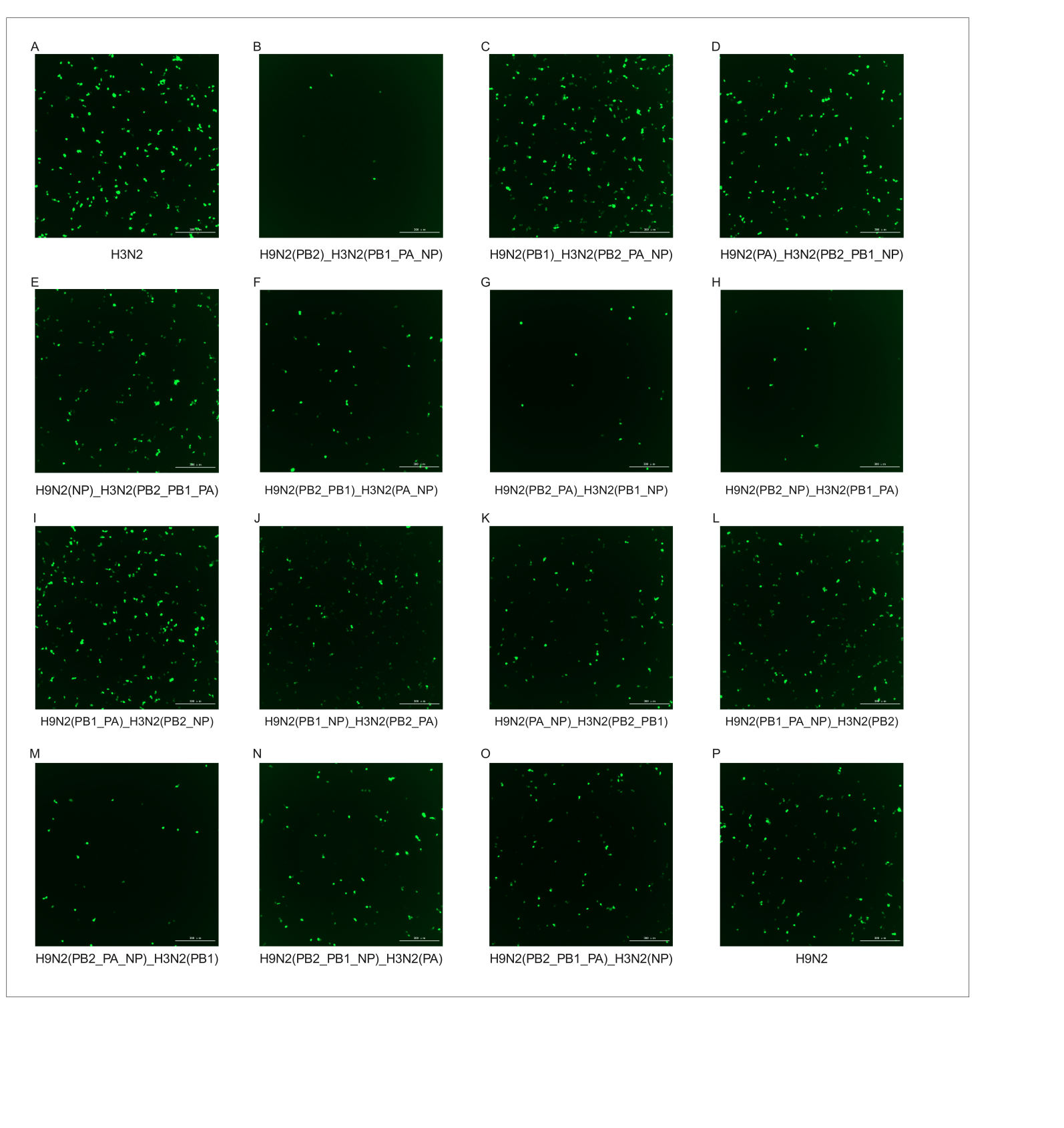

Supplement: nwaf396_Supplemental_Files [file nwaf396_supplemental_files.zip › Supplementary Figure 15.tif]

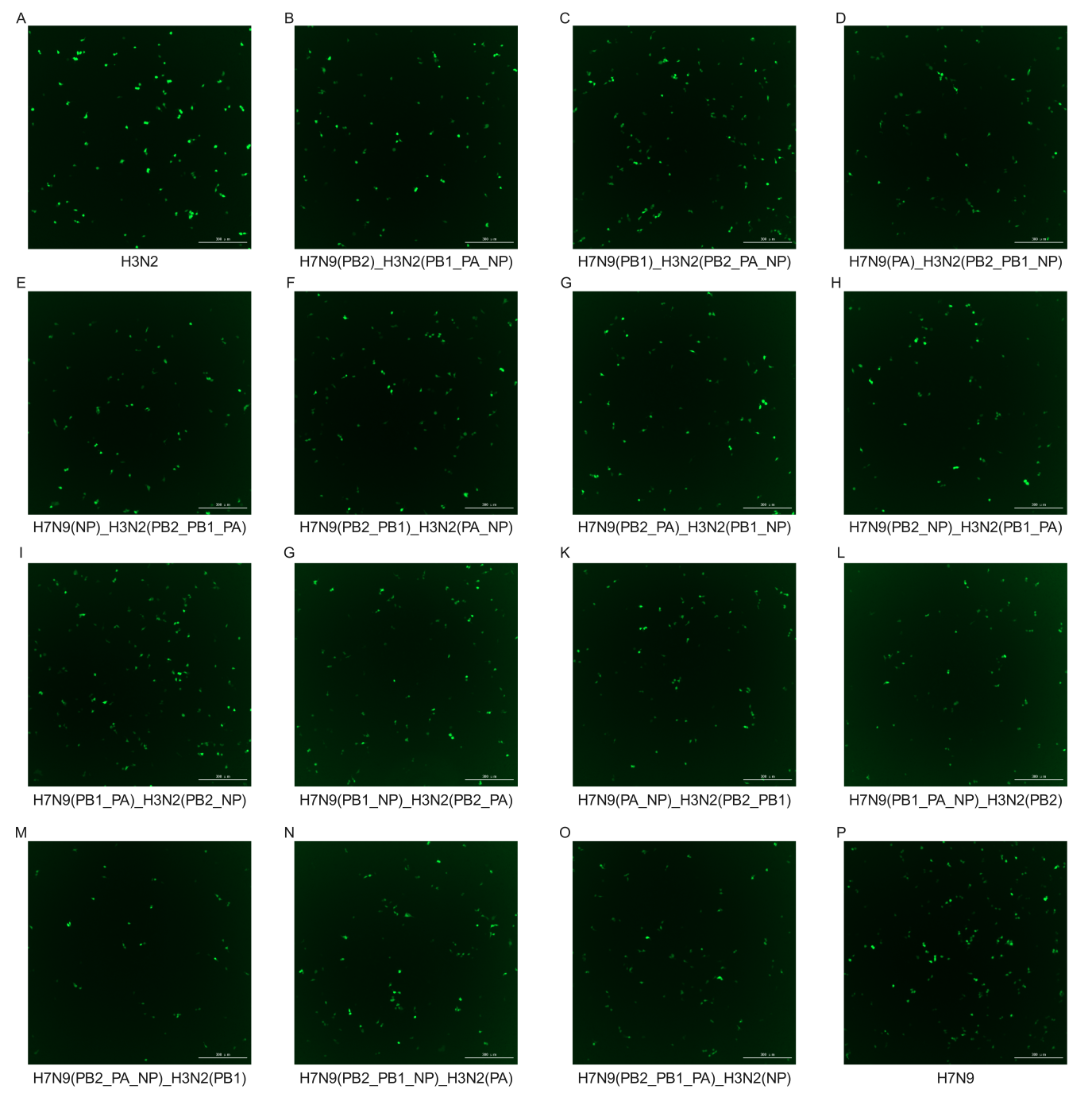

Supplement: nwaf396_Supplemental_Files [file nwaf396_supplemental_files.zip › Supplementary Figure 16.tif]

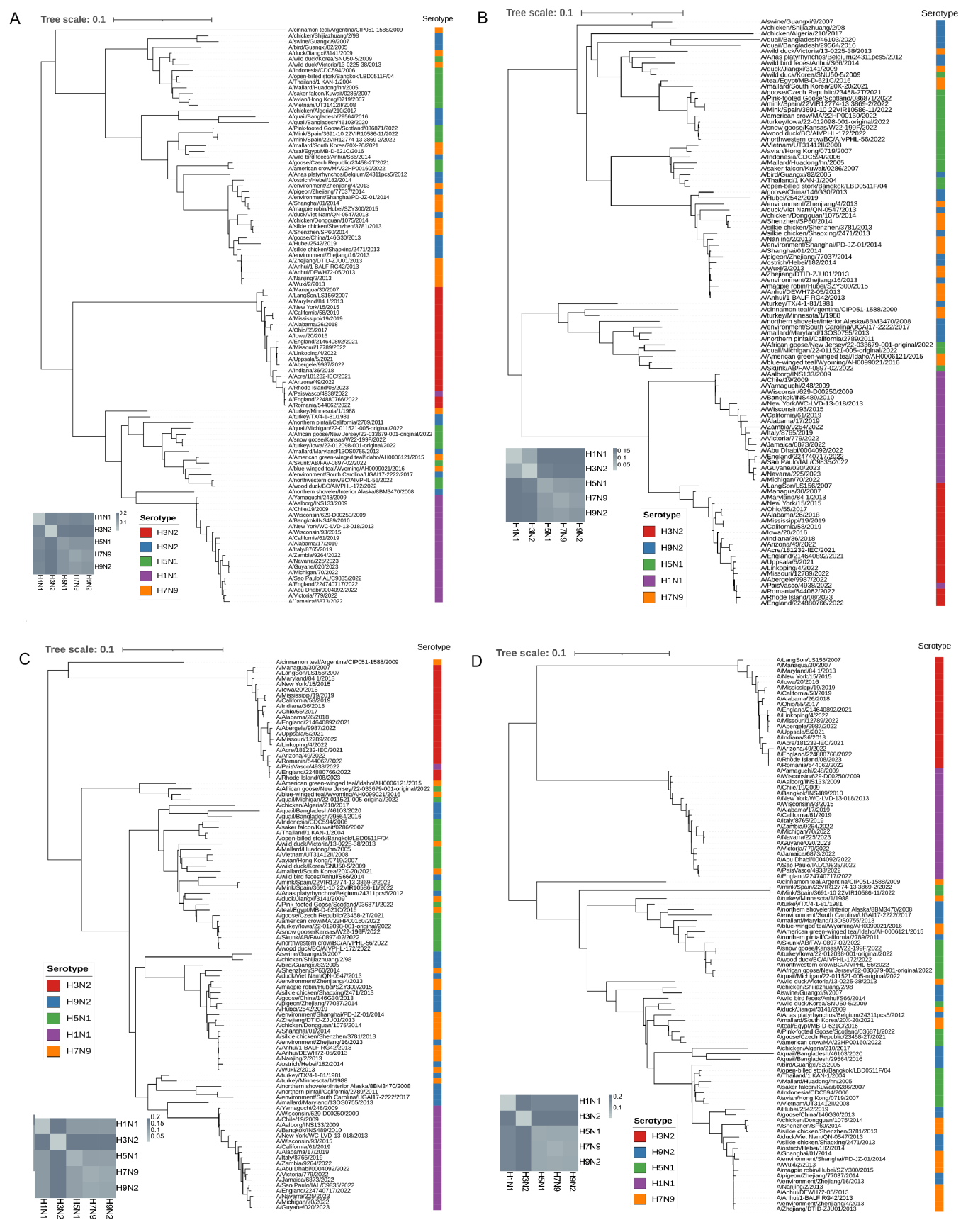

Supplement: nwaf396_Supplemental_Files [file nwaf396_supplemental_files.zip › Supplementary Figure 17.tif]

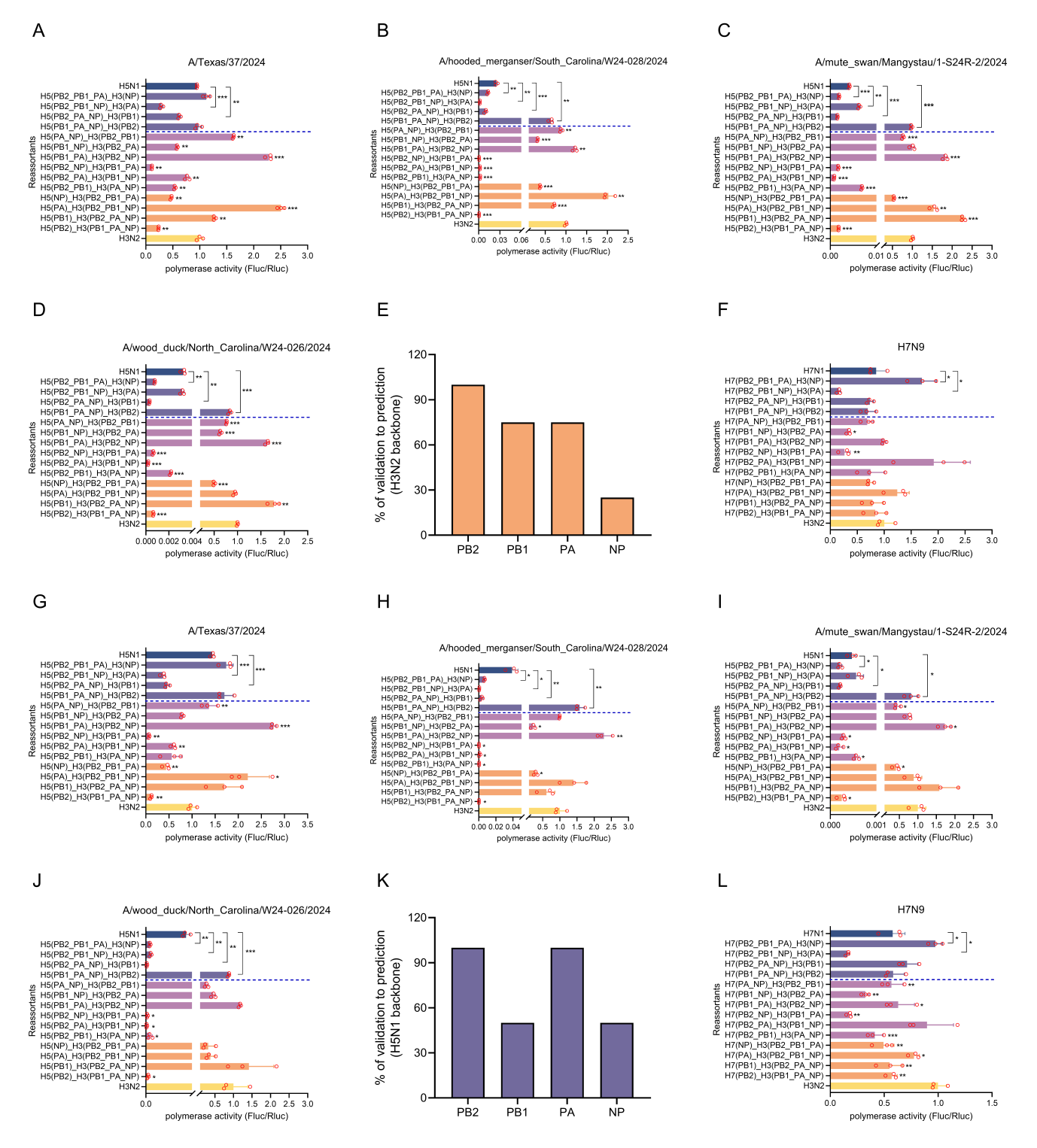

Supplement: nwaf396_Supplemental_Files [file nwaf396_supplemental_files.zip › Supplementary Figure 18.tif]

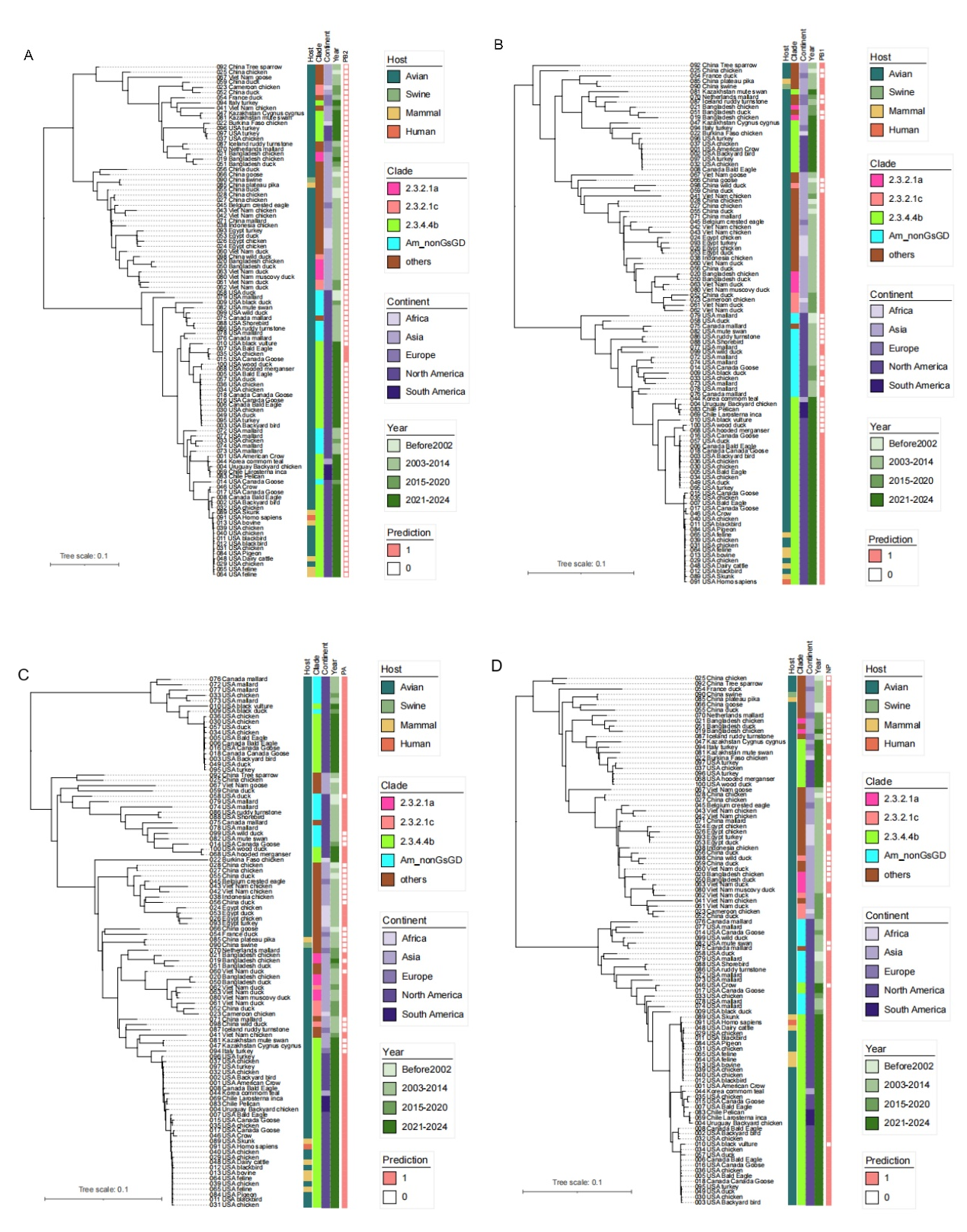

Supplement: nwaf396_Supplemental_Files [file nwaf396_supplemental_files.zip › Supplementary Figure 19.tif]

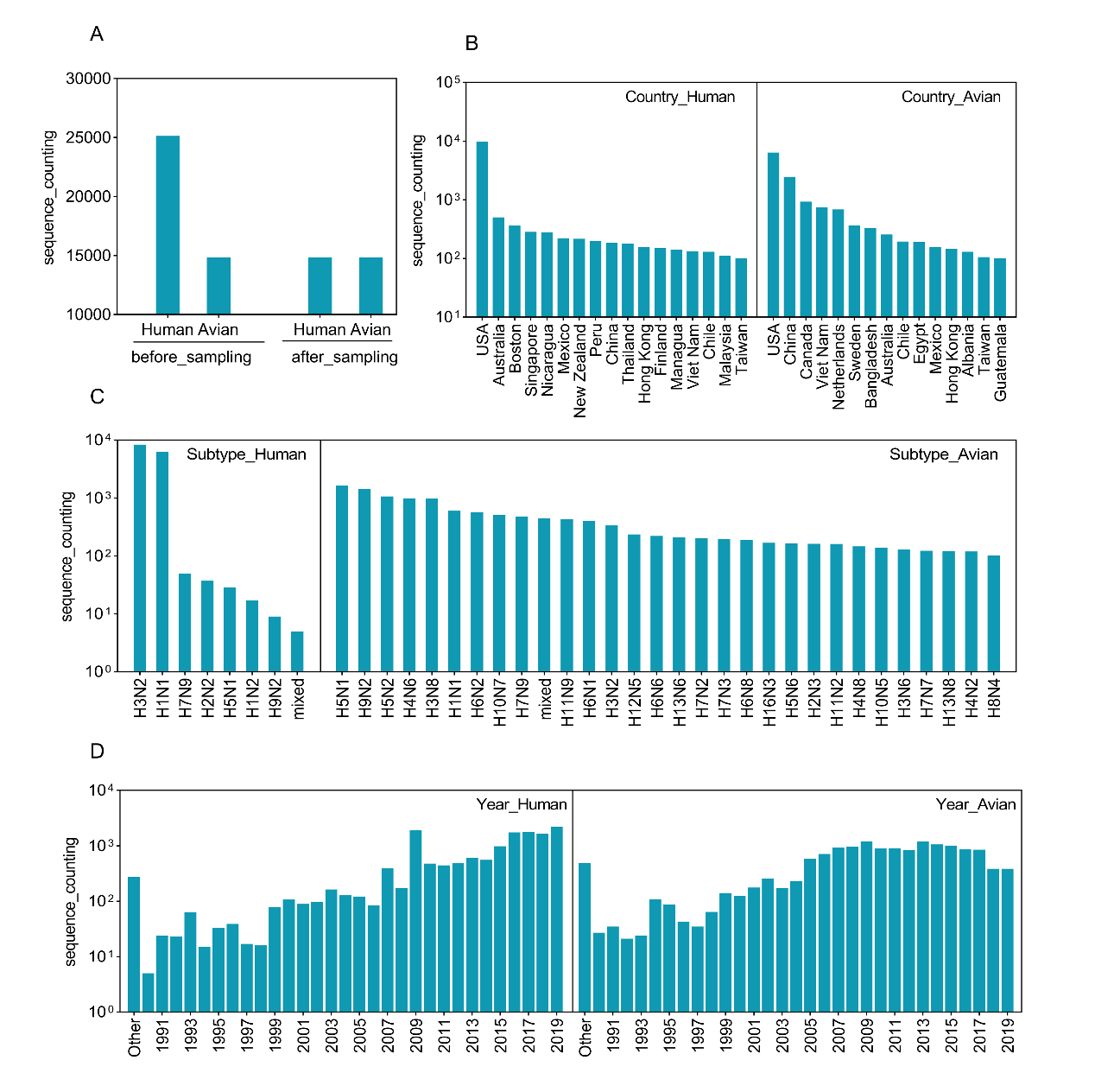

Supplement: nwaf396_Supplemental_Files [file nwaf396_supplemental_files.zip › Supplementary Figure 2.tif]

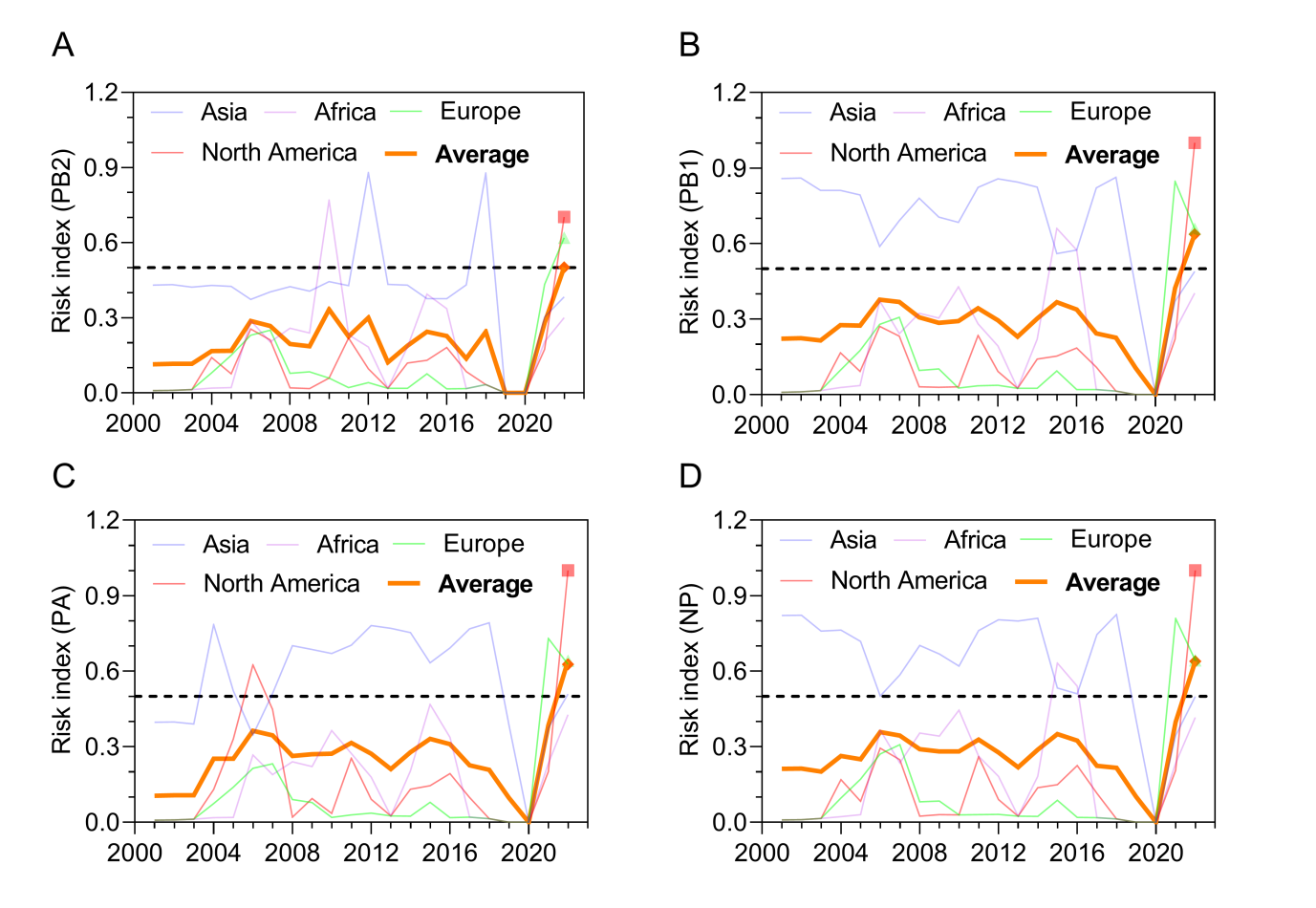

Supplement: nwaf396_Supplemental_Files [file nwaf396_supplemental_files.zip › Supplementary Figure 20.tif]

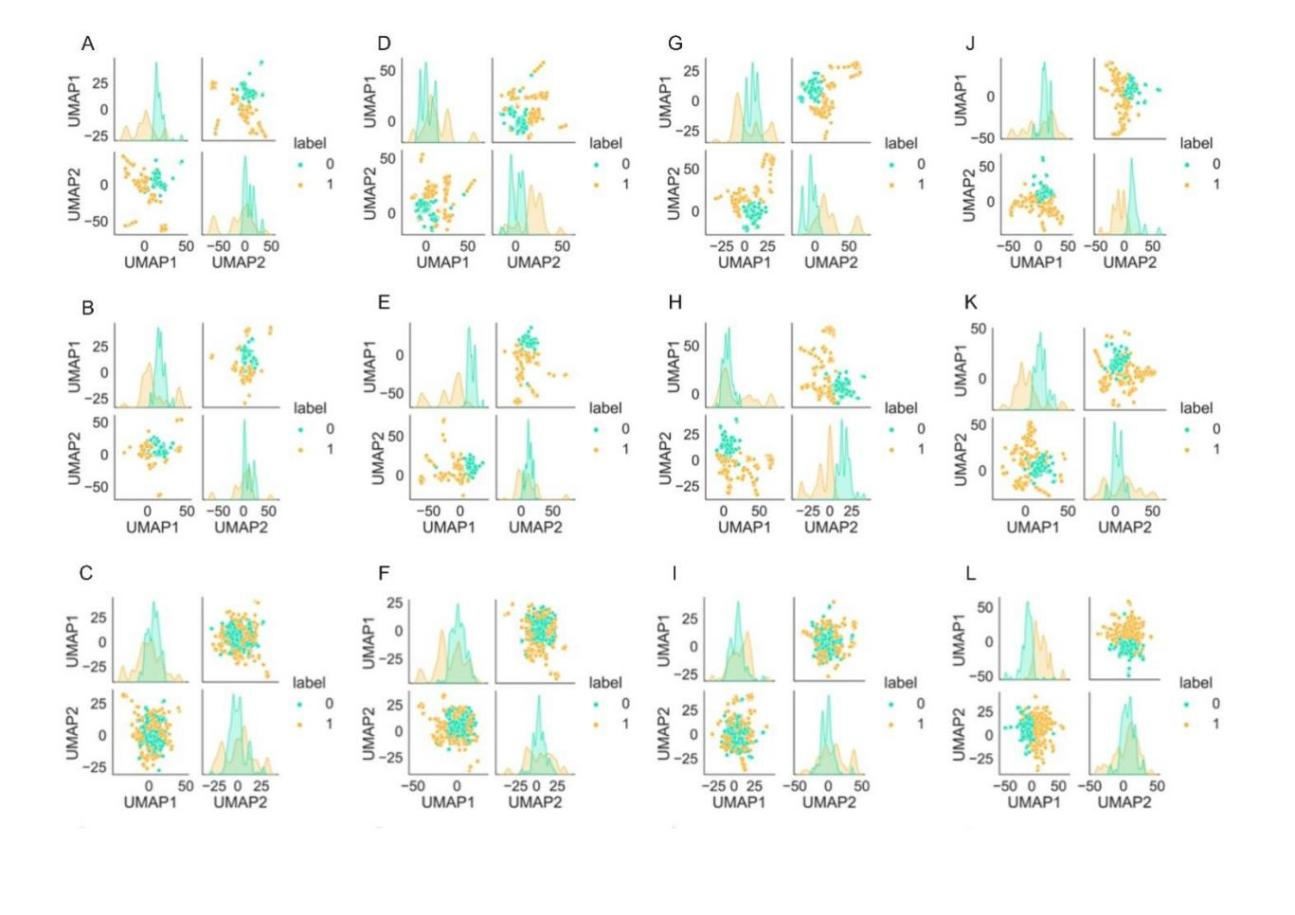

Supplement: nwaf396_Supplemental_Files [file nwaf396_supplemental_files.zip › Supplementary Figure 3.tif]

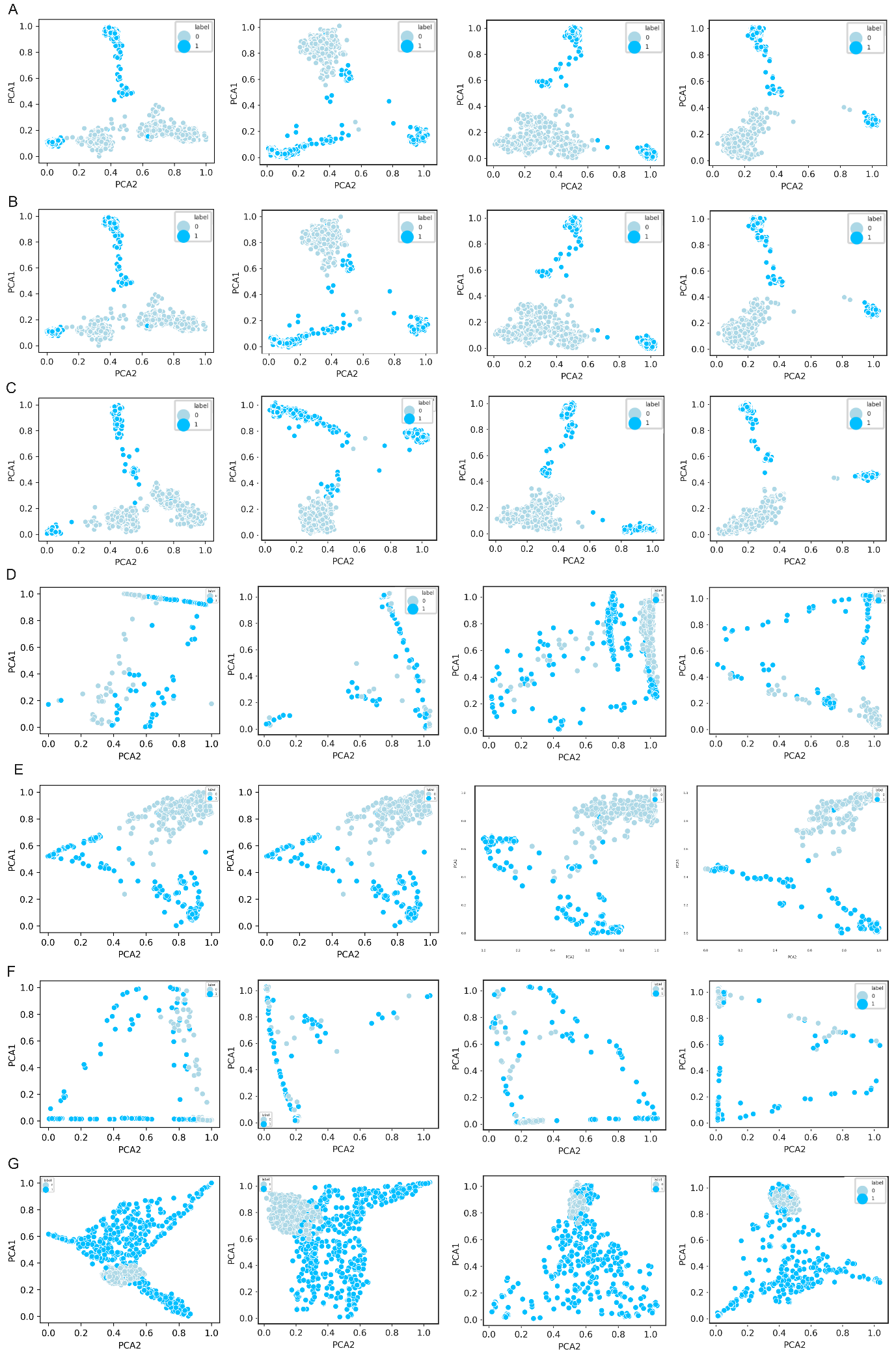

Supplement: nwaf396_Supplemental_Files [file nwaf396_supplemental_files.zip › Supplementary Figure 4.tif]

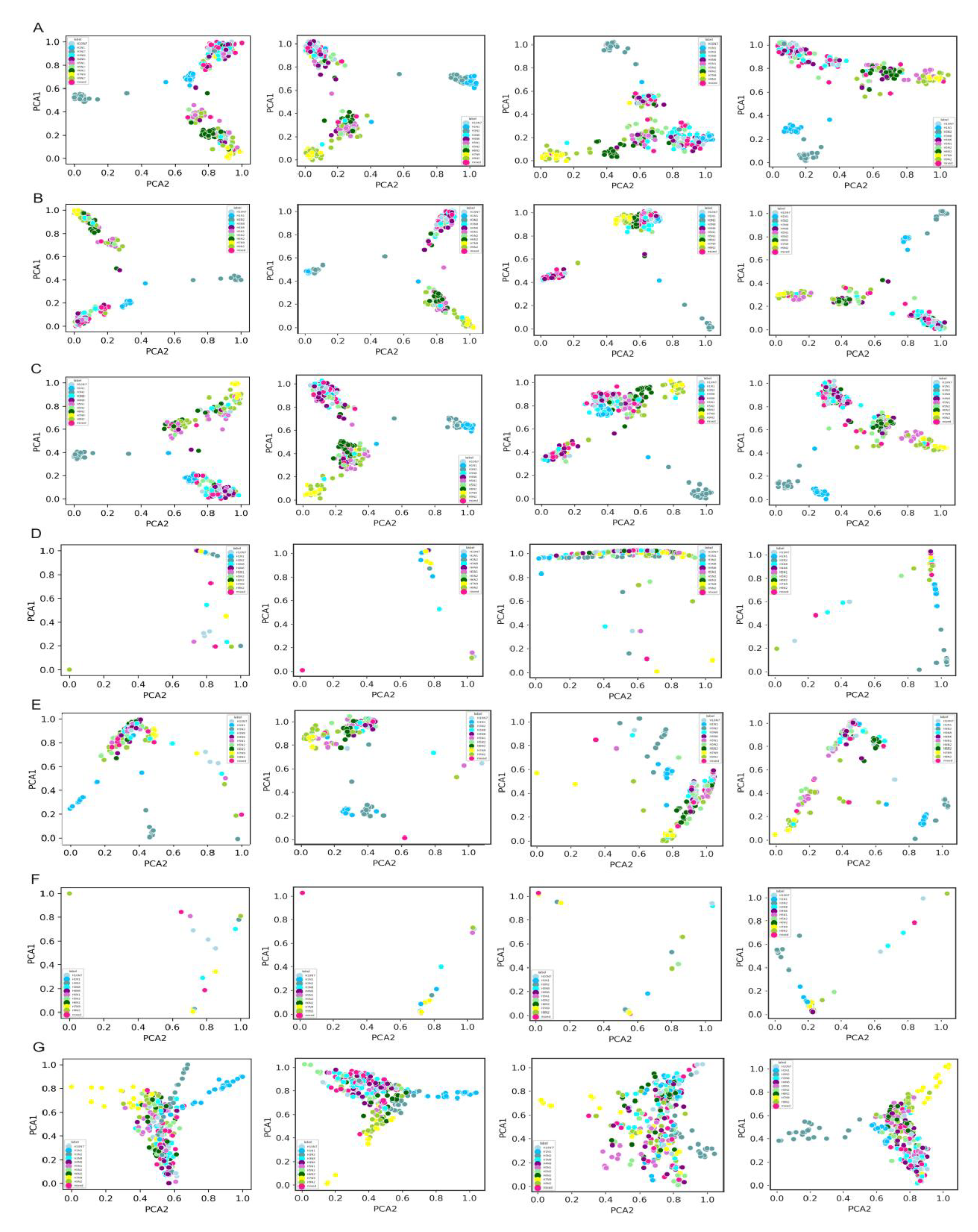

Supplement: nwaf396_Supplemental_Files [file nwaf396_supplemental_files.zip › Supplementary Figure 5.tif]

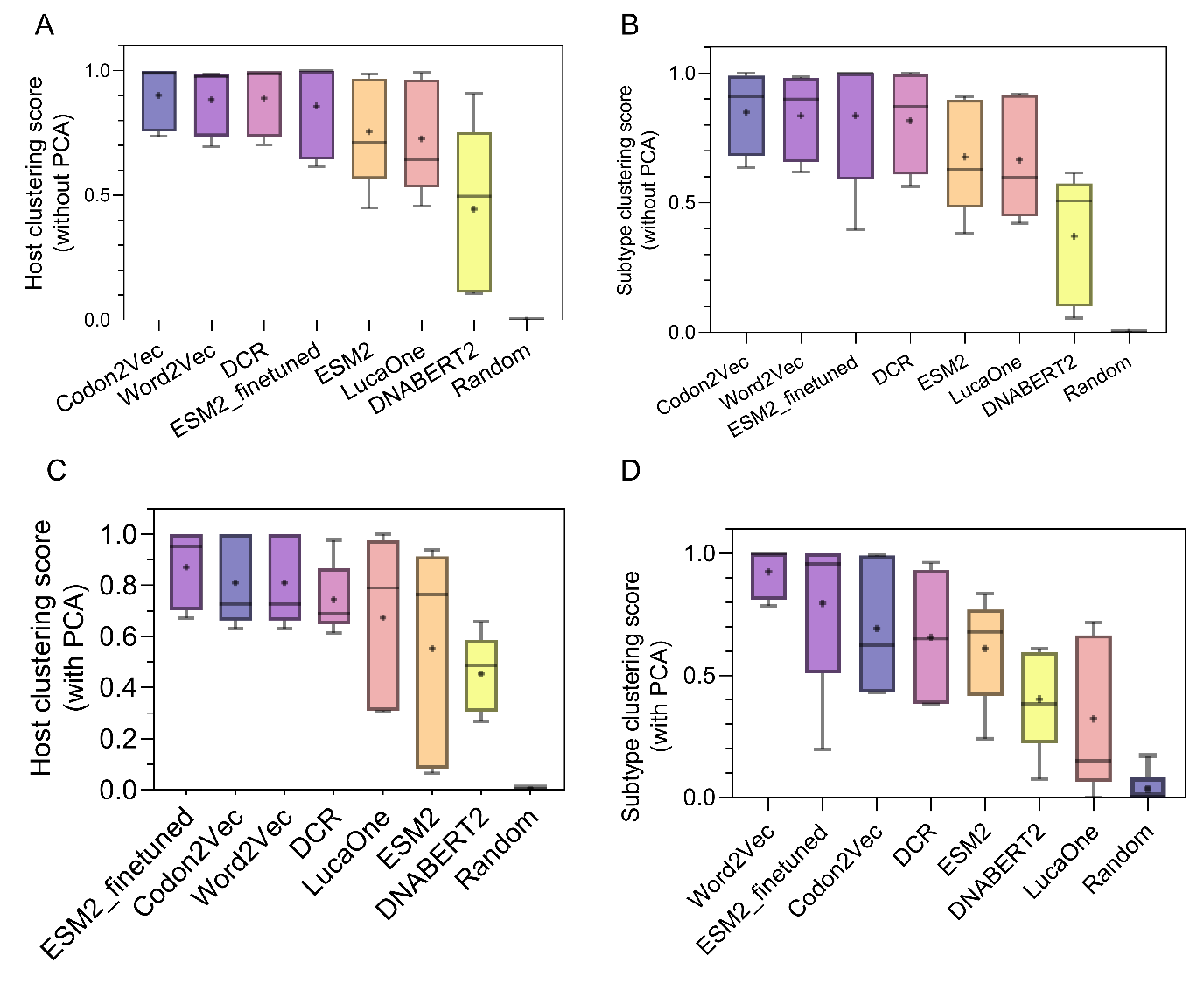

Supplement: nwaf396_Supplemental_Files [file nwaf396_supplemental_files.zip › Supplementary Figure 6.tif]

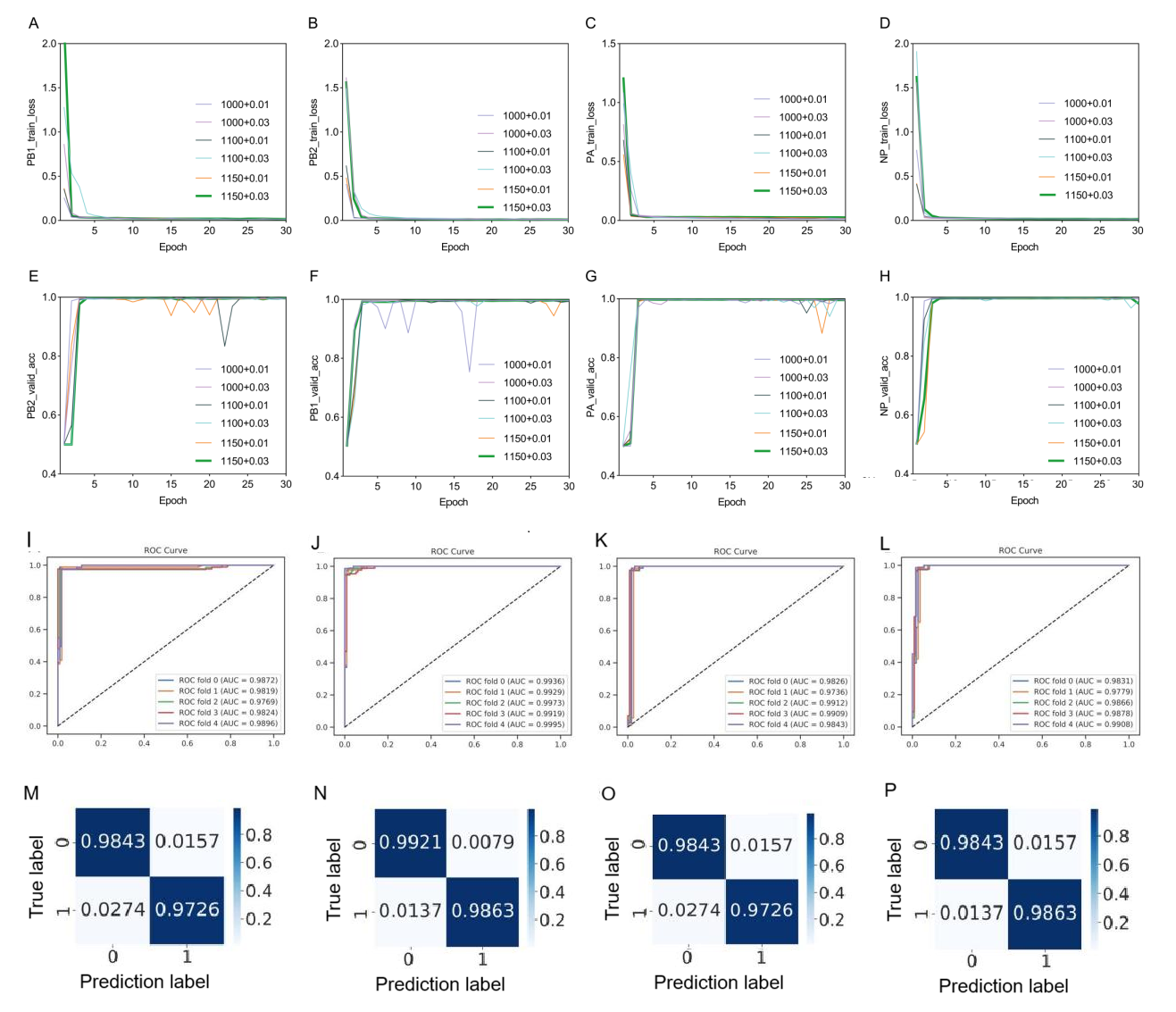

Supplement: nwaf396_Supplemental_Files [file nwaf396_supplemental_files.zip › Supplementary Figure 7.tif]

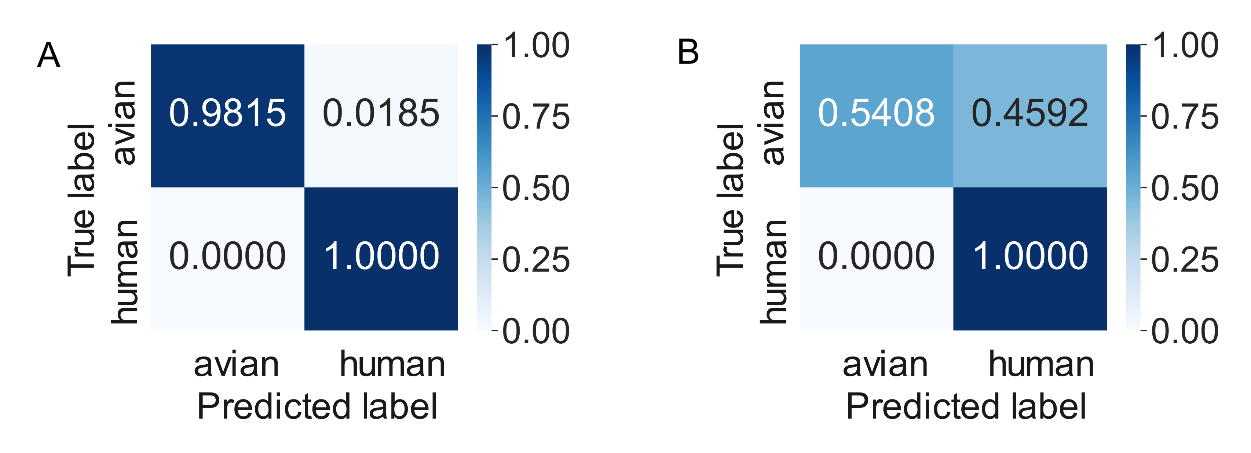

Supplement: nwaf396_Supplemental_Files [file nwaf396_supplemental_files.zip › Supplementary Figure 8.tif]

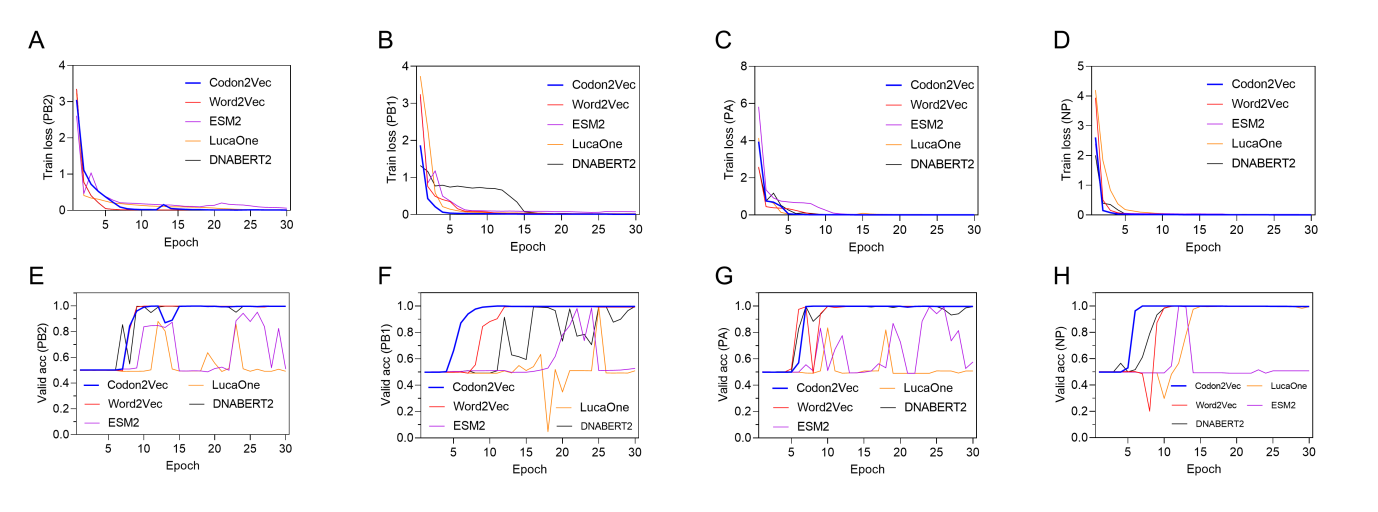

Supplement: nwaf396_Supplemental_Files [file nwaf396_supplemental_files.zip › Supplementary Figure 9.tif]
